# Randomised Double-Blind Comparison of Placebo and Active Drugs for Effects on Risks Associated with Blood Pressure Variability in the Systolic Hypertension in Europe Trial

**DOI:** 10.1371/journal.pone.0103169

**Published:** 2014-08-04

**Authors:** Azusa Hara, Lutgarde Thijs, Kei Asayama, Lotte Jacobs, Ji-Guang Wang, Jan A. Staessen

**Affiliations:** 1 Studies Coordinating Centre, Research Unit Hypertension and Cardiovascular Epidemiology, KU Leuven Department of Cardiovascular Diseases, University of Leuven, Leuven, Belgium; 2 Department of Hygiene and Public Health, Teikyo University School of Medicine, Tokyo, Japan; 3 Center for Epidemiological Studies and Clinical Trials, Ruijin Hospital, Shanghai Jiaotong University School of Medicine, Shanghai, China; 4 Department of Epidemiology, Maastricht University, Maastricht, the Netherlands; Hospital de Clínicas de Porto Alegre, Brazil

## Abstract

**Background:**

In the Systolic Hypertension in Europe trial (NCT02088450), we investigated whether systolic blood pressure variability determines prognosis over and beyond level.

**Methods:**

Using a computerised random function and a double-blind design, we randomly allocated 4695 patients (≥60 years) with isolated systolic hypertension (160–219/<95 mm Hg) to active treatment or matching placebo. Active treatment consisted of nitrendipine (10–40 mg/day) with possible addition of enalapril (5–20 mg/day) and/or hydrochlorothiazide (12.5–25.0 mg/day). We assessed whether on-treatment systolic blood pressure level (SBP), visit-to-visit variability independent of the mean (VIM) or within-visit variability (WVV) predicted total (*n* = 286) or cardiovascular (*n* = 150) mortality or cardiovascular (*n* = 347), cerebrovascular (*n* = 133) or cardiac (*n* = 217) endpoints.

**Findings:**

At 2 years, mean between-group differences were 10.5 mm Hg (p<0.0001) for SBP, 0.29 units (p = 0.20) for VIM, and 0.07 mm Hg (p = 0.47) for WVV. Active treatment reduced (p≤0.048) cardiovascular (−28%), cerebrovascular (−40%) and cardiac (−24%) endpoints. In analyses dichotomised by the median, patients with low *vs*. high VIM had similar event rates (p≥0.14). Low *vs*. high WVV was not associated with event rates (p≥0.095), except for total and cardiovascular mortality on active treatment, which were higher with low WVV (p≤0.0003). In multivariable-adjusted Cox models, SBP predicted all endpoints (p≤0.0043), whereas VIM did not predict any (p≥0.058). Except for an inverse association with total mortality (p = 0.042), WVV was not predictive (p≥0.15). Sensitivity analyses, from which we excluded blood pressure readings within 6 months after randomisation, 6 months prior to an event or both were confirmatory.

**Conclusions:**

The double-blind placebo-controlled Syst-Eur trial demonstrated that blood-pressure lowering treatment reduces cardiovascular complications by decreasing level but not variability of SBP. Higher blood pressure level, but not higher variability, predicted risk.

**Trial Registration:**

ClinicalTrials.gov NCT02088450

## Introduction

Whether blood pressure variability determines prognosis over and beyond level needs further clarification. Recent publications [1]–[4], reviewed elsewhere [5], [6] suggested that clinicians might reduce stroke incidence more by targeting systolic blood pressure variability along with level, preferentially using calcium-channel blockers [1]–[4], which might result in less blood pressure variability than other antihypertensive drugs classes. These recommendations, not endorsed by current guidelines [7], largely originated from observational population studies [8], [9] or from clinical trials [2] or cohort studies that exclusively enrolled high-risk patients with diabetes mellitus [10], [11], a history of stroke or transient ischaemic attack [2], or renal failure [12]–[14]. Other methodological issues that might have confounded the issue are categorisation of continuous variability measures for risk prediction [2], [9], the application of variability indexes that are dependent on blood pressure level [2], [9], and the limitation of endpoints to mortality [9], [13].

The double-blind Systolic Hypertension in the Elderly Trial (Syst-Eur) demonstrated that among older patients with isolated systolic hypertension, antihypertensive drug treatment starting with the calcium-channel blocker nitrendipine reduced the rate of stroke and cardiovascular complications [15], [16]. By analysing the Syst-Eur database, we investigated in a double-blind fashion whether placebo *vs*. active treatment starting with a dihydropyridine calcium-channel blocker differentially affected visit-to-visit and within-visit blood pressure variability and whether visit-to-visit or within-visit variability had an additive role to blood pressure level in predicting outcome.

## Methods

The protocol for this trial and supporting CONSORT checklist are available as supporting information; see Protocol S1 and Checklist S1.

### Ethics Statement

The protocol of this trial [16], [17] and the procedure to obtain informed consent were approved by the ethics committees of the University of Leuven and the 198 participating centres. We used the principles outlined in the Helsinki declaration [18]. At enrolment in the run-in period of the trial, all patients provided written informed consent. Investigators confirmed that they had obtained consent from patients in a checklist of inclusion and exclusion criteria, which they had to submit to the coordinating office before a patient could be randomised.

### Data Availability Statement

Bayer AG funded investigator-initiated Syst-Eur trial (1988-1997). The corresponding author owns the data and funding by Bayer does therefore in no way alter our adherence to PLOS ONE policies on sharing data and materials.

### Selection of patients

**Figure 1:**
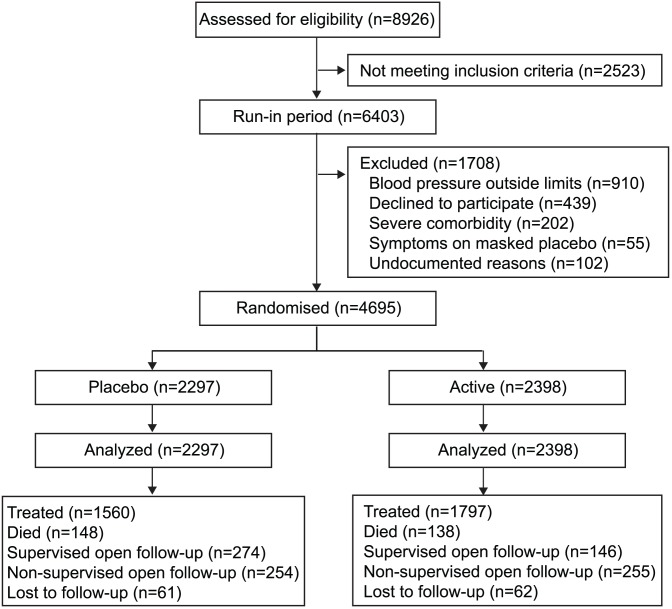
Trial profile.

Previous publications describe the Syst-Eur protocol, inclusion and exclusion criteria, and procedures for recruitment and randomisation [15]–[17]. Eligible patients were at least 60 years old. On masked placebo, during the run-in phase, their sitting systolic blood pressure ranged from 160 mm Hg to 219 mm Hg, their sitting diastolic blood pressure was below 95 mm Hg, and their standing systolic blood pressure was at least 140 mm Hg. All patients consented to be enrolled. The blood pressure determining eligibility was the average of six sitting and six standing readings, two in each position at three baseline visits, 1 month apart. Of 8926 screened patients, 6403 (71.7%) entered the run-in period, and 4695 (52.6%) were randomised (figure 1).

### Treatment and follow-up procedures

After stratification by centre, sex, and previous cardiovascular complications, eligible patients were randomly assigned to treatment with active medication or placebo. A computerised random function generated at the study coordinating centre in Leuven without block size restriction ensured random allocation of patients at the 198 participating centres. The study medications were stepwise titrated and combined to reduce sitting systolic blood pressure by 20 mm Hg or more to levels below 150 mm Hg. Active treatment was initiated with nitrendipine (10–40 mg/day). If necessary, the dihydropyridine calcium-channel blocker was combined with or replaced by enalapril maleate (5–20 mg/day), hydrochlorothiazide (12.5–25 mg/day), or both drugs. Placebo tablets were identical to the study drugs with a similar schedule. Patients who withdrew from double-blind treatment proceeded to supervised open follow-up. During double-blind and supervised open follow-up, patients had their sitting blood pressure measured twice at 3-monthly intervals. The two measurements were averaged for analysis of blood pressure level. Terminal digit and number preference were monitored during the trial [19]. Patients for whom regular follow-up was not possible proceeded to non-supervised open follow-up that involved collecting information on vital status, occurrence of major endpoints and other events, and the use of antihypertensive medications at annual intervals. Patients without any report within the year before the trial stopped were counted as lost to follow-up.

### Definition of endpoints

The main endpoints included death, stroke, myocardial infarction, and congestive heart failure. Stroke, the primary endpoint, was a neurological deficit with symptoms continuing for more than 24 h or leading to death with no apparent cause other than vascular. Acute myocardial infarction required two of the following three disorders: typical chest pain, electrocardiographic changes, or increased cardiac enzymes, and did not include silent myocardial infarction. Congestive heart failure required the presence of three disorders: symptoms, such as dyspnoea, clinical signs, such as ankle oedema or crackles, and the necessity of treatment with diuretics, vasodilators, or antihypertensive drugs. Sudden death encompassed any death of unknown origin occurring immediately or within 24 h of the onset of acute symptoms, as well as unattended death for which no likely cause could be established by necropsy or medical history. Cardiac events included fatal and non-fatal heart failure, fatal and non-fatal myocardial infarction, and sudden death. The endpoint committee, which was unaware of the patients' treatment status, identified all major endpoints by reviewing the patients' files and other source documents, or by requesting detailed written information from the investigators, or by both approaches. The number of endpoints in the present analysis rests on the update published in 1999 [16].

### Sample size

Our original sample-size calculations assumed a rate of stroke in the placebo group of 17.0 events per 1000 patient-years. 15 000 patient-years (i.e., 3000 patients with an average follow-up of 5 years) were required to detect a 40% change in the overall stroke rate with a two-tailed significance of 1% and 90% power [17]. A one-year pilot trial was concluded on 30 September 1989, and showed that the protocol could be followed and that the logistics of the trial were feasible. The Ethics Committee therefore implemented the full study on 1 February 1990. On August 18, 1995, the projected number of patients had been recruited [15]. However, because in the early phase of the study the stroke rate in the placebo group was only 13.6 events per 1000 patient-years, the steering committee decided in January, 1996, to continue recruitment through 1996 or until at least 4000 patients had been randomly assigned treatment. The steering committee stopped the trial prematurely on February 18, 1997, after the second interim analysis, because of a significant benefit of active treatment on the incidence of stroke [15].

### Statistical analysis

For database management and statistical analysis, we used the SAS system, version 9.3 (SAS Institute Inc., Cary, NC). Significance was a two-tailed α-level of 0.05 or less. Means and proportions were compared using the large-sample z-test or ANOVA and Fisher's exact test, respectively.

#### Assessment of blood pressure level and variability

We limited our analyses to systolic blood pressure, because in middle-aged and older subjects it is a stronger risk factor than diastolic blood pressure is [20]. Henceforth, blood pressure refers to systolic blood pressure. We assessed associations between level and variability of blood pressure using Pearson correlation coefficients and changes in continuous measurements using a large sample z-test.

We determined blood pressure level (SBP) and variability separately for the run-in period in 4695 patients and after randomisation in 2297 and 2398 patients randomised to placebo or active treatment, respectively. We assessed visit-to-visit blood pressure variability from the variability independent of the mean (VIM) [2], the standard deviation (SD), the coefficient of variation (CV), the difference between maximum and minimum blood pressure (MMD) [2], and average real variability (ARV) [21]. To compute visit-to-visit variability, we averaged the two blood pressure readings obtained at each visit over the period of analysis. SD is the within-patient standard deviation of the blood pressure values. CV is SD divided by the mean. MMD is the absolute difference between the highest and lowest blood pressure recorded at any visit during follow-up. VIM is calculated as the SD divided by the mean to the power *x* and multiplied by the population mean to the power x [2]. The power x is obtained by fitting a curve through a plot of SD against mean using the model SD  =  a × mean*^x^*, where *x* was derived by non-linear regression analysis as implemented in the PROC NLIN procedure of the SAS package. ARV is the average of the absolute differences between blood pressure measurements obtained at consecutive visits. ARV averages the absolute blood pressure differences between consecutive visits and thereby accounts for the order according to which blood pressure was measured 
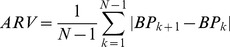
where k ranges from 1 to N–1, and N is the number of blood pressure readings [21].

Within-visit variability (WVV) is the absolute difference between the two blood pressure measurements at each visit averaged over the whole follow-up of each patient [1].

#### Analysis of outcome

We compared the incidence of the pre-defined endpoints between groups, using the log-rank test for rates and Kaplan-Meier survival function estimates. We used Cox regression to compute hazard ratios, while stratifying for centre and adjusting for randomisation group (if applicable), sex, age, body mass index, heart rate, serum total cholesterol, smoking and drinking, and history of cardiovascular disease or diabetes. Hazard ratios express the risk associated with a 1-SD increment in the level or variability of blood pressure. In our main analyses, we used all available blood pressure measurements during follow-up up to an event to compute blood pressure level and variability. In sensitivity analyses of VIM and WVV, we excluded the blood pressure readings obtained during the first 6 months or 1 year after randomisation [2], those obtained 6 months before an event, or those obtained 6 months after randomisation and 6 months before an event.

## Results

### Baseline characteristics

At randomisation, patients in the placebo (*n* = 2297) and active-treatment (*n* = 2398) groups were similar (p≥0.079) for distribution of sex, age, blood pressure, pulse rate, body mass index, serum cholesterol, use of tobacco and alcohol, previous cardiovascular complications, and antihypertensive treatment. Of 4695 participants, 3138 (66.8%) were women. Age averaged 70.2 (SD, 6.7) years. Sitting blood pressure was 173.8 (10.0) mmHg systolic and 85.5 (5.9) mmHg diastolic.

Table 1 lists the characteristics of participants by fourths of the SBP distribution at randomisation. Prevalence rates of previous cardiovascular complications, diabetes, and antihypertensive treatment before enrolment and mean values of age, body mass index, blood glucose, and serum creatinine increased (p≤0.019) across the SBP categories. During the run-in period (table 1), visit-to-visit blood pressure variability, as captured by SD, CV, MMD and ARV increased with higher category of SBP before randomisation (p<0.0001). VIM did not increase with higher run-in SBP (p = 0.084). In all 4695 patients, the correlation coefficients of VIM with SBP level were 0.01 (p = 0.59) during the run-in period and −0.01 (p = 0.75) during follow-up after randomisation. We therefore based our main analyses on VIM as index of visit-to-visit variability. WVV during the run-in period (table 1) increased with higher SBP (p<0.0001).

**Table 1:** Patient characteristics by fourths of the distribution of systolic blood pressure at randomisation.

Characteristic	Categories of systolic blood pressure				p
Limits, mm Hg	<166.2	166.3–171.7	171.8–179.2	≥179.3	
Number of subjects (%)					
All patients in category	1142	1206	1166	1181	
Women	764 (66.9)	785 (65.1)	763 (65.4)	826 (69.9)*	0.049
Smokers	78 (6.8)	85 (7.1)	83 (7.1)	97 (8.2)	0.58
Drinking alcohol	354 (31.0)	345 (28.6)	309 (26.5)	287 (24.3)	0.0023
Previous cardiovascular complications	270 (23.6)	358 (29.7)†	359 (30.8)	415 (35.1)*	<0.0001
Antihypertensive treatment	410 (36.1)	519 (43.1)‡	603 (51.7)§	662 (56.2)*	<0.0001
Diabetes mellitus	100 (8.8)	116 (9.6)	119 (10.2)	157 (13.3)*	0.0027
Mean (SD) of characteristic					
Age (years)	69.4 (6.5)	69.7 (6.3)	70.5 (6.8)†	71.4 (7.0)†	<0.0001
Body mass index (kg/m^2^)	26.9 (3.9)	27.0 (4.0)	26.9 (4.1)	27.3 (4.3)†	0.019
Heart rate (beats per minute)	72.6 (7.7)	73.1 (7.8)	73.4 (7.9)	73.4 (8.5)	0.074
Serum total cholesterol (mmol/l)	5.96 (1.14)	6.06 (1.17)*	6.03 (1.27)	6.01 (1.20)	0.21
Serum HDL cholesterol (mmol/l)	1.39 (0.43)	1.40 (0.42)	1.39 (0.45)	1.41 (0.45)	0.48
Blood glucose (mmol/L)	5.31 (1.36)	5.43 (1.45)*	5.43 (1.45)	5.58 (1.57)*	0.0002
Serum creatinine (µmol/L)	85.7 (17.8)	87.5 (18.1)*	88.5 (19.0)	89.9 (19.9)	<0.0001
Between-visit blood pressure variability					
Standard deviation (mm Hg)	4.9 (4.4)	5.6 (4.0)§	6.8 (5.0)§	8.2 (6.2)§	<0.0001
Coefficient of variation (%)	3.0 (2.7)	3.3 (2.4)†	3.9 (2.8)§	4.4 (3.2)§	<0.0001
Variability independent of mean (units)	6.1 (5.6)	6.2 (4.4)	6.6 (4.8)*	6.3 (4.7)	0.084
Maximum minus minimum (mm Hg)	9.3 (8.4)	10.7 (7.7)§	12.9 (9.6)§	15.6 (11.7)§	<0.0001
Average real variability (mm Hg)	6.0 (5.8)	7.1 (5.7)§	8.2 (6.4)§	10.0 (8.2)§	<0.0001
Within-visit variability (mm Hg)					
Averaged absolute difference	3.0 (2.4)	3.3 (2.6)*	3.4 (2.9)	3.9 (3.2)§	<0.0001

The p values denote the significance of the differences in prevalence rates or means across fourths of the distribution of systolic blood pressure at randomisation. The value of *x* to calculate VIM based on six blood pressure readings, two at each of three visits was 3.347. Significance of the difference with the adjacent lower fourth:

*p≤0.05;

† p≤0.01;

‡ p≤0.001;

§ p<0.0001.

### Changes in systolic blood pressure level and variability

The value of *x* to compute VIM was 0.847 in patients randomised to placebo and 0.981 in the active-treatment group. The correlation coefficients of SBP with VIM were −0.015 (p = 0.49) and −0.015 (p = 0.48) during follow-up on placebo or active treatment; the corresponding correlation coefficients with WVV were 0.12 and 0.071 (p≤0.0006), respectively.

#### Treatment status at 2 years

Among patients continuing double-blind medication, nitrendipine or matching placebo were the only treatments given at 2 years (median follow-up) in 597 (58.9%) of 1014 randomised to active treatment and in 343 (39.6%) of 866 placebo patients. Among the patients in open follow-up at 2 years, 65 (36.5%) of 178 randomly assigned to active treatment and 157 (58.1%) of 270 in the placebo group were on antihypertensive drugs. Antihypertensive treatment status was undocumented in 88 (49.4%) and 81 (30.0%) patients, respectively.

#### Blood pressure level and variability at 2 years

**Figure 2:**
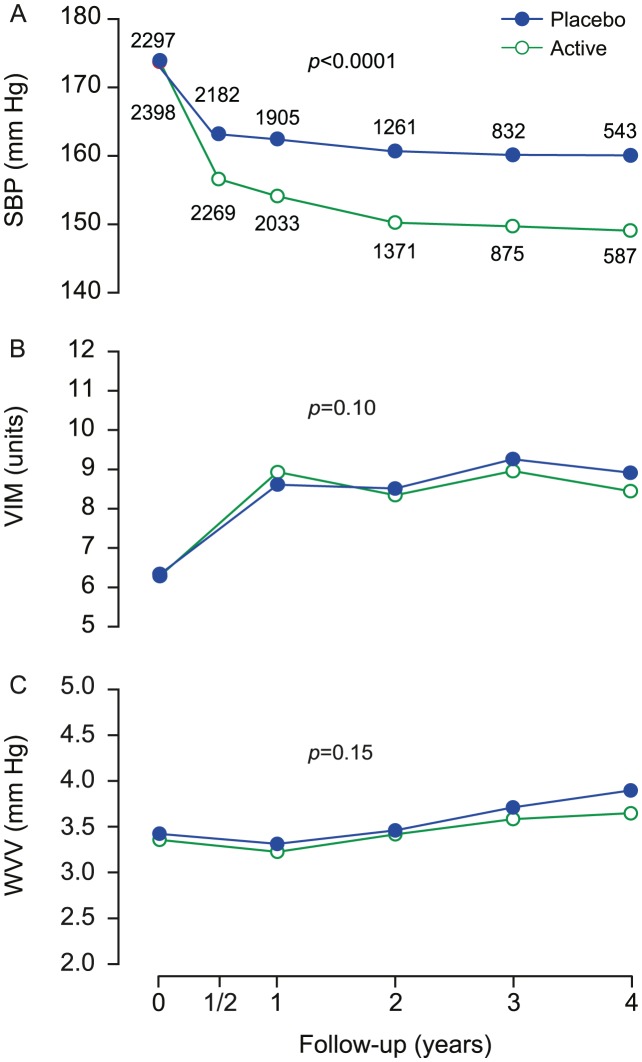
Systolic blood pressure level and variability at randomisation and during follow-up. SBP (A), VIM (B) and WVV (C) denote systolic blood pressure, visit-to-visit variability independent of the mean and within-visit variability. Values at randomisation and at annual intervals during follow-up were derived from at least six blood pressure readings, two at each of three consecutive visits. The blood pressure level at six months is the average of four blood pressure readings at two consecutive visits. The computation of variability requires at least three visits. Variability is therefore not plotted at 6 months. P values indicate the significance of the average between-group difference throughout follow-up.

At 2 years (figure 2), SBP had fallen (p<0.0001) by a mean (SD) of 13.6 (13.4) mm Hg and 24.0 (12.4) mm Hg in the placebo and active-treatment groups, respectively. VIM had increased (p<0.0001) by 1.99 (7.28) and 1.66 (6.56) units, respectively, whereas WVV had decreased (p = 0.044) by 0.17 (3.10) mm Hg in the active-treatment group but not significantly in placebo group, averaging 0.01 (3.24) mm Hg (p = 0.96). At 2 years, the mean between-group differences were 10.5 mm Hg (CI, 9.5 to 11.5; p<0.0001) for SBP, 0.29 units (CI, −0.15 to 0.74; p = 0.20) for VIM, and 0.073 mm Hg (CI, −0.13 to 0.27; p = 0.47) for WVV. At 4 years, the mean between-group differences were 11.0 mm Hg (CI, 9.4 to 12.6; p<0.0001) for SBP, 0.46 units (CI, −0.18 to 1.11; p = 0.16) for VIM, and 0.22 mm Hg (CI, −0.07 to 0.52; p = 0.14) for WVV.

#### Monotherapy vs. combination therapy

**Figure 3:**
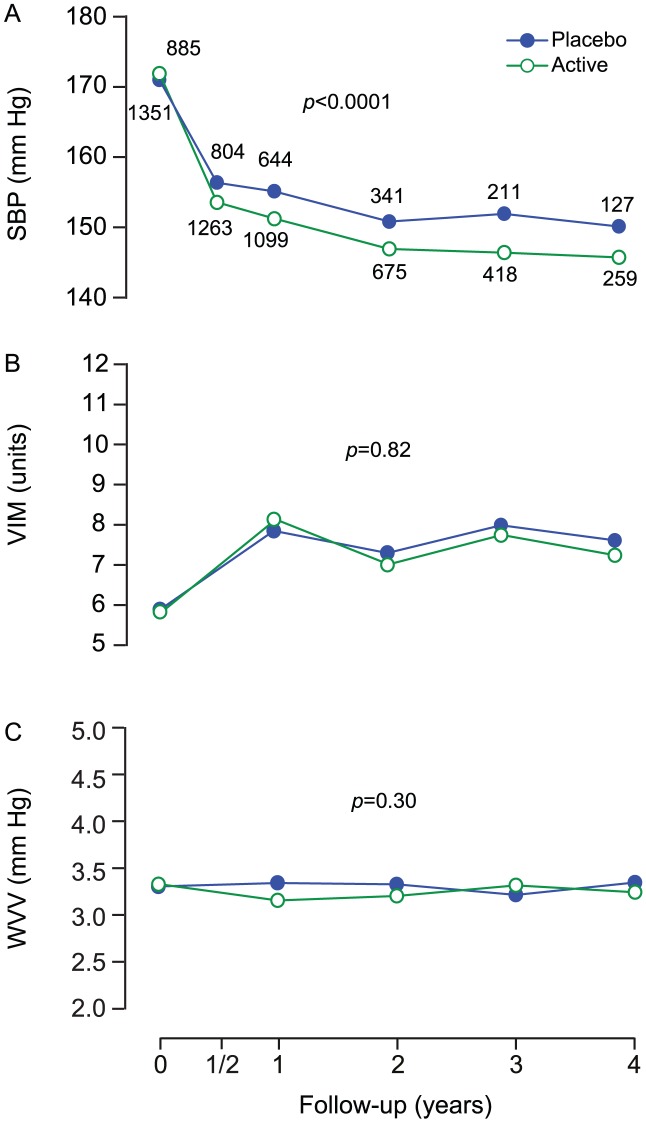
Blood pressure and variability at randomisation and during follow-up among patients staying on monotherapy. SBP (A), VIM (B) and WVV (C) denote systolic blood pressure, visit-to-visit variability independent of the mean and within-visit variability in 2236 patients staying on monotherapy with the first-line study medication. Values at randomisation and at annual intervals during follow-up are derived from at least six blood pressure readings, two at each of three consecutive visits. The blood pressure level at six months is the average of four blood pressure readings at two consecutive visits. The computation of variability requires at least three visits. Variability is therefore not plotted at 6 months. P values indicate the significance of the average between-group difference throughout follow-up.

The number of patient-years on monotherapy with first-line treatment was 1754.0 (31.0%) on nitrendipine placebo and 3107.4 (51.4%) on active nitrendipine. In patients remaining on monotherapy, the value of x to compute VIM was 3.354 in the run-in period and 0.058 and 0.659 in patients during follow-up on placebo (n = 885) or active nitrendipine (n = 1351), respectively. At 2 years, the mean between-group differences were 3.9 mm Hg (CI, 2.6 to 5.2; p<0.0001) for SBP, 0.29 units (CI, −0.32 to 0.91; p = 0.35) for VIM, and 0.12 mm Hg (CI, −0.19 to 0.44; p = 0.44) for WVV (figure 3).

**Figure 4:**
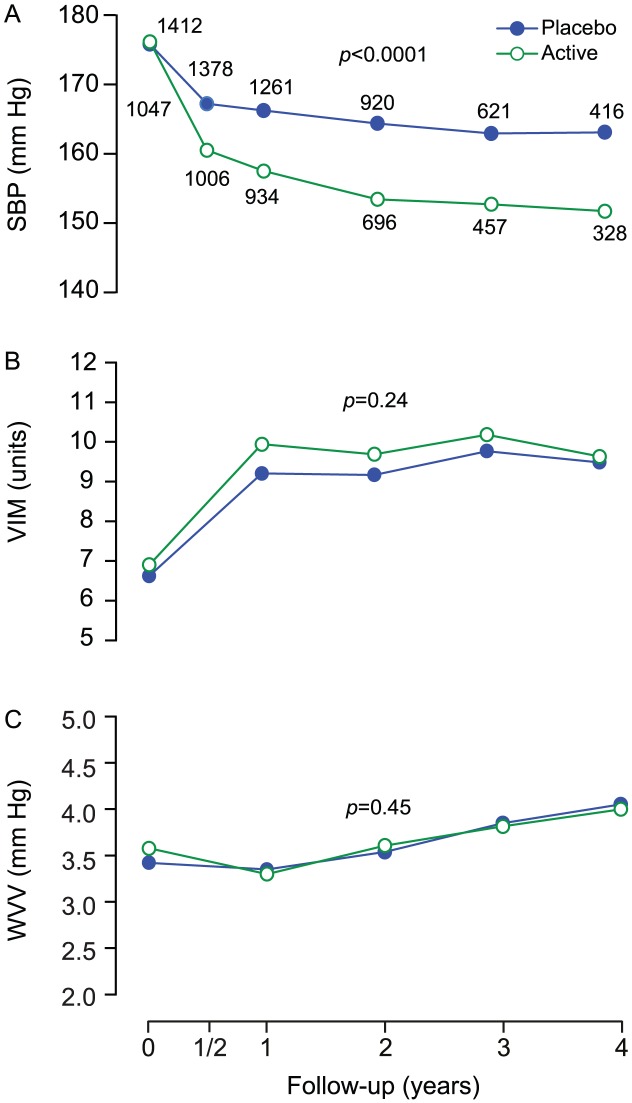
Blood pressure and variability at randomisation and during follow-up among patients proceeding to combination therapy. SBP (A), VIM (B) and WVV (C) denote systolic blood pressure, visit-to-visit variability independent of the mean and within-visit variability in 2459 patients proceeding to combination therapy. Values at randomisation and at annual intervals during follow-up are derived from at least six blood pressure readings, two at each of three consecutive visits. The blood pressure level at six months is the average of four blood pressure readings at two consecutive visits. The computation of variability requires at least three visits. Variability is therefore not plotted at 6 months. P values indicate the significance of the average between-group difference throughout follow-up.

Among patients proceeding to combination therapy, the value of *x* to compute VIM was 3.192 in the run-in period and 0.791 and 0.975 in patients during follow-up on placebo (n = 1412) or active nitrendipine (n = 1047), respectively. At 2 years, the mean between-group differences were 10.9 mm Hg (CI, 9.6 to 12.3; p<0.0001) for SBP, −0.52 units (CI, −1.13 to 0.09; p = 0.096) for VIM, and −0.069 mm Hg (CI, −0.33 to 0.20; p = 0.61) for WVV (figure 4).

### Incidence of endpoints

The number of accumulated patient-years was 5650.8 and 6045.1 in the placebo and active-treatment group, respectively. Table 2 lists the rates of the main endpoints. Active treatment reduced the incidence of the composite cardiovascular endpoint, fatal plus non-fatal stroke and fatal plus non-fatal cardiac events.

**Table 2:** Incidence of endpoints by randomisation group.

	Rate per 1000 patient-years		Relative difference with	
Endpoint	(number of endpoints)		rate in placebo group	
	Placebo	Active	% rate	p value
	(*n* = 2297)	(*n* = 2398)	(95% CI)	
**Mortality**				
Total	25.1 (148)	22.3 (138)	−11 (−29 to 13)	0.34
Cardiovascular	13.9 (82)	11.0 (68)	−21 (−43 to 9)	0.15
**Fatal plus non-fatal cardiovascular endpoints**				
All	34.7 (196)	25.0 (151)	−28 (−42 to −11)	0.0022
Stroke	14.0 (81)	8.5 (52)	−40 (−57 to −15)	0.0045
Cardiac endpoints	20.9 (120)	15.9 (97)	−24 (−42 to −0.3)	0.048
Heart failure	9.2 (53)	6.7 (41)	−27 (−51 to 10)	0.13
Myocardial infarction	8.4 (48)	6.4 (39)	−23 (−50 to 17)	0.22

Cardiac endpoints included fatal and non-fatal myocardial infarction, fatal and non-fatal heart failure, and sudden death. Significance was derived from the log-rank test.

**Figure 5:**
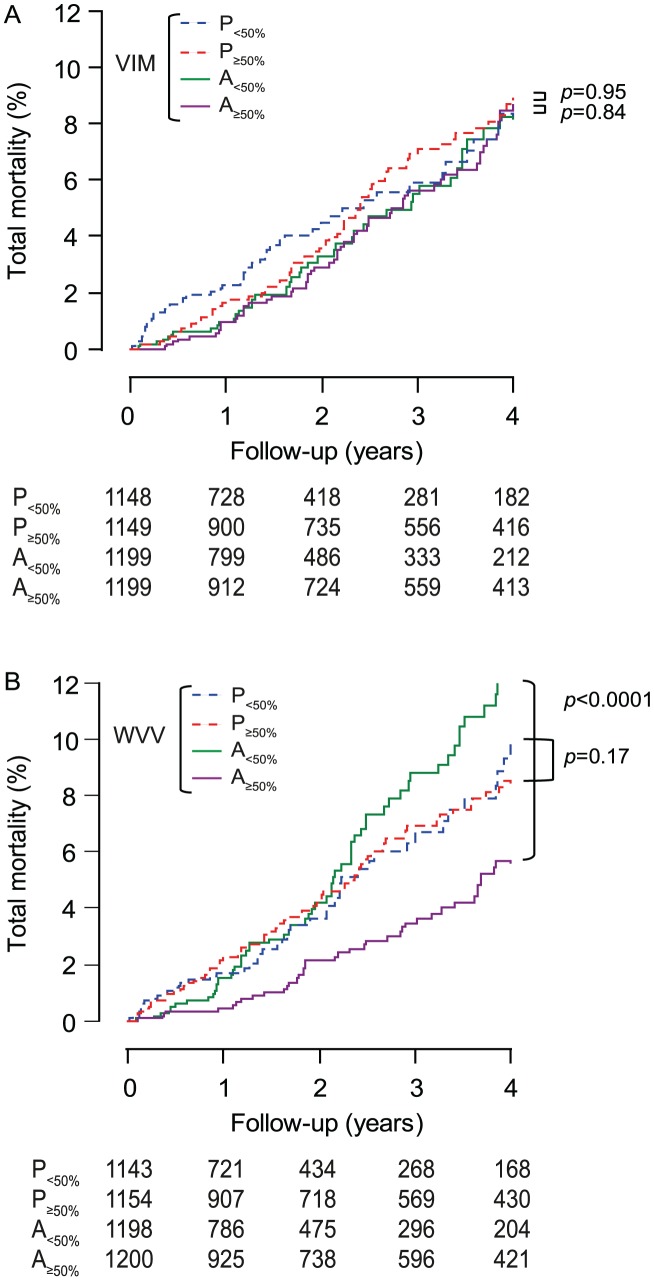
Incidence of total mortality by treatment group and median of blood pressure variability. VIM (A) and WVV (B) denote visit-to-visit variability independent of the mean and within-visit variability of systolic blood pressure. P and A indicate placebo and active treatment, respectively, and 50% refers to the median. On placebo (*n* = 2297) and active treatment (*n* = 2398), *x* values used to compute VIM were 0.822 and 0.628; medians were 9.36 and 9.60 units for VIM, and 2.83 and 2.91 mm Hg for WVV. P value indicates the significance of the log-rank test comparing low with high variability within each randomisation group.

**Figure 6:**
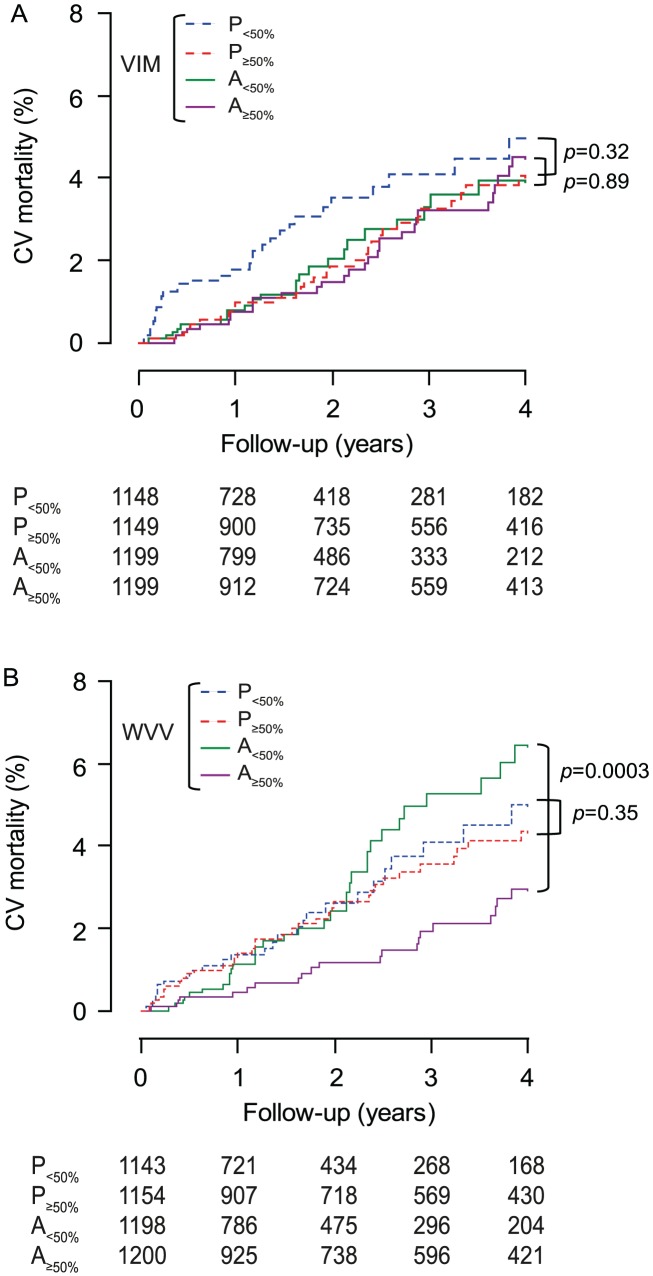
Incidence of cardiovascular mortality by treatment group and median of blood pressure variability. VIM (A) and WVV (B) denote visit-to-visit variability independent of the mean and within-visit variability of systolic blood pressure. P and A indicate placebo and active treatment, respectively, and 50% refers to the median. On placebo (*n* = 2297) and active treatment (*n* = 2398), *x* values used to compute VIM were 0.822 and 0.628; medians were 9.36 and 9.60 units for VIM and 2.83 and 2.91 mm Hg for WVV. P value indicates the significance of the log-rank test comparing low with high variability within each randomisation group.

**Figure 7:**
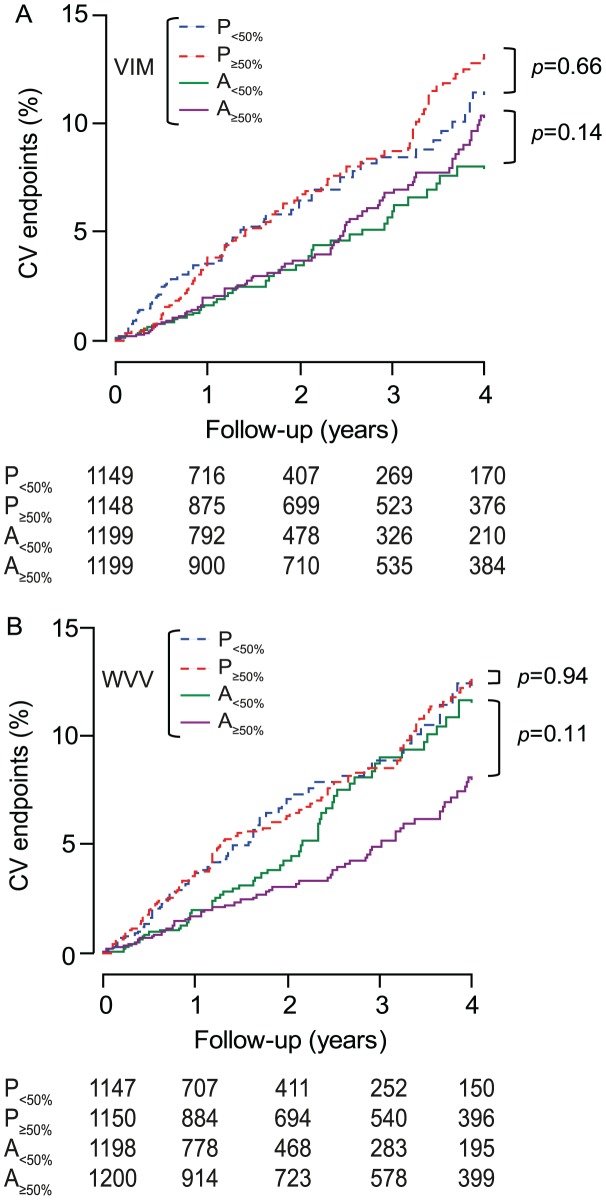
Incidence of the composite cardiovascular endpoint by treatment group and median of blood pressure variability. VIM and WVV denote visit-to-visit variability independent of the mean and within-visit variability. The number of analysed patients randomised to placebo and active treatment was 2297 and 2398, respectively. P and A indicate placebo and active treatment, respectively, and 50% refers to the median. On placebo and active treatment, *x* values used to compute VIM were 0.995 and 0.718; medians were 9.34 and 9.58 units for VIM and 2.82 and 2.91 mm Hg for WVV. P value indicates the significance of the log-rank test comparing low with high variability within each randomisation group.

Within each treatment group, in analyses dichotomised by the randomisation-group specific medians, low *vs*. high VIM was not associated with different rates (p≥0.14) of total or cardiovascular mortality (figures 5 and 6) or cardiovascular (figure 7), cerebrovascular or cardiac events. Low *vs*. high WVV was associated with higher rates of total and cardiovascular mortality (figures 5 and 6) on active treatment (p≤0.0003), but not on placebo (p≥0.17), while in both treatment groups incidence rates of cardiovascular (figure 7), cerebrovascular or cardiac events did not differ (p≥0.095) according to low *vs*. high WVV.

### Multivariable-adjusted analyses

**Figure 8:**
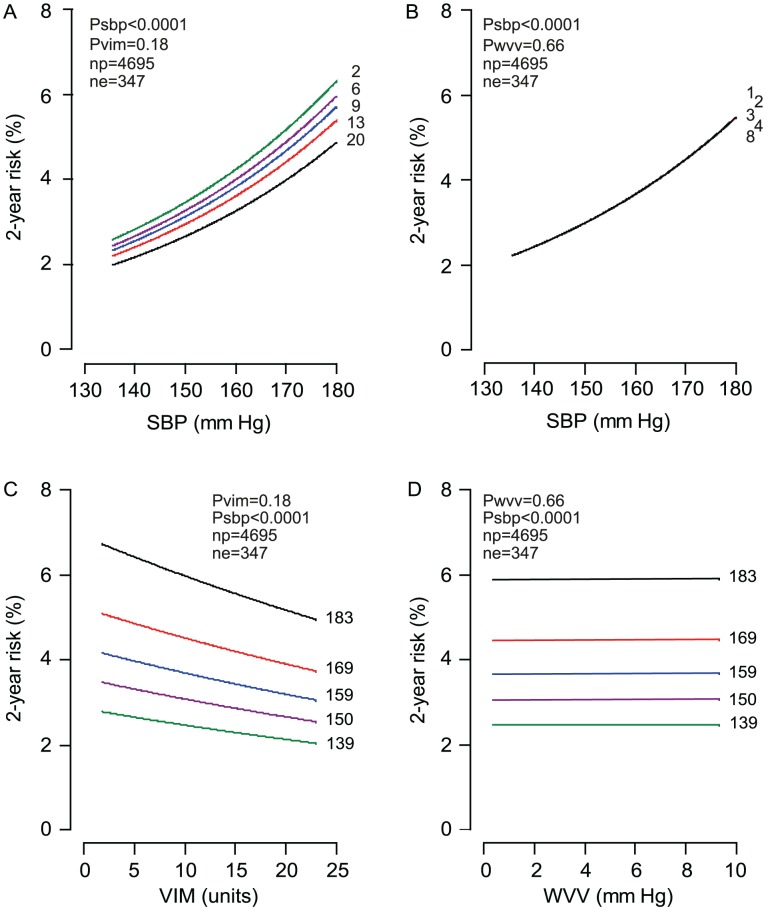
Two-year absolute risk of a cardiovascular endpoint in relation to level and variability. SBP, VIM and WVV denote systolic blood pressure, visit-to-visit variability independent of the mean and within-visit variability. The analyses account for randomisation group and were stratified for centre and standardised to the distributions (mean or ratio) of sex, age, body mass index, heart rate, cholesterol, smoking and drinking, and history of cardiovascular disease or diabetes. In panels A and B, the risk functions span the 5th to 95th percentile interval of SBP and are plotted for the 5th, 25th, 50th, 75th and 95th percentiles of VIM (panel A) or WVV (panel B). In panels C and D, the risk functions span the 5th to 95th percentile interval of VIM or WVV and are plotted for the 5th, 25th, 50th, 75th and 95th percentiles of SBP. The p values are for the independent effects of SBP (p_sbp_) and VIM (p_vim_) or WVV (p_wvv_). n_p_ and n_e_ indicate the number of patients at risk and the number of events.

In all patients, with stratification for centre and adjustments applied for randomisation group, sex, age, body mass index, heart rate, serum cholesterol, smoking and drinking, and history of cardiovascular disease or diabetes, the on-treatment SBP was a strong (p<0.0001) and consistent predictor of all endpoints under study and retained its significance when Cox models also included VIM (table 3) or WVV (table 4). SBP remained a strong prognosticator in analyses confined to patients on placebo or active treatment (tables 3 and 4). In multivariable-adjusted analyses (table 3), VIM was not associated with the risk of any endpoint (p≥0.058), except for a borderline significant but inverse association with the risk of cardiac endpoints in a model not including SBP (p = 0.047). WVV (table 4) did not predict any endpoint (p≥0.14) with exception of an inverse association (p = 0.042) with total mortality among actively treated patients in a model including SBP. Figure 8 shows that the absolute risk of a composite cardiovascular endpoint increased with level of SBP, but was not associated with VIM or WVV.

**Table 3:** Hazard ratios associated with level and visit-to-visit variability of systolic blood pressure during follow-up.

		Mortality		Fatal plus non–fatal events		
Predictor variable (SD)	Model	Total	Cardiovascular	Cardiovascular	Stroke	Cardiac
All patients (*n* = 4695)						
SBP (∼13.6 mm Hg)	…	1.35 (1.19–1.54)§	1.69 (1.40–2.04)§	1.53 (1.36–1.73)§	1.62 (1.33–1.97)§	1.51 (1.30–1.76)§
	VIM	1.35 (1.19–1.54)§	1.70 (1.41–2.05)§	1.53 (1.36–1.73)§	1.61 (1.32–1.97)§	1.51 (1.29–1.76)§
VIM (∼5.5 units)	…	0.94 (0.81–1.09)	0.82 (0.66–1.02)	0.91 (0.79–1.04)	1.04 (0.84–1.28)	0.84 (0.70–0.99)*
	SBP	0.94 (0.81–1.09)	0.81 (0.66–1.01)	0.91 (0.80–1.04)	1.01 (0.82–1.25)	0.86 (0.72–1.02)
Placebo (*n* = 2297)						
SBP (∼13.5 mm Hg)	…	1.29 (1.09–1.53)†	1.68 (1.30–2.18)§	1.46 (1.24–1.72)§	1.70 (1.27–2.26)‡	1.42 (1.16–1.73)‡
	VIM	1.28 (1.08–1.53)†	1.67 (1.29–2.16)§	1.45 (1.23–1.71)§	1.70 (1.27–2.27)‡	1.41 (1.15–1.72)‡
VIM (∼5.6 units)	…	0.90 (0.73–1.10)	0.75 (0.56–1.01)	0.86 (0.72–1.03)	0.97 (0.73–1.28)	0.80 (0.63–1.02)
	SBP	0.92 (0.74–1.13)	0.77 (0.57–1.04)	0.88 (0.73–1.05)	0.96 (0.72–1.28)	0.83 (0.65–1.05)
Active treatment (n = 2398)						
SBP (∼12.6 mm Hg)	…	1.46 (1.15–1.83)†	1.80 (1.29–2.50)‡	1.75 (1.42–2.15)§	1.69 (1.22–2.35)†	1.71 (1.30–2.26)‡
	VIM	1.46 (1.16–1.85)†	1.84 (1.32–2.56)‡	1.77 (1.44–2.18)§	1.75 (1.24–2.45)†	1.71 (1.30–2.26)‡
VIM (∼5.2 units)	…	0.95 (0.76–1.20)	0.86 (0.61–1.21)	0.91 (0.73–1.13)	1.00 (0.71–1.39)	0.87 (0.65–1.17)
	SBP	0.93 (0.74–1.18)	0.80 (0.56–1.15)	0.88 (0.70–1.10)	0.87 (0.60–1.26)	0.89 (0.66–1.19)

Level (SBP) and visit-to-visit variability independent of the mean (VIM) were calculated from all blood pressure readings available from randomisation until the occurrence of an endpoint or until the end of follow-up in patients without an event. Hazard ratios express the risk associated with a 1-SD increase in the predictor variable and were stratified for centre and adjusted for randomisation group (all patients), sex, age, body mass index, heart rate, cholesterol, smoking and drinking, and history of cardiovascular disease or diabetes. Model indicates which systolic index was entered into the models in addition to the studied predictor. SDs are approximate, because the number of blood pressure readings available for analysis differed slightly depending on the timing of endpoints. An ellipsis signifies that in addition to the studied predictor no additional systolic index was entered.

Significance of the estimates:

*P≤0.05;

† *P*≤0.01;

‡ *P*≤0.001;

§ p<0.0001.

**Table 4:** Hazard ratios associated with level and within-visit variability of systolic blood pressure during follow-up.

		Mortality		Fatal plus non–fatal events		
Predictor variable (SD)	Model	Total	Cardiovascular	Cardiovascular	Stroke	Cardiac
All patients (*n* = 4695)						
SBP (∼13.6 mm Hg)	…	1.35 (1.19–1.54)§	1.69 (1.40–2.04)§	1.53 (1.36–1.73)§	1.62 (1.33–1.97)§	1.51 (1.30–1.76)§
	WVV	1.36 (1.19–1.55)§	1.70 (1.41–2.05)§	1.54 (1.36–1.73)§	1.61 (1.32–1.97)§	1.52 (1.30–1.77)§
WVV (∼2.3 mm Hg)	…	0.94 (0.76–1.15)	0.91 (0.68–1.21)	1.02 (0.86–1.21)	1.10 (0.84–1.45)	1.00 (0.80–1.26)
	SBP	0.90 (0.73–1.11)	0.86 (0.65–1.14)	0.96 (0.81–1.15)	1.03 (0.78–1.36)	0.95 (0.76–1.19)
Placebo (*n* = 2297)						
SBP (∼13.5 mm Hg)	…	1.29 (1.09–1.53)†	1.68 (1.30–2.18)§	1.46 (1.24–1.72)§	1.70 (1.27–2.26)‡	1.42 (1.16–1.73)‡
	WVV	1.29 (1.09–1.54)†	1.70 (1.31–2.19)§	1.46 (1.24–1.72)§	1.67 (1.25–2.24)‡	1.43 (1.17–1.75)‡
WVV (∼2.4 mm Hg)	…	1.00 (0.73–1.37)	0.91 (0.60–1.38)	1.03 (0.80–1.33)	1.28 (0.87–1.88)	0.88 (0.61–1.26)
	SBP	0.96 (0.71–1.30)	0.87 (0.59–1.28)	0.96 (0.75–1.23)	1.16 (0.79–1.71)	0.85 (0.60–1.20)
Active treatment (n = 2398)						
SBP (∼12.6 mm Hg)	…	1.46 (1.15–1.83)†	1.80 (1.29–2.50)‡	1.75 (1.42–2.15)§	1.69 (1.22–2.35)†	1.71 (1.30–2.26)‡
	WVV	1.53 (1.21–1.95)‡	1.90 (1.36–2.67)‡	1.77 (1.44–2.18)§	1.69 (1.22–2.35)†	1.71 (1.29–2.26)‡
WVV (∼2.3 mm Hg)	…	0.78 (0.56–1.08)	0.83 (0.53–1.30)	0.97 (0.74–1.26)	0.90 (0.56–1.45)	1.11 (0.80–1.54)
	SBP	0.70 (0.49–0.99)*	0.69 (0.41–1.14)	0.89 (0.68–1.18)	0.89 (0.55–1.43)	1.02 (0.72–1.44)

Level (SBP) and within-visit variability (WVV) were calculated from all blood pressure readings available from randomisation until the occurrence of an endpoint or until the end of follow-up in patients without an event. Hazard ratios express the risk associated with a 1-SD increase in the predictor variable and were stratified for centre and adjusted for randomisation group (all patients), sex, age, body mass index, heart rate, cholesterol, smoking and drinking, and history of cardiovascular disease or diabetes. Model indicates which systolic index was entered into the models in addition to the studied predictor. SDs are approximate, because the number of blood pressure readings available for analysis differed slightly depending on the timing of endpoints. An ellipsis signifies that in addition to the studied predictor no additional systolic index was entered. Significance of the estimates:

*P≤0.05;

† *P*≤0.01;

‡ *P*≤0.001;

§ p<0.0001.

Analyses with a time-dependent adjustment for proceeding to combination therapy, produced results for VIM (table 5) and WVV (table 6) consistent with those presented in tables 3 and 4, respectively. Similarly, sensitivity analyses of VIM (table 7) and WVV (table 8), from which we excluded blood pressure measurements obtained within the first 6 or 12 months after randomisation, 6 months prior to an event, or those obtained 6 months after randomisation and 6 months prior to an event were confirmatory.

**Table 5:** Hazard ratios associated with level and visit-to-visit variability of systolic blood pressure during follow-up with additional adjustment for proceeding to combination therapy as time-dependent variable.

		Mortality		Fatal plus non–fatal events		
Predictor variable (SD)	Model	Total	Cardiovascular	Cardiovascular	Stroke	Cardiac
All patients (*n* = 4695)						
SBP (∼13.6 mm Hg)	…	1.38 (1.20–1.58)§	1.69 (1.40–2.05)§	1.55 (1.37–1.76)§	1.65 (1.35–2.03)§	1.53 (1.31–1.79)§
	VIM	1.38 (1.20–1.58)§	1.70 (1.40–2.06)§	1.55 (1.37–1.75)§	1.65 (1.35–2.03)§	1.52 (1.30–1.78)§
VIM (∼5.5 units)	…	0.94 (0.81–1.09)	0.81 (0.66–1.01)	0.90 (0.79–1.03)	1.03 (0.84–1.28)	0.83 (0.70–0.99)*
	SBP	0.95 (0.82–1.10)	0.81 (0.66–1.01)	0.92 (0.80–1.05)	1.03 (0.83–1.27)	0.86 (0.72–1.02)
Placebo (*n* = 2297)						
SBP (∼13.5 mm Hg)	…	1.29 (1.08–1.53)†	1.66 (1.28–2.15)‡	1.48 (1.25–1.75)§	1.74 (1.29–2.35)‡	1.44 (1.17–1.77)‡
	VIM	1.28 (1.07–1.52)†	1.64 (1.27–2.13)‡	1.47 (1.24–1.74)§	1.74 (1.29–2.35)‡	1.43 (1.16–1.76)‡
VIM (∼5.6 units)	…	0.89 (0.72–1.10)	0.75 (0.56–1.01)	0.86 (0.71–1.03)	0.96 (0.72–1.28)	0.80 (0.63–1.02)
	SBP	0.91 (0.74–1.13)	0.77 (0.57–1.04)	0.88 (0.73–1.06)	0.97 (0.72–1.30)	0.83 (0.65–1.05)
Active treatment (*n* = 2398)						
SBP (∼12.6 mm Hg)	…	1.51 (1.19–1.91)‡	1.82 (1.30–2.54)‡	1.76 (1.43–2.17)§	1.73 (1.25–2.41)†	1.71 (1.29–2.26)‡
	VIM	1.51 (1.19–1.92)‡	1.85 (1.32–2.59)‡	1.77 (1.44–2.19)§	1.77 (1.26–2.49)‡	1.70 (1.29–2.25)‡
VIM (∼5.2 units)	…	0.96 (0.76–1.21)	0.85 (0.60–1.20)	0.90 (0.72–1.12)	1.01 (0.72–1.42)	0.86 (0.64–1.16)
	SBP	0.95 (0.76–1.20)	0.80 (0.56–1.15)	0.88 (0.70–1.10)	0.90 (0.62–1.30)	0.88 (0.65–1.19)

Level (SBP) and visit-to-visit variability independent of the mean (VIM) were calculated from all blood pressure readings available from randomisation until the occurrence of an endpoint or until the end of follow-up in patients without an event. Hazard ratios express the risk associated with a 1-SD increase in the predictor variable and were stratified for centre and adjusted for randomisation group (all patients), sex, age, body mass index, heart rate, cholesterol, smoking and drinking, history of cardiovascular disease or diabetes, and combination therapy as time-dependent covariable. Model indicates which systolic index was entered into the models in addition to the studied predictor. SDs are approximate, because the number of blood pressure readings available for analysis differed slightly depending on the timing of endpoints. An ellipsis signifies that in addition to the studied predictor no additional systolic index was entered.

Significance of the estimates:

*P≤0.05;

† *P*≤0.01;

‡ *P*≤0.001;

§ p<0.0001.

**Table 6:** Hazard ratios associated with level and within-visit variability of systolic blood pressure during follow-up with additional adjustment for proceeding to combination therapy as time-dependent variable.

		Mortality		Fatal plus non–fatal events		
Predictor variable (SD)	Model	Total	Cardiovascular	Cardiovascular	Stroke	Cardiac
All patients (*n* = 4695)						
SBP (∼13.6 mm Hg)	…	1.38 (1.20–1.58)§	1.69 (1.40–2.05)§	1.55 (1.37–1.76)§	1.65 (1.35–2.03)§	1.53 (1.31–1.79)§
	WVV	1.38 (1.21–1.59)§	1.70 (1.41–2.06)§	1.56 (1.37–1.76)§	1.65 (1.35–2.03)§	1.53 (1.31–1.80)§
WVV (∼2.3 mm Hg)	…	0.93 (0.76–1.15)	0.90 (0.68–1.20)	1.02 (0.86–1.21)	1.10 (0.84–1.45)	1.00 (0.80–1.25)
	SBP	0.90 (0.73–1.11)	0.86 (0.65–1.14)	0.96 (0.81–1.14)	1.03 (0.78–1.36)	0.95 (0.76–1.19)
Placebo (*n* = 2297)						
SBP (∼13.5 mm Hg)	…	1.29 (1.08–1.53)†	1.66 (1.28–2.15)‡	1.48 (1.25–1.75)§	1.74 (1.29–2.35)‡	1.44 (1.17–1.77)‡
	WVV	1.29 (1.08–1.54)†	1.67 (1.29–2.17)‡	1.48 (1.25–1.76)§	1.72 (1.27–2.32)‡	1.45 (1.18–1.79)‡
WVV (∼2.4 mm Hg)	…	0.99 (0.73–1.36)	0.90 (0.59–1.36)	1.02 (0.79–1.32)	1.27 (0.86–1.88)	0.88 (0.61–1.26)
	SBP	0.96 (0.71–1.30)	0.86 (0.58–1.28)	0.96 (0.75–1.24)	1.17 (0.79–1.72)	0.85 (0.60–1.20)
Active treatment (*n* = 2398)						
SBP (∼12.6 mm Hg)	…	1.51 (1.19–1.91)‡	1.82 (1.30–2.54)‡	1.76 (1.43–2.17)§	1.73 (1.25–2.41)†	1.71 (1.29–2.26)‡
	WVV	1.59 (1.24–2.03)‡	1.92 (1.36–2.72)‡	1.78 (1.44–2.20)§	1.73 (1.25–2.41)†	1.70 (1.28–2.26)‡
WVV (∼2.3 mm Hg)	…	0.78 (0.56–1.09)	0.82 (0.52–1.28)	0.96 (0.73–1.26)	0.90 (0.56–1.46)	1.10 (0.79–1.53)
	SBP	0.70 (0.50–0.99)*	0.69 (0.41–1.14)	0.89 (0.68–1.18)	0.91 (0.56–1.46)	1.02 (0.72–1.44)

Level (SBP) and within-visit variability (WVV) were calculated from all blood pressure readings available from randomisation until the occurrence of an endpoint or until the end of follow-up in patients without an event. Hazard ratios express the risk associated with a 1-SD increase in the predictor variable and were stratified for centre and adjusted for randomisation group (all patients), sex, age, body mass index, heart rate, cholesterol, smoking and drinking, history of cardiovascular disease or diabetes, and combination therapy as time-dependent covariable. Model indicates which systolic index was entered into the models in addition to the studied predictor. SDs are approximate, because the number of blood pressure readings available for analysis differed slightly depending on the timing of endpoints. An ellipsis signifies that in addition to the studied predictor no additional systolic index was entered.

Significance of the estimates:

*P≤0.05;

† *P*≤0.01;

‡ *P*≤0.001;

§ p<0.0001.

**Table 7:** Sensitivity analyses of the predictive value of visit-to-visit variability of systolic blood pressure with exclusion of varying follow-up periods.

		Mortality		Fatal plus non–fatal events		
Predictor variable (SD)	Model	Total	Cardiovascular	Cardiovascular	Stroke	Cardiac
Six months after randomisation (*n*≥2974)						
SBP (∼13.3 mm Hg)	…	1.11 (0.95–1.31)	1.40 (1.12–1.76)†	1.28 (1.09–1.50)†	1.33 (1.04–1.71)*	1.32 (1.09–1.62)†
	VIM	1.12 (0.95–1.32)	1.40 (1.11–1.76)†	1.28 (1.09–1.50)†	1.35 (1.05–1.74)*	1.31 (1.07–1.61)†
VIM (∼5.2 units)	…	0.99 (0.83–1.18)	0.90 (0.69–1.17)	0.94 (0.79–1.13)	1.07 (0.82–1.40)	0.88 (0.70–1.11)
	SBP	1.02 (0.85–1.22)	0.94 (0.72–1.23)	0.99 (0.83–1.19)	1.12 (0.85–1.48)	0.94 (0.75–1.19)
One year after randomisation (*n*≥2451)						
SBP (∼13.6 mm Hg)	…	1.01 (0.85–1.21)	1.23 (0.96–1.58)	1.15 (0.96–1.38)	1.37 (1.05–1.80)*	1.14 (0.91–1.44)
	VIM	1.00 (0.83–1.20)	1.20 (0.93–1.56)	1.12 (0.93–1.35)	1.34 (1.02–1.76)*	1.12 (0.88–1.42)
VIM (∼5.3 units)	…	0.93 (0.76–1.14)	0.83 (0.62–1.12)	0.85 (0.70–1.04)	0.81 (0.59–1.11)	0.89 (0.69–1.14)
	SBP	0.93 (0.76–1.14)	0.87 (0.64–1.18)	0.88 (0.72–1.08)	0.86 (0.62–1.20)	0.92 (0.71–1.19)
Six months before an event (*n*≥3474)						
SBP (∼13.0 mm Hg)	…	1.23 (1.05–1.45)*	1.55 (1.23–1.96)‡	1.29 (1.10–1.52)†	1.42 (1.10–1.83)†	1.26 (1.03–1.55)*
	VIM	1.23 (1.04–1.44)*	1.55 (1.22–1.95)‡	1.29 (1.10–1.52)†	1.44 (1.11–1.86)†	1.23 (1.01–1.52)*
VIM (∼5.2 units)	…	0.94 (0.79–1.12)	0.88 (0.67–1.17)	0.93 (0.77–1.12)	1.08 (0.82–1.42)	0.81 (0.64–1.04)
	SBP	0.98 (0.81–1.17)	0.94 (0.70–1.25)	0.98 (0.81–1.18)	1.13 (0.85–1.50)	0.85 (0.67–1.09)
Six months before an endpoint and six months after randomisation (*n*≥2941)						
SBP(∼13.3 mm Hg)	…	1.18 (0.99–1.40)	1.46 (1.15–1.86)†	1.23 (1.03–1.46)*	1.41 (1.08–1.84)*	1.20 (0.97–1.50)
	VIM	1.17 (0.98–1.39)	1.43 (1.12–1.83)†	1.20 (1.01–1.43)*	1.39 (1.06–1.81)*	1.17 (0.93–1.46)
VIM (∼5.2 mm Hg)	…	0.91 (0.76–1.10)	0.78 (0.58–1.05)	0.82 (0.67–1.01)	0.84 (0.61–1.15)	0.83 (0.65–1.07)
	SBP	0.95 (0.78–1.15)	0.83 (0.61–1.12)	0.86 (0.70–1.05)	0.89 (0.64–1.23)	0.87 (0.67–1.13)

SBP and VIM indicate level and variability independent of the mean of systolic blood pressure. Hazard ratios express the risk associated with a 1-SD increase in the predictor variable and were stratified for centre and adjusted for randomisation group, sex, age, body mass index, heart rate, cholesterol, smoking and drinking, and history of cardiovascular disease or diabetes. Model indicates which systolic index was entered into the models in addition to the studied predictor. SDs are approximate, because the number of patients and blood pressure readings available for analysis differed slightly depending on the timing of endpoints. An ellipsis signifies that in addition to the studied predictor no additional systolic index was entered.

Significance of the estimates:

*P≤0.05;

† *P*≤0.01;

‡ *P*≤0.001.

**Table 8:** Sensitivity analyses of the predictive value of within-visit variability of systolic blood pressure with exclusion of varying follow-up periods.

		Mortality		Fatal plus non–fatal events		
Predictor variable (SD)	Model	Total	Cardiovascular	Cardiovascular	Stroke	Cardiac
Six months after randomisation (*n*≥2974)						
SBP (∼13.3 mm Hg)	…	1.11 (0.95–1.31)	1.40 (1.12–1.76)†	1.28 (1.09–1.50)†	1.33 (1.04–1.71)*	1.32 (1.09–1.62)†
	WVV	1.12 (0.95–1.32)	1.41 (1.13–1.77)†	1.28 (1.09–1.50)†	1.31 (1.02–1.69)*	1.32 (1.08–1.62)†
WVV (∼2.2 mm Hg)	…	0.96 (0.74–1.24)	0.97 (0.66–1.42)	1.06 (0.85–1.33)	1.23 (0.93–1.64)	1.08 (0.79–1.48)
	SBP	0.94 (0.73–1.21)	0.90 (0.61–1.33)	1.02 (0.81–1.29)	1.19 (0.89–1.60)	1.03 (0.75–1.41)
One year after randomisation (*n*≥2451)						
SBP (∼13.6 mm Hg)	…	1.01 (0.85–1.21)	1.23 (0.96–1.58)	1.15 (0.96–1.38)	1.37 (1.05–1.80)*	1.14 (0.91–1.44)
	WVV	1.00 (0.84–1.20)	1.22 (0.95–1.58)	1.15 (0.96–1.37)	1.36 (1.03–1.78)*	1.13 (0.90–1.43)
WVV (∼2.3 mm Hg)	…	1.12 (0.83–1.51)	1.16 (0.75–1.79)	1.09 (0.83–1.43)	1.25 (0.87–1.78)	1.15 (0.83–1.60)
	SBP	1.12 (0.82–1.52)	1.11 (0.71–1.72)	1.06 (0.81–1.40)	1.19 (0.83–1.71)	1.13 (0.81–1.58)
Six months before an event (*n*≥3474)						
SBP (∼13.0 mm Hg)	…	1.23 (1.05–1.45)*	1.55 (1.23–1.96)‡	1.29 (1.10–1.52)†	1.42 (1.10–1.83)†	1.26 (1.03–1.55)*
	WVV	1.24 (1.06–1.46)†	1.57 (1.24–1.98)‡	1.30 (1.10–1.52)†	1.41 (1.09–1.82)†	1.27 (1.04–1.56)*
WVV (∼2.3 mm Hg)	…	0.75 (0.57–0.99)*	0.68 (0.44–1.06)	1.02 (0.80–1.31)	1.10 (0.76–1.60)	0.92 (0.66–1.30)
	SBP	0.74 (0.57–0.98)*	0.66 (0.43–1.03)	0.98 (0.76–1.25)	1.05 (0.72–1.51)	0.89 (0.63–1.24)
Six months before an endpoint and six months after randomisation (*n*≥2941)						
SBP (∼13.3 mm Hg)	…	1.18 (0.99–1.40)	1.46 (1.15–1.86)†	1.23 (1.03–1.46)*	1.41 (1.08–1.84)*	1.20 (0.97–1.50)
	WVV	1.19 (1.01–1.41)*	1.48 (1.16–1.88)†	1.24 (1.04–1.47)*	1.40 (1.07–1.83)*	1.21 (0.97–1.50)
WVV (∼2.2 mm Hg)	…	0.80 (0.60–1.08)	0.84 (0.54–1.29)	0.98 (0.75–1.27)	1.18 (0.84–1.67)	0.96 (0.67–1.37)
	SBP	0.79 (0.59–1.05)	0.79 (0.51–1.21)	0.94 (0.72–1.23)	1.12 (0.78–1.60)	0.92 (0.64–1.32)

SBP and WVV indicate level and within-visit variability of systolic blood pressure. Hazard ratios express the risk associated with a 1-SD increase in the predictor variable and were stratified for centre and adjusted for randomisation group, sex, age, body mass index, heart rate, cholesterol, smoking and drinking, and history of cardiovascular disease or diabetes. Model indicates which systolic index was entered into the models in addition to the studied predictor. SDs are approximate, because the number of patients and blood pressure readings available for analysis differed slightly depending on the timing of endpoints. An ellipsis signifies that in addition to the studied predictor no additional systolic index was entered.

Significance of the estimates:

*P≤0.05;

† *P*≤0.01;

‡ *P*≤0.001.

## Discussion

The novelty of our current study lies in the randomised double-blind placebo-controlled assessment of the risk associated with blood pressure variability and in accounting for blood pressure level while assessing blood pressure variability as cardiovascular risk factor. The key finding was that antihypertensive treatment lowered blood pressure and the incidence of cardiovascular, cerebrovascular and cardiac complications, but compared to placebo it did not affect visit-to-visit or within-visit blood pressure variability. In our current analysis, only blood pressure level, not variability, can therefore explain the benefit conferred by antihypertensive treatment. Kaplan-Meier survival estimates and fully adjusted Cox models confirmed that blood pressure level, but not visit-to-visit or within-visit variability predicted outcome. The findings were consistent in the placebo and active-treatment groups analysed separately.

Our manuscript differs substantially from previous publications on the role of blood pressure variability as cardiovascular prognosticator. First, our analyses followed the lines of randomisation in a double-blind placebo-controlled trial [15]–[17]. Without the need of complex statistical modelling, figure 2 clearly demonstrates that only level can explain the difference in outcomes between the two treatment groups. Previous reports on visit-to-visit variability analysed clinical trial data as cohort studies [2], [4], [22], [23] with [2], [4], [22] – or even without [22], [23] – a design variable in multivariable models coding for randomisation group. Second, advocates of the role of blood pressure variability as cardiovascular risk factor support the idea that stroke is the outcome most likely associated with variability over and beyond level of blood pressure [1], [3], [4]. Syst-Eur was powered to demonstrate a between-group difference in the incidence of stroke, the primary endpoint of the trial [15]–[17]. The wide-spread introduction of stroke and coronary care units and the increasing availability of invasive intravascular procedures and thrombolysis dramatically reduced the case-fatality rate of most cardiovascular complications of hypertension. Not accounting for non-fatal events limits the generalizability of several reports [9], [13]. Third, our multivariable-adjusted Cox models analysed variability as a continuous variable, avoiding the problem of categorisation [1], [2], [9], [23]. Fourth, cardiovascular endpoints are often preceded by changes in blood pressure related to the subsequent complication. Such blood pressure changes should not be considered to predict a complication, but are actually part of the imminent event [24]. Sensitivity analyses of VIM (table 7) and WVV (table 8), from which we excluded blood pressure measurements obtained within the first 6 or 12 months after randomisation, 6 months prior to an event, or those obtained 6 months after randomisation and 6 months before an event were confirmatory. Finally, we showed that our main measure of visit-to-visit blood pressure variability was independent of the mean.

Factors explaining the difference with previous studies [1]–[4], [8]–[14] are the analysis of the data according to the random allocation of patients, the double blind design, the multivariable adjustment including blood pressure level, the analysis of blood pressure variability as a continuous rather than as a categorical variable, and the implementation in Syst-Eur of a comprehensive quality control programme of the blood pressure measurements [19]. We do not believe that selection of patients with isolated systolic hypertension, age, or use of nitrendipine as first-line antihypertensive drug explain why in our study blood pressure variability did not predict outcome. Indeed, the average blood pressure at entry into ASCOT-BPLA [25] was 164.0/94.7 (SD, 18.0/10.4) mm Hg. The SD indicates that a substantial number of ASCOT patients must have had isolated SBP, as defined in our current study (160–219/<95 mm Hg). In ASCOT-BPLA [25], 63% of randomised patients were older than 60 years. In ASCOT-BPLA [1], systolic VIM and WVV averaged 13.1 (5.2) units and 5.91 (0.02) mm Hg on atenolol and 11.1 (4.5) and 5.42 (0.02) mm Hg on amlodipine with between-group differences of 1.99 units (CI, 1.93 to 2.05) and 0.49 mm Hg (CI, 0.44 to 0.54), respectively. In Syst-Eur, systolic VIM and WVV averaged 10.2 (5.7) units and 3.34 (2.35) mm Hg on placebo and 10.2 (5.2) units and 3.29 (2.24) mm Hg on active treatment with between-group differences of 0.01 units (CI, −0.30 to 0.33) and 0.05 mm Hg (CI, −0.08 to 0.18), respectively. These measures of variability were lower in Syst-Eur than in ASCOT-BPLA [1]. However, use of nitrendipine as the first-line antihypertensive agent in Syst-Eur cannot explain the discordance between our findings and those reported for ASCOT-BPLA, because in Syst-Eur VIM and WVV were similar on active and placebo nitrendipine. In our study, the indexes of variability increased after randomisation. Patients could be randomised if during the single-blind placebo run-in period SBP ranged from 160 mm Hg to 219 mm Hg and diastolic blood pressure was below 95 mm Hg. These blood pressure criteria were the averages of six readings, two at three consecutive run-in visits approximately 1 month apart. After randomisation, these constraints on SBP level disappeared, explaining the increase in variability.

The current study must be interpreted within the context of some potential limitations. First, Syst-Eur involved older patients with isolated systolic hypertension, of whom only one third had a history of previous cardiovascular complications. Our current observations might not be generalizable to younger individuals, patients with more serious cardiovascular disease, in whom blood pressure regulation is often compromised, or patients with predominant diastolic hypertension. Second, the Syst-Eur trial was terminated early at the second interim analysis because of a significant reduction of stroke, while recruitment was still on-going [15]. This explains why the number of analysable patients dropped from 4695 to around 2500 in some sensitivity analyses, from which blood pressure readings obtained during specified follow-up periods were excluded. In spite of the loss of power and the drop in significance levels, sensitivity analyses were confirmatory. Finally, nitrendipine was the first-line drug in the active-treatment group, and enalapril and hydrochlorothiazide were used as add-on medications. However, at 2 years, close to 60% of patients allocated active treatment were still on monotherapy with nitrendipine. Findings in patients remaining on monotherapy and in those proceeding to combination therapy and models that included a time-dependent covariable coding for proceeding to combination therapy produced consistent results. These observations confirm a meta-analysis showing that drug class effects on blood pressure variability persist when medications were used in combination [26].

In conclusion, in Syst-Eur, higher blood pressure level, but not higher variability, predicted risk. Blood pressure level is a reversible risk factor, overriding all other modifiable risk factors. The suggestion to consider blood pressure variability as an additional risk indicator and to reduce it by drugs [1], [4] detracts from what really matters in the management of hypertension, that is controlling the blood pressure level. In keeping with the recent European guideline [7], blood pressure variability remains a research instrument and requires further evaluation before it can be meaningfully applied in clinical practice.

## Supporting Information

Protocol S1 **Ethics approval, original protocol, and trial registration.**
(PDF)

Checklist S1 **CONSORT checklist.**
(DOC)
