# Supplementary material for: Randomised Double-Blind Comparison of Placebo and Active Drugs for Effects on Risks Associated with Blood Pressure Variability in the Systolic Hypertension in Europe Trial
Source: PLoS One. 2014 Aug 4;9(8):e103169. doi: 10.1371/journal.pone.0103169 (PMC4121168; doi:10.1371/journal.pone.0103169)
Supplement: Protocol S1 — Ethics approval, original protocol, and trial registration. (PDF) [file pone.0103169.s001.pdf]

BIJLAGE 6

Laboratorium voor Experimentele Geneeskunde en Endocrinologie
(LEGENDO)
Onderwijs en Navorsing

Gasthuisberg
B 3000 LEUVEN - Belgium
Tel. (016) 21 59 70

Katholieke Universiteit Leuven

Aan Prof. Dr. A. AMERY
Hematologie

O. Ref.: 88/232WH(bm)

Leuven, 24 maart 1988.

Betreft: Tweede europese studie over hypertensie boven de 60 jaar

Geachte Collega,

De Ethische Commissie van de Faculteit Geneeskunde heeft dit protocol onderzocht op haar vergadering van 23 maart 1988 en met U besproken. De leden van de Commissie menen dat er geen ethische bezwaren zijn tegen deze studie, zoals beschreven in het protocol.

Met de meeste hoogachting.

Prof. Dr. P. DE SCHEPPER
Voorzitter Ethische Commissie

Prof. Dr. W. HEYNS
Secretaris

GUIDELINES FOR PATIENT CHARTS

OF THE SYST-EUR TRIAL

CONTENTS

I. Reports required during the trial.

II. Logbook.

III. Rules for completion of the patients forms during the study.

IV. Electrocardiogram.

V. What to do if...

VI. Errata.

VII. Contact persons at the CO.

I. REPORTS

The following charts will be used during the trial :

I.1 Logbook : lists of all patients eligible for the placebo run-in phase of the study.

I.2 Forms for the placebo run-in period of the trial

1. Short report during placebo run-in period : to be completed at the first and third out-patient visit on placebo run-in medication, i.e. after one and three months of placebo run-in treatment.

2. Extensive report during placebo run-in period : to be completed at the second out-patient visit on placebo run-in medication.

2bis. Addendum to the extensive report during placebo run-in period : to be completed at the second visit on placebo run-in medication.

At the SYST-EUR investigator's meeting of 4 November 1988, it was decided to collect more precise information on previous antihypertensive treatment at the occasion of the second run-in visit. After the pilot running of the trial, new forms will be printed. This addendum (2bis) concerning previous antihypertensive treatment will then be added to the extensive report form of the placebo run-in period.

3. Checklist : to be completed at the third visit on placebo run-in medication.

I.3 Forms for the double-blind period of the trial

1. Short report during double-blind period : to be completed at quarterly intervals.

2. Extensive report during double-blind period : to be completed at yearly intervals.

3. Report at the end of the double-blind period : to be completed when double-blind treatment is stopped.

I.4 Forms for supervised open follow-up

When the patient continues to attend the clinic after stopping double-blind treatment, the following forms will be used :

1. Short report during supervised open follow-up : to be completed at quarterly intervals.

2. Extensive report during supervised open follow-up : to be completed at yearly intervals.

3. Report at the end of the supervised open follow-up : to be completed when :

   i) A patient dies during supervised open follow-up

   ii) Regular follow-up is not longer possible

I.5 Forms for non-supervised open follow-up

Annual report during non-supervised open follow-up : to be completed at yearly intervals when :

i) Double-blind treatment is stopped and no supervised open follow-up is possible

ii) Supervised open follow-up is stopped

The patient forms for the different periods of the trial are identified by colour codes printed in various ways (see table).

Form	Logbook Checklist	placebo run-in	double-blind period	supervised open follow-up	non-supervised open follow-up
	white	pink	blue	green	yellow
Short	-	band (top)	band (top)	band (top)	-
Extensive	-	whole page	whole page	whole page	whole page
End	-	-	blocks (top)	blocks (top)	-

II. LOGBOOK

The aim of keeping a logbook is to have an idea on the total number of patients, who are considered by the investigator for entry into the placebo run-in phase of the study. This information may be important in evaluating the representativeness of the patients who will finally be randomised.
Thus the following patients should be entered into the logbook :

1. All patients who are actually treated with placebo run-in medication.

2. All patients, who apparently comply with the entry criteria of the trial and who the local investigator at a certain moment has considered for entry, but who for various reasons were not started on placebo run-in treatment, e.g. patient does not give informed consent; patient lives too far; patient appears at second look to have other medical problems, which constitute an exclusion criterion; etc...

Information on the following items is requested :

Name-First Name : Name or initials of the patient. The surname (initial of the surname) should be written before the first name (initial of the first name).

Gender : self-explanatory.

Date of birth : self-explanatory.

Date of visit : self-explanatory.

Blood pressure : systolic and diastolic blood pressure, measured when the patient was considered for entry into the run-in phase of the study. The sitting pressure is preferred, but if not available, also the supine or standing pressure may be recorded. If available, the untreated pressure should be given; otherwise the pressure while the patient was still on antihypertensive medication may be recorded in the logbook.

Treatment at time of BP-recording : At the SYST-EUR investigator's meeting of 4 November 1988, it was decided to record only whether or not the patient was previously treated with antihypertensive drugs. Please indicate in the logbook by yes or no the treatment status of the patients who are being considered for entry.

Admitted in run-in period of trial : Reply by yes or no. If the patient was not entered in the run-in period of the trial, please specify the reason why not.

Admitted in double-blind period of trial : Indicate by yes or no whether or not the patient was admitted in the double-blind period. If the patient was not admitted, please specify the reason why not.

Each logbook carries the centre number and logbook number. Centre number and logbook number will be provided by the Coordinating Office. A centre may use several logbooks at the

same time, e.g. when patients are being recruited in different departments. However every six months new logbook(s) should be started. The previous logbook(s) should then be fully completed and mailed back to the Coordinating Office. A copy of each page can be retained for local use.

The cover of the logbook has a flap which can be placed between pages to prevent that the handwriting presses through to several pages.

III. RULES FOR COMPLETION OF THE PATIENT FORMS DURING THE STUDY

III.1 General rules

(1) Charts will be completed and sent to the Coordinating Office as soon as possible after each patient visit, e.g. during the placebo run-in period at monthly intervals, namely after the first, second and third placebo run-in visit. Before sending the charts to the Coordinating Office, the local investigator will check whether the information on the patient charts is accurate and complete. Each patient form has a top copy to be mailed back to the Coordinating Office and a carbon copy which can be removed for local use.

(2) The big open boxes should be completed by the local investigator, while the small shaded boxes are reserved for the Coordinating Office. Asterisks indicate information which is optional.

III.2 Items to be completed by the Coordinating Office

The following items will be filled in by the Coordinating Office :

- ICD-codes
- Drug-codes
- Codes for comments
- Minnesota codes for special findings on the electrocardiogram.

III.3 Reference guide

Entries in alphabetical order :

- Activities of daily living : These 6 questions need to be answered either by "Ø" if the answer is negative or by "1" if the answer is positive. The fourth question will be answered by "1" (yes) if all conditions are fulfilled, i.e. if a patient can move in and out bed and in and out chair without help. Thus, if the patient needs help for one of the several functions in this fourth question, the answer to this question is "Ø" (negative). The scores for the six questions should be summed to give the total ADL-score.

- Any other drugs : see "drug codes".

- Blood pressure measurements : (see appendix III of the protocol) At each visit two measurements should be made in each position i.e. in the supine position after two minutes rest, in the sitting position after 5 minutes rest and in the standing position (after two minutes). At least one minute should elapse between measurements. If a cuff with large size is used, this should be noted in the comment section on the patient form; in each patient the same cuff should be employed throughout the study.

- Blood tests : Results of these tests should be reported in SI-units or the units specified on the form. For conversion to SI-units, the following factors can be used :

Table : conversion of conventional units to SI-units and vice versa

- Haemoglobin	:	(g/dl) x 0,62 = (mmol/l)<br>(mmol/l) x 1,61 = (g/dl)
- Creatinine	:	(mg/dl) x 88,4 = (µmol/l)<br>(µmol/l) x 0,01131 = (mg/dl)
- Uric acid	:	(mg/dl) x 0,059 = (mmol/l)<br>(mmol/l) x 16,81 = (mg/dl)
- Glucose	:	(mg/dl) x 0,056 = (mmol/l)<br>(mmol/l) x 18,02 = (mg/dl)
- Cholesterol	:	(mg/dl) x 0,026 = (mmol/l)<br>(mmol/l) x 38,66 = (mg/dl)
- Triglycerides<br>(MM = 875)	:	(mg/dl) x 0,011 = (mmol/l)<br>(mmol/l) x 87,5 = (mg/dl)<br>(mg/dl) x 0,01 = (g/l)<br>(g/l) x 100 = (mg/dl)
- Na	:	(meq/l) x 1 = (mmol/l)
- K	:	(meq/l) x 1 = (mmol/l)

ref. : see N. Engl. J. Med., 1980, Vol. 302, n° 1, 37-48.

- Checklist : The checklist will be sent to the Coordinating Office together with the report of the third visit during the placebo run-in period.
  Each entry on the checklist should be completed. At the Coordinating Office the patient will be considered for randomisation if informed consent has been obtained and if yes is entered for all inclusion criteria and no for all exclusion criteria.

- Diagnosis of hypertension : Possible causes of hypertension are classified in appendix IX of the protocol.

- Diseases and findings : All diseases and findings which were mentioned in the last report form and which are still active at the moment when the patient visits again should be specified under "Diseases and findings still active". Diseases and findings which were not mentioned on the last report form should be specified under "New diseases and findings".

- Drug codes : The generic name of the drugs will be given. If a drug contains several compounds, each compound has to be specified as a separate entry on the form. Coding of the drugs will be done at the Coordinating Office.

- Identification & registration : For reasons of confidentiality it was agreed that the patient can be identified as follows :

  1) During the placebo run-in period by initials and date of birth.

  2) After randomisation by initials, date of birth and patient identification number.

  The initial of the surname should be written before the initial of the first name, e.g. John Smith should be given as S.J.

- Information on treatment : The number of tablets per day should be given in decimal values.

  e.g. [0].[5] = half a tablet per day

  [1].[0] = one tablet per day

  [1].[5] = one and half a tablet per day

- Laboratory tests : see "Blood tests" and "Urine tests".

- Logbook : The logbook number is the number of the logbook in which the patient is entered. This number is mentioned on the cover of the logbook. The row number is the number of the patient in the specified logbook. This number is printed left of the patient's name.

- Non-supervised open follow-up : When a patient does not continue to attend the clinic, the local investigator should complete a report form for non-supervised open follow-up at yearly intervals. The requested information should be obtained by writing, telephone, or visits, either directly from the patient or via family members, General Practioners, other hospitals, population registers or via Offices for Vital Statistics.

- Physical examination :

  For blood pressure measurements see entry on page III.2.
  Weight and height must be given in decimal values, e.g.
  [ ] [5] [0] . [5] for a weight of fifty and half a kilogram.

- Reason : see "Report at the end of the double-blind period" or "Report at the end of supervised open follow-up".

- Report at the end of the double-blind period : This form should be completed for each randomised patient when double-blind treatment is stopped. If a patient dies, or if a patient has not been examined, only page one and two of the form has to be completed. For all other patients, page one through five must be filled in.

  Each entry in the section entitled "Reason" should be completed. However, only one possibility for stopping double-blind treatment can be answered by yes.

  Before completing this report form, the local investigator will carefully read chapter XIII of the main protocol. Definitions of cardiovascular events are given in appendix IV of the protocol.

  If an autopsy is performed, a copy of the protocol should be sent to the Coordinating Office. Each protocol must be identified by the patient identification number , initials and date of birth.

- Report at the end of supervised open follow-up : This form should be completed in the same way as the report at the end of the double-blind period (see "Report at the end of the double-blind period").
  If a patient dies, or if a patient has not been examined only page one of the form will be completed. For all other patients, page one through four must be filled in.

- Serum samples : Serum samples should be stored preferably at -70 to -80°C. Guidelines for sending these serum samples to the Coordinating Office are currently being worked out. Serum and urine samples will be collected at yearly intervals.

- Smoking and drinking habits :

  Drinking habits : On the patient forms for placebo run-in period, the number of glasses alcohol per day should be given. On the patient forms for the double-blind and open follow-up period, the number of glasses of alcohol per week must be filled in. After the pilot trial, new patient forms will be printed. The question about alcohol consumption will be made identical on all the patient forms (alcohol consumption per week). If the patient consumes less than one glass of alcoholic beverages per week (day), the answer to the question whether or not the patient consumes alcoholic beverages now, is yes (code 1) and the quantity may be given as a fraction e.g. [0] [1] / [2] if one glass per 2 weeks (days) is consumed.

  Smoking habits : A patient is called an ex-smoker (code 1) when he (she) smoked in the past, but does not smoke anymore at the start of the placebo run-in period.
  If a patient consumes less than one cigarette, cigar or pipe per day, the answer to the question whether or not the patient is smoking now, is yes (code 2), but the quantity may be expressed as a fraction e.g. [0] [1] / [2] if one cigarette per 2 days is consumed.

- Supervised open follow-up : When a patient continues to attend the clinic after stopping double-blind medication, a short report for supervised open follow-up should be completed at quarterly intervals and an extensive report at yearly intervals.

- Urine tests : Urine samples will be collected at yearly intervals during the double-blind period and if possible, also at the end of the double-blind period. Each urine sample should be identified by date of urine sampling, patient identification number, initials and date of birth.
  Before sending, urine samples may be stored at -20 to -40°C. Practical guidelines for sending these urine samples to the central laboratory (Prof. A. De Schaepdryver, Heymans Institute of Pharmacology, University of Ghent, Belgium) are currently being worked out.

IV. ELECTROCARDIOGRAM

The ECG will be recorded as follows :

- A calibration mark should be recorded, in each channel. This calibration mark has to be adjusted to exactly 10 mm. Leads recorded with a reduced calibration should be preceded by a calibration mark of exactly 5 mm.
- The tracings will be recorded at a paper speed of 25 mm per second and for each lead, I, II, III, aVR, aVL, aVF, V_1 to V_6 , a strip of at least 5 seconds should be obtained.
- Baseline drift and noice should be avoided.
- Each ECG should be identified as follows :
  1) During placebo run-in period by date of recording, date of birth and initials of the patient.
  2) After randomisation by date of recording, patient identification number, initials and date of birth.

More detailed guidelines for ECG-recordings are given in appendix VIII of the protocol.

Calibration signal, R- and S-waves should be measured as described by Prineas et al. in the Minnesota Code Manual of Electrocardiographic Findings, 1982 :

- The calibration signal is measured from the top margin of the baseline to the top of the square wave (fig. 1).

- R-waves are measured to the nearest whole millimeter in the next to last complete normal beat in the appropriate leads. The R-wave amplitude is the vertical distance between the upper margin of the P-R baseline at the onset of the QRS complex and the peak of the R-wave, even if the R-wave is preceded by a Q-wave (fig. 2 and fig. 3).

- The maximum S-wave is measured in the same way as the maximum R-wave.

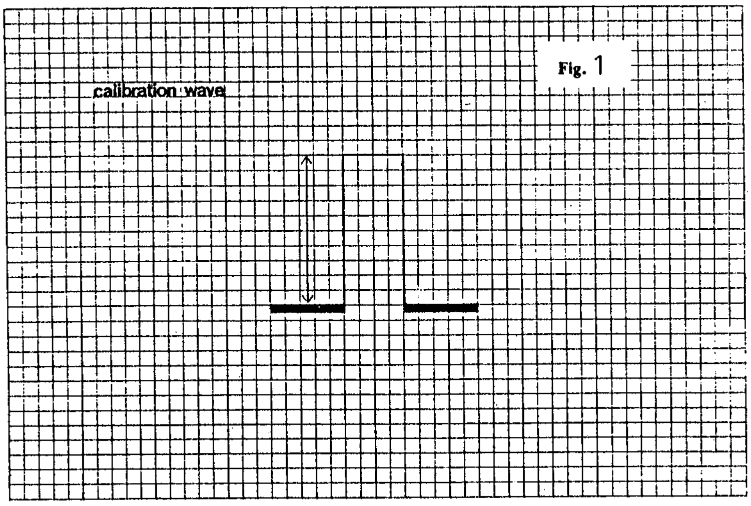

Fig. 1

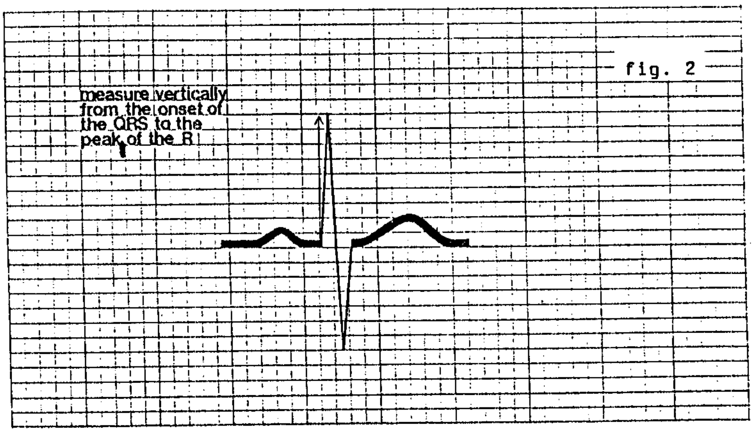

fig. 2

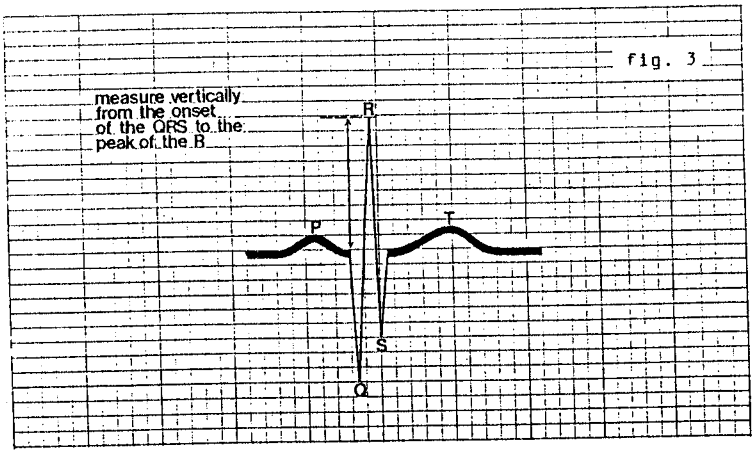
fig. 3
measure vertically from the onset of the QRS to the peak of the R
R
P
T
S
Q

V. WHAT TO DO IF...

V.1 Placebo run-in period is stopped

1. The patient has reached the end of the placebo run-in period, i.e. he (she) has been treated with placebo run-in medication for three months :
   The local investigator should send the checklist to the Coordinating Office together with the report form of the third run-in visit. At the Coordinating Office the patient will be considered for randomisation into the study.
   Double-blind treatment should be started as soon as possible after receipt of the double-blind medication (provided by the Regional Drug Dispatching Centre) and the patient identification number (provided by the Coordinating Office). In the mean time placebo run-in treatment should be continued. Thus, the answer to the question on the third report form "Does the patient continue run-in period", should be affirmative (code 1).

2. Placebo run-in period is stopped prematurely, i.e. before the patient has been treated with placebo run-in medication for three months :
   The reason why placebo run-in medication is stopped should be specified in the section entitled "Decisions and Comments".
   If the patient does not attend the clinic for the second or third placebo run-in visit, an extensive report form

should be completed for the missed second visit and a short report form for the missed third visit. In these instances, only the sections entitled "Identification & Registration" and "Decisions and Comments" need to be filled in.

V.2 Double-blind treatment is stopped

The local investigator will complete the report form for the end of the double-blind period (see II.3 : Report at the end of the double-blind period).
If the patient still attends the clinic after the double-blind period is terminated, he (she) enters the period of supervised open follow-up. Short report forms should be completed at quarterly intervals and an extensive report at yearly intervals.
If the patient is still alive, but does not continue to attend the clinic after stopping double-blind treatment, he (she) enters the period of non-supervised open follow-up. During non-supervised open follow-up a report will be completed at yearly intervals and mailed to the Coordinating Office. (see III.3 : Non-supervised open follow-up)

V.3 Supervised open follow-up is stopped

When a patient dies during supervised open follow-up or when regular follow-up is not longer possible, a report form for the end of supervised open follow-up should be filled in.

If the patient is still alive at the end of supervised open follow-up, he (she) enters the period of non-supervised open follow-up. In this instance, the report form for non-supervised open follow-up must be completed annually. (see III.3 : Non-supervised open follow-up)

VI. ERRATA

The following corrections should be made on the report forms for the placebo run-in period :

- Activities of daily living : The fourth question should be replaced by "Can the patient move in and out bed and chair alone ?" (see III.3 : Activities of daily living).

- Electrocardiogram : "Height of RV_1" must be replaced by "Height of SV_1".

- Information on treatment : The question "Interruption since last visit ?" should be replaced by "Dose reduction or interruption since last visit ?".

- Physical examination : To specify height and weight more accurately, a fourth box for the decimal part may be added.

- All open boxes for ICD-codes should be replaced by dotted boxes, to be filled in by the Coordinating Office.

VII. CONTACT PERSONS AT THE COORDINATING OFFICE

If you have any additional questions about the completion of the patient charts, you can contact one of the following persons :

1. Mrs. L. Thijs
   Hypertension Unit
   Research Building
   Campus Gasthuisberg
   Herestraat 49
   B-3000 LEUVEN
   Belgium

   Tel. : 32/16/21.57.67

   Fax Number : 32/16/21.59.91
   KUL-MED-RESEARCH

   Telex Number : 24484

   EARN Number : THIJS @ BLEKUL50

2. Mr. M. Laermans

   Tel. : 32/16/21.57.63

   Address, Telex Number, Fax Number : idem (Thijs)

   EARN Number : LAERMANS @ BLEKUL50

3. Dr. J. Staessen
   Internal Medicine-Cardiology
   University Hospital Gasthuisberg
   Herestraat 49
   B-3000 LEUVEN
   Belgium

   Tel. : 32/16/21.36.31

   Fax Number, Telex Number : idem (Thijs)

   EARN Number : STAESSEN @ BLEKUL50

4. Prof. A. Amery

   Address, Telex Number, Telephone Number, Fax Number :
   idem (Staessen)

   EARN Number : AMERY @ BLEKUL50

SYST-EUR

EUROPEAN TRIAL ON SYSTOLIC HYPERTENSION IN PATIENTS AGED 60 OR MORE

TABLE OF CONTENTS

PART I : MAIN PROTOCOL OF SYST-EUR

I.	Aim	P 2
II.	Rationale	P 2
III.	Objectives of the trial	P 3
IV.	Design of the trial	P 4
V.	Investigators and location	P 5
VI.	Sample size and timeframe	P 6
VII.	Single-blind placebo run-in period	P 7
VIII.	Patient definition	P 9
IX.	Stratification of patients	P 13
X.	Definition of admission into the study	P 15
XI.	Treatment during the double-blind period	P 16
XII.	Measurements during the study	P 21
XIII.	End of the double-blind part of the study in individual patients	P 30
XIV.	Statistical analyses and guidelines for stopping the trial	P 36

PART II : SIDE-PROJECTS IN SYST-EUR

I. Quality of life assessment by questionnaire (C.J. Bulpitt, A. Fletcher)

II. Twenty-four hour blood pressure monitoring in SYST-EUR (J. Staessen, G. Mancia)

III. Determination on stored serum samples (Prof. De Schaepdryver)

IV. Twenty-four hour ECG-recording in SYST-EUR (N...)

V. Echocardiography in SYST-EUR (R. Fagard)

VI. Study of multiple infarction dementia (F. Forette)

PART III : APPENDICES TO THE PROTOCOL OF SYST-EUR

I. Literature references to section II of the main protocol.

II. Sample size calculations

III. Guidelines for blood pressure measurements

IV. Definitions of cardiovascular events

V. Procedures for randomizing patients and supplying study medication

VI. Patient charts

VII. Activities of daily living (ADL-score)

VIII. ECG-recording in multicentre clinical trials

IX. Classification of hypertension

X. Trial termination

XI. Patient confidentiality

XII. Proposal for committees

XIII. Addresses

PART I

MAIN PROTOCOL OF

SYST-EUR

I. AIM

SYST-EUR aims to investigate the influence of antihypertensive drug therapy on mortality, morbidity and general well-being in patients aged 60 or more with systolic hypertension.

II. RATIONALE

1. Several studies (1, 2) have demonstrated that systolic blood pressure is a major risk factor for cardiovascular events, and that systolic pressure in the elderly is probably even more important than diastolic blood pressure.

2. It has conclusively been shown in certain groups of hypertensives (3), including elderly patients (4-7) that antihypertensive drug therapy reduces cardiovascular mortality and morbidity. However, presently there is no evidence assessing the possible benefit of antihypertensive drug therapy in patients with systolic hypertension.

References : see Appendix I.

III. OBJECTIVES OF THE TRIAL

The principal goals of SYST-EUR are to assess in elderly patients with systolic blood pressure elevation the influence of antihypertensive drug treatment on : (1) cardiovascular mortality; (2) cardiovascular morbidity; (3) and general well-being.

Several side-projects constitute an optional part of the trial, to be undertaken by selected centres only.

A. A detailed quality of life assessment by questionnaire (see part II, section I).

B. A study of the predictive value of 24-hour blood pressure recordings (see part II, section II).

C. A study of biochemical variables, such as serum lipids and other indicators of cardiovascular risk (see part II, section III).

D. A study on the influence of antihypertensive drug treatment on cardiac rhythm by 24-hour ECG-recordings (see part II, section IV).

E. An echocardiographic evaluation of the patients randomized into the main study : this side-project was not retained (see part II, section V).

F. A study of multiple infarction dementia (see part II, section VI).

IV. DESIGN OF THE TRIAL

The trial is designed in order to allow an intention-to-treat analysis. It comprises:

1. A single-blind run-in period on placebo.
2. A double-blind, randomized study of one control and one treatment group in each of four strata of patients (see section IX for stratum definition).

All patients will be followed until the end of the trial irrespective of their treatment status.

V. INVESTIGATORS AND LOCATION

In order to recruit a sufficient number of patients, SYST-EUR will be run as a multicentre trial. Several committees will be involved in the conduct of the trial (For proposal see Appendix IX).

The Coordinating Office is responsible for the day-to-day conduct of the trial : stratification and randomization procedures, data processing and statistical analyses. The Coordinating Office is located in Leuven, Belgium (Hypertension Unit, Gasthuisberg University Hospital, 49 Herestraat, B-3000 Leuven, Belgium, telephone 32-16-215767, telefax 32-16-215991 KUL MED RESEARCH). For protection of patient confidentiality see appendix XI.

VI. SAMPLE SIZE AND TIMEFRAME

Sample size calculations are summarized in Appendix II.

A total of 3,000 patients will be randomized into SYST-EUR. Collaborating centres will each randomize around 30 patients (or more) over a 2 year period.

A one year pilot trial will start on October 1, 1988. If no major alterations of the protocol are necessary the patients entered in the pilot trial will continue into the main study. The rest of the patients in the main trial will be recruited over 2 years (1990-1991) and all patients will be followed until 1996 (5 years or more). Data analysis and publication will require 2 years (1997-1998).

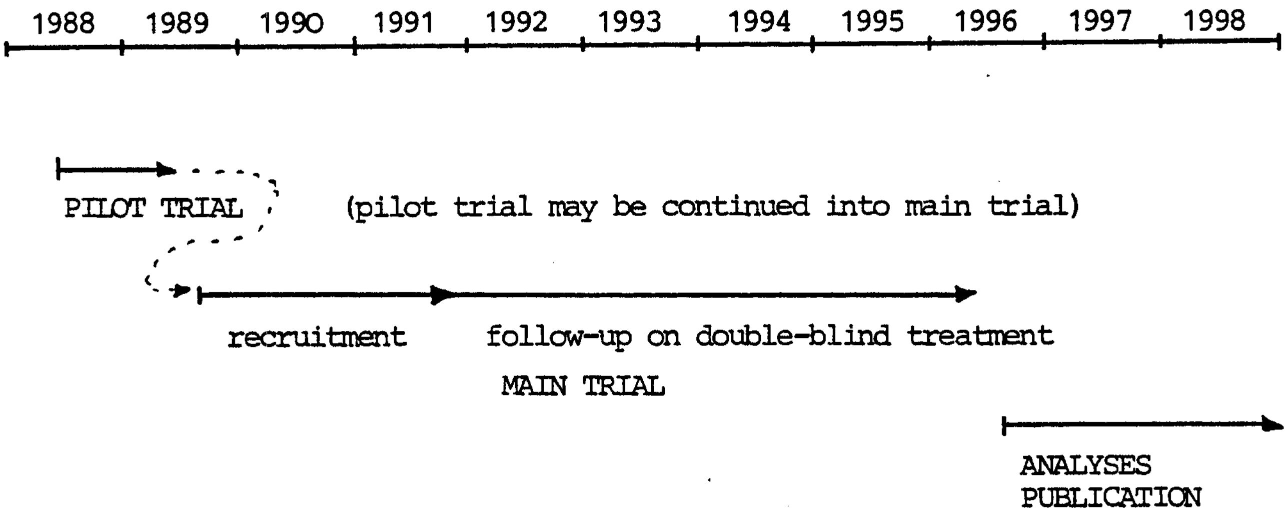

VII. SINGLE-BLIND PLACEBO RUN-IN PERIOD

Informed consent will be obtained before recruitment. Thereafter patients will be followed during a single-blind run-in period of three months. During the run-in period patients should be examined at least three times. They will receive for their hypertensive condition a placebo (half a tablet in the evening).

Newly diagnosed as well as patients with known systolic hypertension may be considered for recruitment into the trial. However, all drugs with hypotensive action which the patient may be receiving should be discontinued at the start of the run-in period. In some cases, depending on the drugs and clinical condition, this should be done progressively over a few days.

Compliance of the patients during the run-in period will be evaluated mainly on the basis of clinic attendance. Riboflavin containing tablets will not be used to check compliance.

Following each of the three exams of the placebo run-in period, a report with the individual data on each patient will be sent to the Coordinating Office, where the patient will be stratified according to the criteria defined below. Patients will then be given their study number. The latter will consist of 4 blocks of digits : (1) centre number =

3 digits; (2) one digit identifying the strata; (3) patient number = 3 digits; (4) two check digits (example : 321-2-984-53) [1].

A logbook will be provided by the Coordinating Office. This logbook should be kept in each centre and will consist of : (1) a list of patients fulfilling the admission criteria, but not admitted into the run-in period; (2) a list of patients admitted into the run-in period of the trial. These lists will contain for each patient the following items :

- patient's name, age and sex
- patient's blood pressure readings
- was the patient admitted in the run-in period and in the double-blind study ? if not : why ?

A copy of the logbook will be requested by the Coordinating Office at regular intervals.

(1) Check digits are given by the following formula: 3212984 - [int. (3212984/97)x97].

VIII. PATIENT DEFINITION

A. Inclusion criteria

All of the following criteria should be met :

1. Age of 60 years or more at randomization into the study without an upper age limit (age = time elapsed from brith date to date of randomization).

2. Blood pressure during the placebo run-in period : all of the following conditions must be met :
   - the average systolic pressure in the sitting position of the 3 visits combined : 160 through 219 mm Hg
   - the average diastolic pressure in the sitting position of the 3 visits combined : 94 mm Hg or less with no lower limit
   - the average systolic pressure in the standing position of the 3 visits combined : 140 mm Hg or more.

   At each of the three visits during the placebo run-in period two blood pressure readings will be obtained in each of the supine, sitting and standing positions. Thus, the average sitting blood pressure to be used for eligibility will be obtained from 6 readings (2 readings at 3 visits). The methods of blood pressure recording are described in Appendix III.

3. Informed consent should be obtained from each patient. Patients must be willing to cooperate and (according to the judgement of the local investigator) regular follow-up must be possible, e.g. patients who are planning to move

or who are alcoholics should not be included because it is likely that regular follow-up in such patients is impossible. The doctor who proposes a patient for randomization into the trial remains in charge for follow-up and treatment.

B. Exclusion criteria

None of the following conditions should be present :

1. certain causes of systolic blood pressure elevation :

a. specific cause of systolic blood pressure elevation, such as hyperthyroidism

b. conditions which will be corrected by surgery (or other procedures such as percutaneous dilatation of the renal artery), such as coarctation of the aorta, Cushing syndrome, Conn syndrome, renovascular hypertension, phaeochromocytoma, etc...

c. severe aortic insufficiency (grade III or IV) or AV-fistula. These conditions are excluded because they can lead to high systolic and low diastolic pressure.

2. certain complications of hypertension :

a. presence at time of recruitment :

- vascular retinopathy grade III or IV (i.e. papilloedema)
- overt congestive heart failure
- dissecting aneurysm
- severe renal failure defined as a serum creatinine of 180 µmol/L (2.0 mg/dl), or more.

b. history of :

- repeated severe nose bleeds due to hypertension, not controlled by local measures
- cerebral or subarachnoid hemorrhage
- definite myocardial infarction within 1 year prior to randomization (definition of myocardial infarction, see Appendix IV).

3. certain other diseases :

- dementia
- acute hepatitis, or active cirrhosis or severe hepatic dysfunction
- malignant neoplasm
- diabetes mellitus is only excluded when frequent adjustments of insulin are necessary
- any physical condition prohibiting a sitting or standing position, or if the patient is permanently bed-ridden
- conditions necessitating to continue administration of drugs with blood pressure lowering activity, e.g. calcium antagonists, beta-blockers, diuretics (patients on treatment with nitrates may not be excluded)

4. poor collaboration : patients who without valid reason do not attend one exam during the placebo run-in period will be considered non-compliant and will not be randomized into the trial.

C. <u>The following conditions are not per se an exclusion criterion</u>

- patients with a cardiac pacemaker
- diabetes mellitus, satisfactorily controlled either with oral antidiabetic agents or with insulin
- patients on antidepressants, if in stable mental state

IX. STRATIFICATION OF PATIENTS

If at the end of the run-in period, the patient continues to satisfy the patient eligibility criteria, (s)he will be stratified by the responsible person in the Coordinating Office first by centre and in addition into one of the following four categories, combining gender and stage of hypertensive cardiovascular disease at randomization.

Stratum 1 : male patient without cardiovascular complications of hypertension;

Stratum 2 : female patients without cardiovascular complications of hypertension;

Stratum 3 : male patients with cardiovascular complications of hypertension;

Stratum 4 : female patients with cardiovascular complications of hypertension.

Conditions which are considered as cardiovascular complications of hypertension will lead to stratification into stratum 3 or 4. Examples are :

(1) history of angina pectoris; history of myocardial infarction; history of congestive heart failure; left ventricular hypertrophy

(2) history of TIA or cerebrovascular accident

(3) history of retinal exudates or papilloedema

(4) non-dissecting aortic aneurysm

Patient stratification and treatment randomization will be designed in such a fashion that in each participating centre a similar number of patients will receive active or placebo treatment (stratification per centre). For further details on the procedures to be followed see Appendix V.

X. DEFINITION OF ADMISSION INTO THE STUDY

During the run-in period the three record forms will be sent to the Coordinating Office. If additional data are required, the Coordinating Office will contact the Principal Investigator in the participating centre. If no additional data are required the Coordinating Office will communicate the patient identification number to both the local investigator and the Regional Drug Dispatching Centre (see Appendix V).

The patient is considered as having entered the study on the date on which (s)he is randomized by the Coordinating Office. This date should be as close as possible to the day when the double-blind medication is handed over to the patient.

Study medication will be supplied via Regional Drug Dispatching Centres (see Appendix V) covering one or more countries. The responsible person of the Regional Drug Dispatching Centre will be informed by the Coordinating Office whether a patient has been randomized to active medication or to placebo and will then send without further delay the study medication to the local investigators.

For each individual patient a closed envelope, containing the code of the double-blind medication, will be sent by the Coordinating Office to the collaborating clinical centre; this envelope should be kept closed and mailed back as such to the Coordinating Office at the end of the study, except in cases of major emergencies (see section XIII.2.C.).

XI. TREATMENT AFTER RANDOMIZATION

A. TREATMENT DURING THE DOUBLE-BLIND PERIOD

The following drugs will be employed :

a. In patients randomized to active treatment :

a1. First line medication : Hypotensor A

The first line active drug will be the calcium entry blocker nitrendipine. Scored tablets of 20 mg will be used with the following dosage steps :

* starting dose : half a tablet of 20 mg in the evening

* second step : half a tablet of 20 mg, twice daily (morning and evening)

* third step : one tablet of 20 mg, twice daily (morning and evening)

a2. Second line medication : Hypotensor B

The second line active drug will be the converting-enzyme inhibitor enalapril. Scored tablets of 10 mg will be used with the following dosage steps :

* starting dose : half a tablet of 10 mg in the evening

* second step : one tablet of 10 mg in the evening

* third step : two tablets of 10 mg in the evening

a3. Third line medication : Hypotensor C

The third line active drug will be the diuretic hydrochlorothiazide. Scored tablets of 25 mg will be employed with the following dosage steps :

* starting dose : half a tablet (12.5 mg) in the morning

* second step : one tablet (25 mg) in the morning

b. In patients randomized to placebo treatment :

In patients randomized to placebo study medication will solely consist of placebo tablets. Three different placebos will be used, namely Hypotensor A, Hypotensor B and Hypotensor C, respectively matching nitrendipine, enalapril and hydrochlorothiazide. For each of these placebos, tablets will be similar in shape, taste and appearance to the corresponding active drugs. Dosage steps in placebo-treated patients will also not be different from those employed in patients randomized to active medication.

The goal of treatment should be to reduce the sitting systolic pressure to a level below 150 mm Hg with at least a 20 mm Hg decrease in systolic pressure, as compared to the randomization blood pressure. This should be achieved by very progressive titration of the study medication. The dose of the blood pressure lowering study medication (active or placebo) should not be increased any further, if symptoms occur. During the titration phase of each drug, patients should be seen at a frequency dictated by clinical needs, but it is recommended that the interval between visits does not exceed one month.

The following rules apply equally to patients randomized to active treatment and placebo.

1. All patients should first be started on Hypotensor A alone, of which the dose should be progressively increased, if the goal systolic pressure is not achieved.

2. If a patient's systolic pressure is unsatisfactorily controlled by the maximum dose of Hypotensor A (active or placebo) alone, then Hypotensor B should be added to Hypotensor A and the dose of Hypotensor B progressively increased until the goal systolic pressure is reached. If necessary, half a tablet, and subsequently one tablet of Hypotensor C can be added to combined treatment with Hypotensor A and Hypotensor B.

3. If side-effects occur during monotherapy with Hypotensor A (active or placebo), the daily dose of Hypotensor A should first be reduced; if side-effects persist at this lower dose, treatment with Hypotensor A may be discontinued and Hypotensor B, the second line hypotensive agent, may be started. Similarly, Hypotensor B may be withdrawn because of side-effects and Hypotensor C started. Thus, in some patients, where either Hypotensor A or B had to be withdrawn Hypotensor C can be added to monotherapy with Hypotensor A or Hypotensor B. Hypotensor C may even be given alone, when both Hypotensor A and Hypotensor B had to be discontinued due to side-effects.

Side-effects will be reported on a quaterly basis to the Coordinating Office. However, severe or life-threatening adverse reactions must be immediately reported to the Coordinating Office. The Coordinating Office will then inform the Ethical Committee and the drug safety Liaison Officer of the manufacturer involved. The latter will then be responsible for informing, according to the manufacturer's legal obligations, national and international authorities.

In the unlikely event that during the course of the trial nitrendipine or enalapril should be withdrawn from general use for yet unknown toxic effects, the trial will be continued, using an alternative drug (and matching placebo) to be determined at that time with the agreement of all parties concerned.

Recommendations concerning non-pharmacological treatment are left to the discretion of the local investigators. However, in each centre they should be similar in the two treatment groups and independent of the blood pressure level.

B. TREATMENT DURING OPEN FOLLOW-UP

After the end of the double-blind period, surviving patients will be treated with whatever drug(s) the responsible physician feels necessary. However, the tablets of the double-blind period cannot be used any more.

XII. MEASUREMENTS DURING THE STUDY

Visits will be scheduled at least at monthly intervals during the run-in period of the study. After randomization, when a patient's blood pressure is stabilized, follow-up visits will be planned at three-monthly intervals. Data entry at the local centres and electronic transmission to the Coordinating Office through the EARN and JANET system will be developed, but is not compulsory. Patient charts are presented in Appendix VI.

A. <u>Reports required during the placebo run-in period</u>

Three visits are planned during the placebo run-in period at monthly intervals. Short report forms will be completed at the first and third follow-up visit. The report completed after two months on placebo will be the major initial record form.

A1. <u>First visit during placebo run-in period</u>

This visit should be scheduled after 1 month of placebo run-in treatment.

* Treatment information : daily intake of tablets; duration and reason of any interruption of study medication; concurrent medication (drugs and doses).
* Blood pressure and heart rate
* Blood tests are optional
* Decision to continue or not

A2. <u>Second visit during placebo run-in period</u>

This visit should be scheduled after 2 months of placebo run-in treatment.

A2/1. <u>History</u>

* Personal history : present and past diseases (year of last occurrence); surgical procedures (year of last occurrence); history related to previous antihypertensive treatment.

  For coding symptoms and diseases the ICD-Code revision nine will be used.

* Smoking habits and alcohol consumption

* Functional status of patients based on activities of daily living (ADL-score, see Appendix VII).

* Treatment information : see A1.

A2/2. Physical examination

* Blood pressure, heart rate, body weight, height

* Other physical signs

A2/3. Compulsory technical examinations

* Fundoscopy

* Twelve lead ECG (limited coding in each centre; original ECG to be sent to Coordinating Office). For further details see Appendix VIII).

* Urine tests : proteinuria; glucosuria; microscopy

* Blood tests (fasting) :

  - hemoglobin, hematocrit and red and white blood cell counts

  - serum creatinine; uric acid; cholesterol (total and HDL-cholesterol); triglycerides; alkaline phosphatase; SGPT; SGOT and/or gamma-GT; serum or plasma sodium and potassium

  - glucose

Results should be reported in SI units.

A2/4. Optional technical examinations

* Quality of life assessment by questionnaire (see part II)
* Twenty-four hour blood pressure monitoring (see part II)
* Twenty-four hour ECG monitoring (see part II)
* Storage of serum at -80°C (see part II)
* Study of multiple infarction dementia (see part II)
* Echocardiography (see part II)
* Chest X-ray
* Intravenous pyelography
* Renal angiography
* Determination of serum insulin concentrations

A2/5. Aetiological diagnosis and stage of hypertensive disease, according to the WHO-classification (see Appendix IX).

A2/6. Decision to continue or not in the run-in period

A3. Third visit during placebo run-in period

This visit should be scheduled after 3 months of placebo run-in treatment and is similar to the first visit on placebo. The decision should be made whether a patient may be randomized into the double-blind part of the study. A check list should be completed and sent to the Coordinating Office.

B. Reports required during the double-blind period

Short reports are planned at three-monthly intervals and more extensive reports yearly.

B1. Quarterly reports during the double-blind period of the study

These reports will be coded during the first year 1.1, 2.1, 3.1; during the second year 1.2, 2.2, 3.2; etc...
The report will include the following items :

* Treatment information : number of tablets anti-hypertensive agent A, B and C; any interruption of study medication; concurrent medication (drugs and doses), e.g. aspirin

* Blood pressure and heart rate

* New diseases and new findings since last visit; previous diagnoses and problems which are still active

* Blood tests : white cell counts, serum creatinine and potassium. These tests are compulsory during the first year of treatment with Hypotensor B (active or placebo), but may be omitted subsequently.

* Decision to continue or not

B2. Annual reports during the double-blind period of the study

These reports will be coded 4.1 for the first year; 4.2 for the second year and so forth.

B2/1. History

* Treatment information : see quarterly report
* Functional status of patient (ADL)
* Smoking habits and alcohol consumption

B2/2. Physical examination

Blood pressure, heart rate, body weight

B2/3. Compulsory technical examinations

Similar to second (extensive) visit during placebo run-in period; in addition a urine sample will be sent to a central laboratory (Prof. A. De Schaepdryver, Heymans Institute of Pharmacology, University of Ghent, Belgium) and analysed for sodium and creatinine. The urinary concentrations of the study drugs or their metabolites will also be measured in order to determine patient compliance. If no urine sample is sent it should be specified why not.

B2/4. Optional technical examinations (see Appendices I through VI)

* Quality of life assessment by questionnaire
* 24-h blood pressure and 24-h ECG recordings
* Storage of serum at -80°C
* Study of multiple infarction dementia
* Echocardiography
* Chest X-ray
* 24-h urine collection for measurement of creatinine and sodium

B2/5. Diagnoses : (1) new diagnoses and new findings since last visit; (2) previous diagnoses and problems, which are still active.

B2/6. Decision to continue or not : see section XIII.

C. <u>End of the double-blind period</u>

If the double-blind period is terminated in a particular patient, a report must be submitted giving the reason for stopping the double-blind study in this patient (see section XIII). This report will also contain the same information as the annual report form (see B2). A similar report will be submitted for all patients remaining in the study, when the trial as a whole is terminated.

D. Open follow-up after the double-blind period of the study

D1. Supervised open follow-up in patients who continue to attend the clinics

Patients whose double-blind period is terminated will be encouraged when possible to continue attending the clinics and (see section XIII) quarterly and annual report forms will continue to be completed.

D2. Non-supervised follow-up in patients who do not continue to attend the clinics

To allow an intention-to-treat analysis, follow-up should be available in all randomized patients. Thus, on patients who do not continue to attend the clinics the following information should be annually obtained by writing, telephone, or visits, either directly from the patients or via General Practitioners, family members or via Offices for Vital Statistics:

* patients alive or dead: if deceased, cause and date of death
* if patient is still alive:
    - treatment information
    - new diagnosis and new findings

XIII. END OF THE DOUBLE-BLIND PERIOD OF THE STUDY IN INDIVIDUAL PATIENTS

Patients will end the double-blind period of the study for one of the following reasons : (1) they experience a major fatal or non-fatal event (criteria for diagnosis see Appendix IV); (2) they are withdrawn from double-blind medication by the physician in charge without major event (withdrawals); (3) they are non-compliant to the protocol of the study (defectors); (4) or the termination date of the study has been reached (see section VI).

1. Major events

A. Death (use ICD code, ninth revision, 1977).
If an autopsy is performed, a copy of the protocol should be sent to the Coordinating Office.

B. Non-fatal but morbid cardiovascular and renal events :
These major events will be taken into account in the statistical analysis of trial endpoints.

B1. Cerebrovascular accidents, such as cerebral or subarachnoid haemorrhage or cerebrovascular thrombosis (ICD code 430-434, 436, [illegible])

Note : a T.I.A. (ICD code 435) is not a major event.

B2. Retinal exudates or haemorrhages or papilloedema (hypertensive retinopathy grade III or IV according to Keith-Wagener).

B3. Myocardial infarction (ICD code 410)

B4. Congestive heart failure, requiring either diuretics, or vasodilators, or any antihypertensive drug

B5. Dissecting aneurysm 4429

B6. Serum creatinine increasing 100 % above the initial value at 2 successive measurements or serum creatinine reaching the absolute value of 360 µmol/L (4.0 mg/dl) at 2 consecutive clinic visits

2. Withdrawals

A physician can withdraw a patient from the double-blind part of the study for the reasons mentioned below. These reasons will not be taken into account in the statistical analyses, which test the possible benefits of treatment.

A. A sitting systolic blood pressure exceeding on at least 3 consecutive visits 219 mm Hg and/or a sitting diastolic pressure exceeding on at least 3 visits 99 mm Hg, when the doses of the study drugs have been increased to their maximum. The interval between consecutive visits will depend on the clinical condition of the patient and can be shorter than 3 months, as normally scheduled.

B. Prescription of drugs with hypotensive action, because of conditions requiring a continuous long-term (3 months or more) therapy. Whenever possible, other treatments should be used in such circumstances. For instance, if angina pectoris develops during the trial, despite the prescription of all indicated study drugs, nitrates should be prescribed rather than beta-adrenergic blockers or calcium antagonists. Administration of the following drugs with hypotensive action for less than 3 months does not require withdrawal of a patient.

B1. diuretics

B2. beta-blockers

B3. calcium entry blockers

B4. rauwolfia derivatives

B5. converting enzyme inhibitors

B6. other drugs with hypotensive action

C. Any reason to break the individual medication code, such as complications possibly related to the double-blind medication.

Notes :

1. Any medical situation which can be adequately dealt with without breaking the code of the double-blind medication, e.g. by temporary interruption of the study treatment during no more than 3 months and administration of adequate medico-surgical therapy, should not lead to withdrawal of a patient from the double-blind part of the study.

2. Some complications during the double-blind part of the study may be specifically related to only one of the study medications.
This situation should not lead to withdrawal of a patient from the double-blind part of the study, but should be handled by withdrawal of the causative drug alone or of its matching placebo. Thus, in these cases the double-blind code should not be broken.

D. The doctor decides to stop the study medication because of an interfering disease, which is non-cardiovascular and non-fatal at that moment, e.g. malignant neoplasm.

E. Individual patients followed in the double-blind part of the study for seven years (eight years for patients recruited during the pilot trial, if the latter is continued into the main trial) (see section VI).

F. End of the whole trial for all patients (see section XIV).

The following conditions should be carefully monitored but are not a reason for terminating the double-blind part of the study :

* left ventricular hypertrophy
* multiple infarction dementia

3. Defectors

A patient is considered as a defector from the double-blind part of the study for two series of reasons :

A. If (s)he does not want to come back for a next appointment after 6 months or more; this is a decision of the patient. This condition includes :

- patient lives too far, has moved, is followed in another hospital
- patient does not want to attend the clinic again
- the patient finds transportation too difficult
- patient prefers another doctor

The reason why a patient does not want to continue in the study should be noted.

B. Interruption of all study treatment by the patient; this patient comes back for his (her) next visit, but does not want to continue any of the double-blind study medication. The exact reason for interruption should be noted. This is a decision of the patient. It includes non-compliance or the patient may prefer other ways of treatment rather than taking the double-blind study medication. These patients should be urged to try all study drugs or their combinations before finally stopping double-blind treatment.

XIV. STATISTICAL ANALYSES AND GUIDELINES FOR STOPPING THE TRIAL

(see Appendix X)

The trial may be stopped early, i.e. before the calculated number of patients and events have accumulated, because of a differential effect on morbidity and mortality between placebo and active medication. However, rigid stopping rules will not be used as they cannot reflect all possible outcomes that might arise during the course of the trial. Nonetheless statistical analyses will provide major guidance in the decision to stop the trial prematurely.

Four interim analyses and one final analysis will be performed. The interim analyses will not be scheduled at fixed intervals in time, but will be planned according to the number of major events accumulating during the course of the trial. Sample size calculations were based on a stroke rate of 20 events per 1,000 patient-years with a 40 per cent reduction on active treatment. After recruitment of 3,000 patients the trial is expected to be continued for 5 years. Over 5 years 150 stroke events are expected to occur in the placebo group and 90 events in patients on active treatment (240 events in total). Accordingly, interim analyses will be performed after the accumulation of 50, 100, 150 and 200 stroke events in the two treatment groups combined.

The statistic used to test the difference between the groups at each analysis will be the Cox Proportional Hazards Model ratio or the logrank statistic comparing two survival curves. These statistics will be employed with a group sequential method to provide monitoring boundaries. The O'Brien Fleming method (Appendix X) which gives greater weight to later occuring survival differences is appropriate and will be used. Two-sided boundaries will be calculated for overall significance levels of 10 %, 5 % and 1 %. The following endpoints will be considered : total mortality; stroke events (fatal and non-fatal); cardiovascular mortality; cardiac events (fatal and non-fatal myocardial infarction and congestive heart failure); fatal and non-fatal cardiovascular and renal (increase in serum creatinine by 100 % or above 360 µmol/L) events; and non-cardiovascular mortality. The Ethical Committee may consider stopping the trial, when at an interim analysis the 1 % boundaries for a benefit of active or placebo treatment or the 10 % boundaries for an adverse treatment effect are crossed.

Interim analyses will be based on an intention-to-treat evaluation of the data and the final analysis on an intention-to-treat, as well as on per-protocol comparisons.

PART II

SIDE-PROJECT IN
SYST-EUR

Side-project constitute an optional part of the main study. However if a centre decides to participate in (a) side-project(s), all patients entered at that centre into the main trial should also be followed in the framework of this (these) side-project(s).

I. ASSESSMENT OF QUALITY OF LIFE IN SYST-EUR

A.E. FLETCHER and C.J. BULPITT
Epidemiology Research Unit, Division of Geriatric Medicine, Royal Postgraduate Medical School, Hammersmith Hospital, Ducane Road, London W12 OHS.

I.A. RATIONALE

In previous major trials in hypertension, such as the Medical Research Trial of Mild to Moderate Hypertension (1), and the European Working Party Trial on Hypertension in the Elderly (2), the effects of treatment were measured in traditional terms of mortality, morbidity and symptomatic side effects. Patients completed questionnaires on the presence or absence of symptoms, but not on any resulting distress or interference with their daily lives. The rationale underlying the Quality of life approach is the assessment by the patients of the impact of ill health on their emotional, social and physical well-being and therefore provides a broad perspective of the effects of treatment (both active and placebo). This is particularly important in the elderly since any small reduction in health may result in a loss of independence. Quality of life measurements are therefore not just a better way of describing treatment side effects but include more serious consequences of hypertension such as stroke and coronary heart disease.

A rapidly growing literature on Quality of life suggests the importance of this concept in the field of health (3).

Quality of life measurements are considered essential in cancer trials (4) and are frequently incorporated into therapeutic trials in other diseases to prove a more comprehensive assessment of treatment outcome. The Systolic Hypertension in the Elderly trial in the United States has included measurements of Quality of life in the protocol (5).

I.B. AIM

B.1 Hypothesis to be tested

The null hypotheses is that there is no difference between placebo and active treatment on measures of Quality of life. The alternative hypothesis is that active treatment will produce an overall improvement in measures of Quality of life of 10 % compared to placebo.
The Quality of life measurement to be used for hypothesis testing is the Sickness Impact Profile (6) (SIP). This was developed over ten years ago by researchers at the University of Washington, Seattle, U.S.A. The questionnaire in its entirety consists of 136 statements describing the impact of ill health in different areas of everyday life and provides an overall score, physical score and psychosocial score as well as twelve separate scores for individual areas. In the Syst-Eur trial we have selected only those areas of the SIP relevant to the age group included and to the expected impact of disease during the trial (described in Section C.2 below).

B.2 <u>Sample size</u>

Assumptions concerning the initial SIP score (mean 21.0), change in score (2.1) and the standard deviation of the change (6.8) were based on a pilot study of 52 men and women age over 60 years attending a geriatric out-patient clinic or a hypertension clinic in the Hammersmith Hospital.
With a = 0.01, and 1-b (Power) = 0.9, 332 subjects in each group are required. Assuming an 80 % response rate, approximately 400 patients in each group will be required.

I.C. THE QUESTIONNAIRE

The questionnaire covers a variety of aspects of Quality of life and includes well established and tested scales.

C.1 <u>Section 1 is a short test of cognitive function : the Reitan Trial making Test (A and B) (7)</u>. In the first part of this test (A) subjects are required to connect up sequentially the numbers 1 to 25. The second part (B) consists of an alternating sequence of numbers and letters which subjects must connect up in the correct sequence. The time taken to correctly record the sequence is recorded. This test measures visual scanning, psychomotor speed and conceptual tracking, and has been shown to discriminate between the effects of captopril, propranolol and methyldopa (8) and between treated hypertensives in the community and their age-sex matched normotensive controls (9).

C.2 Section 2 consists of the following sections of the Sickness Impact Profile.

Ambulation : describes the effect of poor health on the ability to walk and move around freely.

Social Interaction : describes the effects of poor health on relationships with family and friends, and enjoyment of social activities.

Homework : describes the effects of poor health on housework, and other primarily physical activities related to looking after the home.

Sleep and Rest : describes the effect of poor health both on nighttime sleeping and daytime tiredness.

Each section includes a set of statements which are individually read to the patient. The patient is asked to respond if they agree with the statement.

C.3 Section 3 consists of a check list of 31 symptoms. The patients are asked to rate these on a 5 point scale according to whether they have experienced the symptom in the past week and if so the extent to which it has bothered them. There are also three separate questions which taken together describe postural hypotension.

C.4 Section 4 is the Brief Assessment Index (Card Version) (10). The patient is asked to respond to a statement on a card shown to them by the interviewer.

C.5 Acceptability of the Questionnaire

The Questionnaire was piloted in the geriatric out-patient clinic and hypertension clinic at the Hammersmith Hospital. Patients with a Hodkinson Mental Test Score (11) less than 6 were excluded (dementia). 14 patients who scored less than 6 on the Hodkinson check list, and 9 non-English speaking patients were excluded. 21 patients refused. 52 patients were interviewed (23 men and 28 women), with an average age of 74 years (range 60 to 95). The average time of completion of questionnaires was 40 minutes (range 20 to 80 minutes).

C.6 Administration of the Questionnaire

The questionnaire must be administered by a trained interviewer under standard conditions in the clinic. The reasons for choosing this method of questionnaire administration (instead of self completed) are :

(i) the cognitive function test must be administered by trained personnel,

(ii) self completed data may not be reliable in elderly subjects due to lack of familiarity with questionnaire methods such as checking of responses,

(iii) patients who cannot read or write for educational or health reasons can be included.

The interviewer is required to attend a session of 2 days in London, to be trained both in general skills of interviewing and specifically to administer the trial tests and questionnaire.

The standard conditions for questionnaire administration are : before seeing the doctor or having the blood pressure taken, and in a quiet room without interruptions.

C.7 <u>Visit dates for administration of the questionnaires</u>

The questionnaires should be administered at the following visits :

(i) Baseline. At randomization

(ii) After six months

(iii) After one year

Thereafter every year.

Patients who withdraw from the trial for any reason should also be interviewed as close to the time of withdrawal as possible. If these patients are missed the questionnaire data on Quality of Life will only be partially completed.

I.D. REFERENCES

1. Medical Research Council Working Party (1985) : MRC trial of treatment of mild hypertension principal results. Br Med J 1985; 291 : 97-104.

2. Amery A, Birkenhäger W, Brixko P et al. Mortality and morbidity results from the European Working Party on High Blood Pressure in the Elderly Trial. The Lancet 1985; 1349-1354.

3. Fletcher AE, Hunt BM and Bulpitt CJ. Evaluation of Quality of Life in Clinical Trials of Cardiovascular Disease. J Chron Dis 1987; 40 : 557-566.

4. Aaronson NK and Beckmann J. The Quality of Life of Cancer Patients. Monography Series of the European Organization for Research on Treatment of Cancer (EORTC). Volume 17. Raven Press.

5. Gurland BJ, Teresi J, McFate Smith W et al. Effects of Treatment for Isolated Systolic Hypertension on Cognitive Status and Depression in the Elderly. Am Ger Soc 1988; 36 : 1015-1022.

6. Bergner M, Bobbitt RA, Carter WB and Gilson BS. The Sickness Impact Profile as an assessment tool. Med Care 1981; 19 : 787-805.

7. Reitan RM and Wolfson. The Halstead-Reitan neuropsychological test battery. Theory and clinical interpretation. Neuropsychology Press, 1985.

8. Croog SH, Levine S, Testa M et al. The effects of anti-hypertensive therapy on the quality of life. N Engl J Med 1986. 314 : 1657-1664.

9. Battersby C, Hartley K, Fletcher AE et al. Cognitive Function in Hypertension, A Community Based Study. In preparation.

10. Beck AT et al. An inventory for measuring depression. Arch Gen Psych 1961; 4 : 561-567.

11. Hodkinson HM. Evaluation of a mental test score for assessment of mental impairment in the elderly. Age and Ageing 1972; 1 : 233-238.

II. QUALITY OF LIFE QUESTIONNAIRE

C O N F I D E N T I A L

SYST - EUR

QUALITY OF LIFE
QUESTIONNAIRE

(TO BE INTERVIEWER
ADMINISTERED)

II.A. SECTION 1

Trail making A

FORM A

EXAMPLE

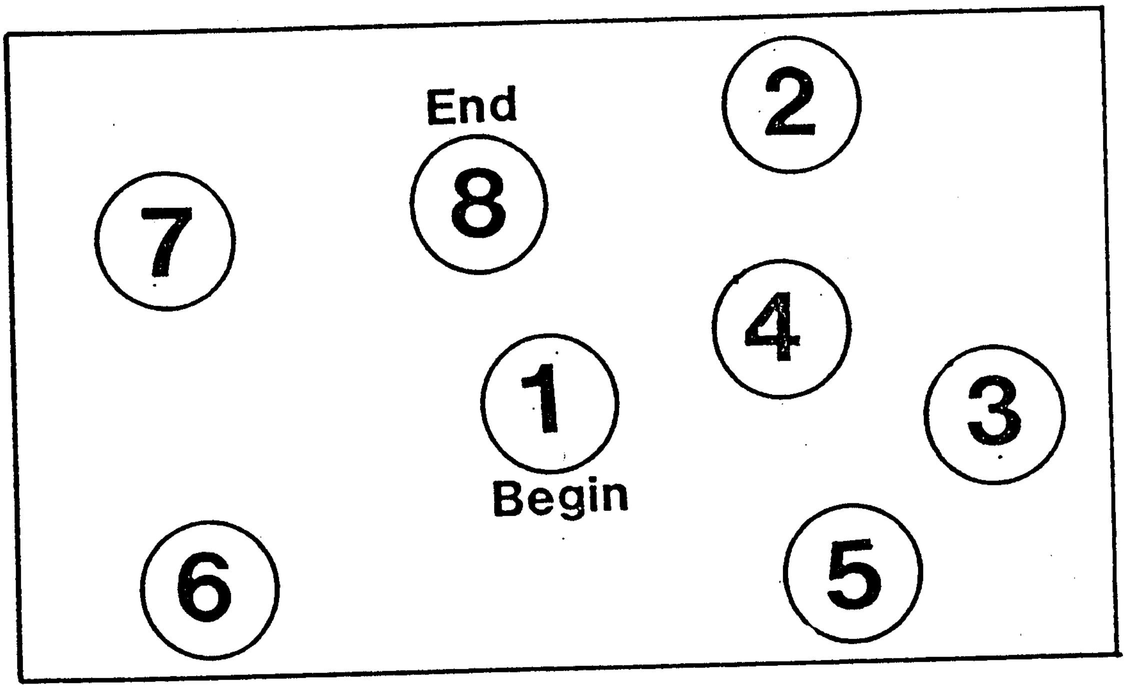

Trail making A

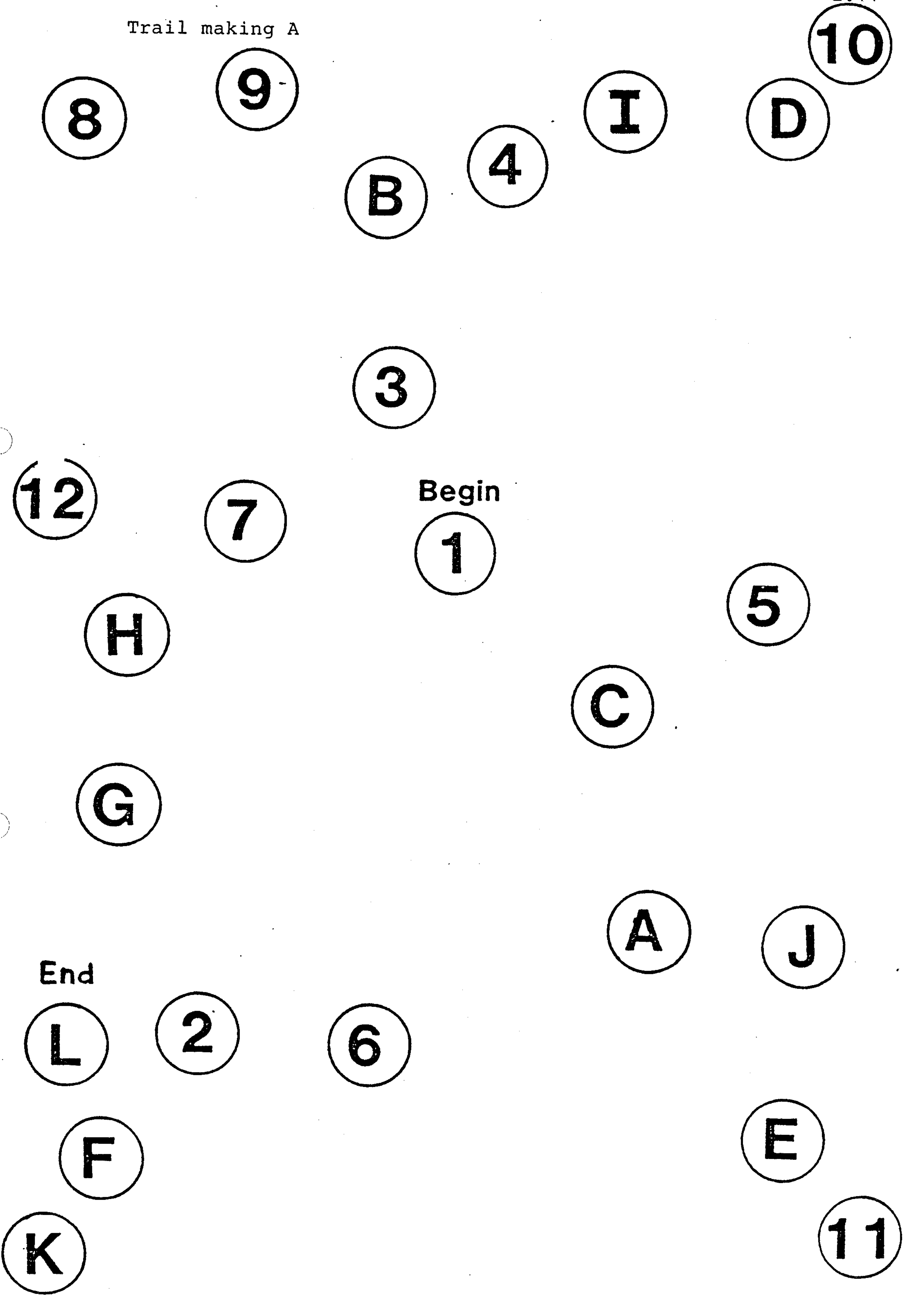

Trail making B

FORM B

EXAMPLE

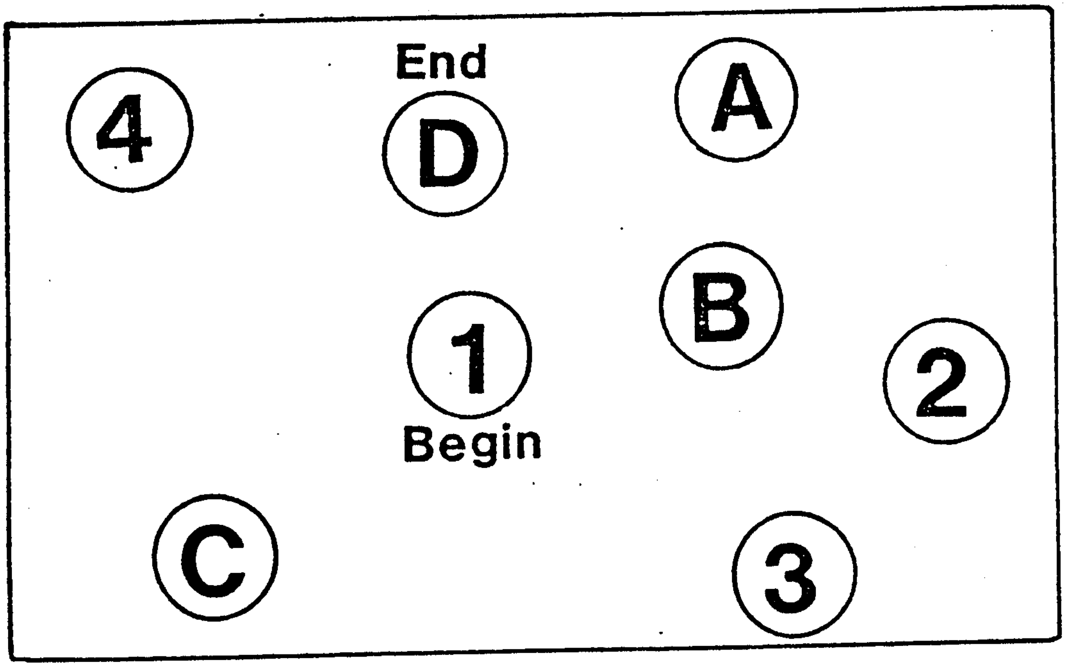

Trail making B

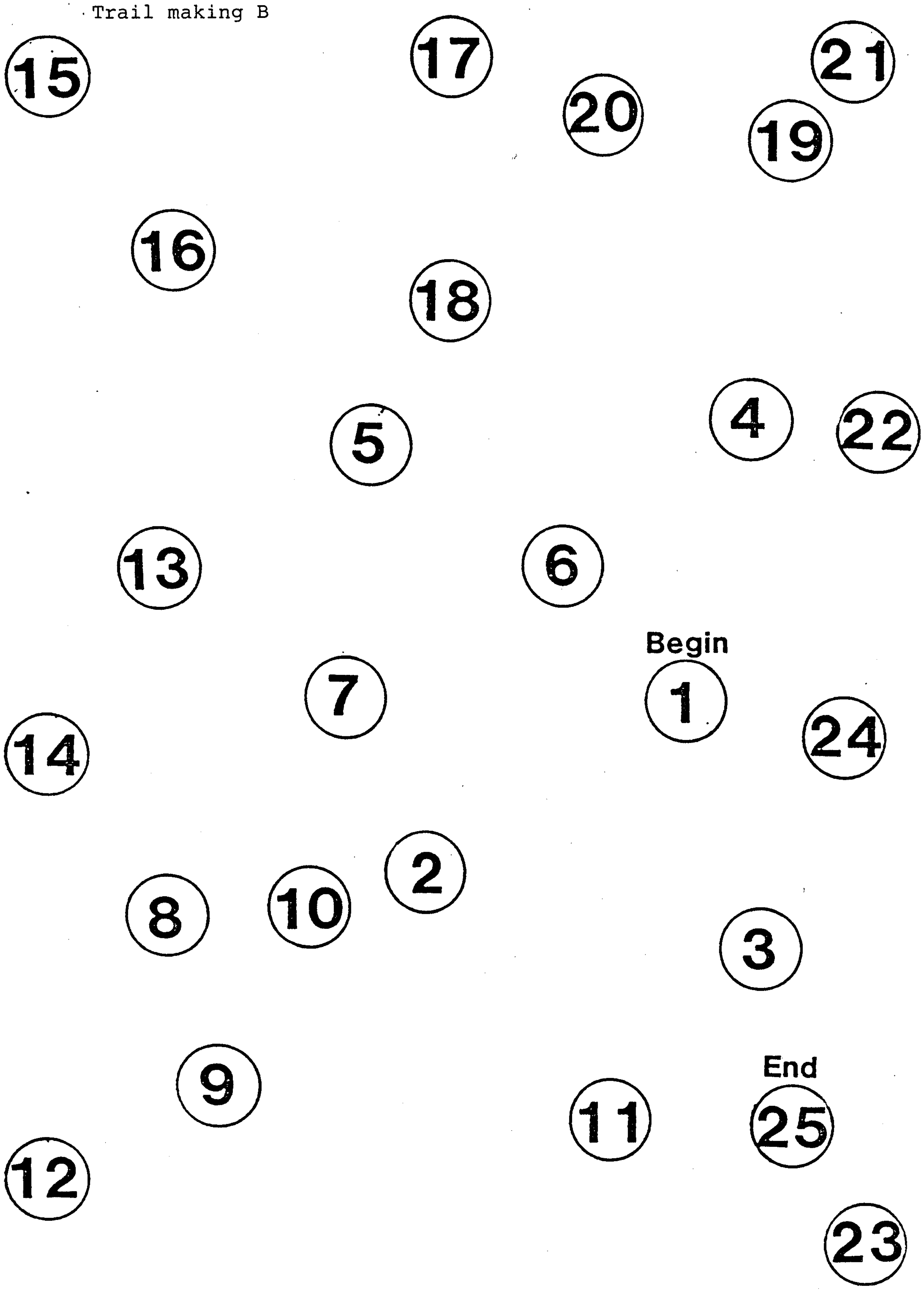

II.B. SECTION 2

Before beginning this section of the questionnaire, I am going to read you the instructions.

There are many activities which you carry out in daily life. Sometimes you do all of these activities, but at other times, because of the state of your health, you don't do these activities in your usual way : you may cut some out; you may do some for shorter lengths of time; you may do some in different ways. These changes in your activities might be recent or longstanding. We are interested in finding out any changes that describe you today, and which are related to your state of health.

I will be reading statements which people have told us describe them when they are not completely well. Whether or not you consider yourself to be ill, there may be some statements that will stand out because they describe you today and are related to your state of health. As I read the questionnaire, think of yourself today. I will pause briefly after each statement. When you hear one that does describe you and is related to health, please tell me and I will tick it.

Let me give you an example. I might read you the statement "I am not driving my car". If this statement is related to your health and describes you today, you should tell me. Also, if you have not been driving for some time because of your health, and are still not driving today, you should tell me.

On the other hand, if you never drive or are not driving your car today because it is being repaired, the statement "I am not driving my car" is not related to your health and you should not respond to it. If you are simply driving less than usual, or are driving shorter distances, so that you feel that the statement only partly describes you, please don't respond to it.

I am now going to begin the questionnaire. Please tell me if you want me to slow down, repeat a statement or stop so that you can think about it. Also let me know at any time if you would like to go over the instructions again. Remember that we are interested in the recent or longstanding changes in your activities that are related to your health.

AMBULATION

The following set of statements describe walking and use of stairs.

- I walk shorter distances or often stop for a rest. ☐
- I do not walk up and down hills. ☐
- I only use stairs with a physical aid, for example, a handrail, stick or crutches. ☐
- I only go up and down stairs with assistance from someone else. ☐
- I get about in a wheelchair. ☐
- I do not walk at all. ☐
- I walk by myself but with some difficulty, for example, I limp, wobble, stumble or have a stiff leg. ☐
- I only walk with help from someone else. ☐
- I go up and down stairs more slowly, for example, one step at a time or I often have to stop. ☐
- I do not use stairs at all. ☐
- I get about only by using a walking frame, crutches, stick, walls, or hold onto furniture. ☐
- I walk more slowly. ☐

SOCIAL INTERACTION

These statements describe your contact with your family and friends today.

- I go out to visit less often. ☐
- I do not go out to visit people at all. ☐
- I show less interest in other people's problems, for example, I don't listen when they tell me about their problems, I don't offer to help. ☐
- I am often irritable with those around me, for example, I snap at people or criticise easily. ☐
- I show less affection. ☐
- I take part in fewer social activities than I used to, for example, I go to fewer parties or social activities. ☐
- I am cutting down the length of visits with friends. ☐
- I avoid having visitors. ☐
- My sexual activity is decreased. ☐
- I often express concern over what might be happening to my health. ☐
- I talk less with other people. ☐
- I make many demands on other people, for example, I insist that they do things for me or tell me how to do things. ☐
- I stay alone much of the time. ☐
- I am dissagreeable with my family, for example, I act spitefully or stubbornly. ☐
- I frequently get angry with my family, for example, I hit them, scream or thorw things at them. ☐
- I isolate myself as much as I can from the rest of my family. ☐
- I pay less attention to the children. ☐
- I refuse contact with my family, for example, I turn away from them. ☐
- I do not look after my children or family as well as I usually do. ☐

I do not joke with members of my family as much as I usually do. ☐

HOMEWORK

These statements describe your daily work around the house.

I only do housework or work around the house for short periods of time or I rest often. ☐

I do less of the daily household chores than I would usually do. ☐

I do not do any of the daily household chores that I would usually do. ☐

I do not do any of the maintenance or repair work that I would usually do in my home or garden. ☐

I do not do any of the shopping that I would usually do. ☐

I do not do any of the cleaning that I would usually do. ☐

I have difficulty using my hands, for example, turning taps, using kitchen gadgets, sewing or doing repairs. ☐

I do not do any of the clothers washing that I would usually do. ☐

I do not do heavy work around the house. ☐

I have given up taking care of personal or household affairs, for example, paying bills, banking or doing household accounts. ☐

SLEEP AND REST

These statements are about sleep and rest.

I spend much of the day lying down to rest. ☐

I sit for much of the day. ☐

I sleep or doze most of the time - day and night. ☐

I lie down to rest more often during the day. ☐

I sit around half asleep. ☐

I sleep less at night, for example, I wake easily, I don't fall asleep for a long time or I keep waking up. ☐

I sleep or doze more during the day. ☐

II.C SECTION 3

I am now going to read you a list of words that describe symptoms people have. Choose from this list (ANSWERS A) the answer which describes whether you have experienced the symptom in the last week, and if so the extent to which it has bothered you.

		Not at all	A little	Moderately	Quite a bit	Extremely
1	Dry mouth					
2	Head pains or headaches					
3	Weakness of limbs					
[illegible]	Blurring of vision					
5	Shortness of breath					
6	Swollen ankles					
7	Constipation					
8	Bad taste in mouth					
9	Feeling of having burnt your mouth					
10	Blocked or runny nose					
11	Feeling sick					
12	Rash on body					
[illegible]	Itching					
14	Cramps in legs					
15	Pain in joints of hands					
16	Shaking hands					
17	Racing heart					
18	Stomach pain					
19	Heartburn					
20	Sore throat					
21	Dry cough					
22	Sweating more than usual					

		Not at all	A little	Moderately	Quite a bit	Extremely
23	Wheezing					
24	Dry eyes					
25	Mouth ulcers					
26	Light hurts your eyes					
27	Cold hands or feet					
28	Getting up at night to pass urine					
29	Diarrhoea					
30	Flushing of face					
[illegible]	Heart thumps or misses a beat					

32
In the last week have you suffered from unsteadiness, lightheadedness or faintness ?

☐ Yes

☐ No

If No, go to section 4.

33
Does the unsteadiness or faintness occur only when you are standing ?

☐ Yes

☐ No

34
Is the unsteadiness or faintness worse first thing in the morning ?

☐ Yes

☐ No

35
For how many hours a day are you troubled by unsteadiness or faintness ?

- ☐ Less than one hour
- ☐ 1-2 hours
- ☐ More than 2 hours

II.D SECTION 4

BRIEF ASSESSMENT INDEX

Show each card to the patient. Say to him/her -

I want you to say whether or not you agree to any of the following statements. Please answer YES if you agree and NO if you disagree.

Tick the box if they answer YES to the statement.

1. I feel anxious all the time. ☐
2. I've felt very low lately. ☐
3. I feel worse at the beginning of the day. ☐
4. I feel life is hardly worth living. ☐
5. I've cried in the past month. ☐
6. I've given up hope. ☐
7. I've seriously considered suicide. ☐
8. I can't recall feeling happy in the past month. ☐
9. I'm so lonely. ☐
10. I've lost interest in things. ☐
11. I'm too miserable to enjoy anything. ☐
12. I have regrets about my past life. ☐
13. I am a nuisance to others being ill. ☐
14. I've been depressed for weeks at a time in the past. ☐
15. I suffer headaches. ☐
16. I seem to have lost my appetite. ☐
17. I'm not sleeping well. ☐
18. I'm kept awake by worry and unhappy thoughts. ☐
19. I'm not happy at all. ☐

VERY OFTEN

QUITE OFTEN

OCCASIONALLY

VERY RARELY

NEVER

II. 24-HOUR BLOOD PRESSURE MONITORING IN SYST-EUR

A. Introduction

The variability of both systolic and diastolic blood pressure increases with age and is also greater in hypertensive as compared with normotensive subjects. Thus, it is possible that in elderly hypertensive patients sphygmomanometric measurements of blood pressure obtained at the out-patient clinic do not provide an accurate estimate of the degree of blood pressure elevation (Mancia G. et al., 1983). If this is true, the average of several pressure readings obtained in the patient's habitual environment may be more closely associated with the incidence of cardiovascular complications than the casual pressure observed during out-patient visits.

B. Aims of the study

(1) The principal goal of the present side-project is to test the hypothesis that not only casual blood pressure measurements obtained at the out-patient clinic, but also the ambulatory blood pressure predict the incidence of cardiovascular complications of hypertension. To investigate this hypothesis, office and ambulatory blood pressures, obtained during the placebo run-in phase of the Syst-Eur trial, will be correlated with the incidence of major cardiovascular-renal events, as defined in Section XIII of the main Syst-Eur protocol. Events during double-blind as well as during open follow-up will be considered, which

will enable both a per-protocol and intention-to-treat analysis. A similar project in middle-aged hypertensives is now being planned (Clement, 1989).

(2) A subsidiary goal of the side-project on the 24-hour blood pressure is to evaluate the possible harmful effects of excessive blood pressure lowering, when treatment decisions are based on the office blood pressure only (Waeber et al., 1987). Unexpected falls in blood pressure may be of particular interest as they may be correlated with subsequent cardiovascular events. This analysis will consider office and ambulatory blood pressure measurements obtained during placebo as well as active treatment.

(3) Finally, the side-project on ambulatory blood pressure also provides the opportunity to evaluate to which extent, in patients with a high systolic, but normal or low diastolic blood pressure, the systolic pressure remains elevated during an entire 24-hour period. This analysis will involve office and ambulatory blood pressure measurements during placebo treatment and will therefore include all patients during the placebo run-in phase of the trial, but only half of the patients after randomisation.

C. Sample size calculations

Side-projects are not compulsary. At the Syst-Eur investigator's meeting of 18 March, 1988, it was therefore recommended to perform sample size calculations to determine

how many centres would be required to test the main hypothesis of the side-project on ambulatory pressure with sufficient statistical power. It was also suggested to base sample size calculations on the study published by Perloff and co-workers (1983). In the latter study patients (n = 1,076) were classified according to the difference between their mean ambulatory blood pressure recorded at entry and their ambulatory pressure as predicted by linear regression from the mean office blood pressure at the start of the study. The average follow-up was 5 years. Based on life table analysis a significantly greater incidence of both fatal and non-fatal cardiovascular events occurred among patients with a higher than predicted ambulatory blood pressure than among those with a lower than predicted ambulatory blood pressure. Direct comparisons between the prediction by casual and ambulatory blood pressure measurements were not presented in Perloff's study, which precludes sample size calculations based on these results.

In the previous study by the European Working Party on High Blood Pressure in the Elderly blood pressure was only measured at out-patient clinics (Amery A. et al., 1981).

In the latter study systolic, but not diastolic pressure, at randomisation was positively correlated with the subsequent incidence of cardiovascular study terminating events and death from cardiovascular causes. The relationship remained statistically significant after adjustment for the effect of treatment, age, sex and the presence or absence of cardiovascular complications at randomisation. On the assumption

that in Syst-Eur the correlation of cardiovascular risk with the ambulatory systolic pressure will be of similar magnitude the present side-project will require recruiting from 600 to 800 patients.

D. Interval of 24-hour blood pressure recordings

Ambulatory blood pressure will be recorded over an entire period of 24 hours. Since for prediction the initial recordings will be extremely important, the ambulatory pressure will be measured twice during the placebo run-in period of the study, i.e. at the occasion of the second and third out-patient visit on placebo run-in treatment. Further recordings, when patients are being followed on double-blind treatment or in the supervised open phase of the study, will be scheduled six months after randomisation and at yearly intervals after randomisation.

When a 24-hour blood pressure recording is of insufficient quality, the investigator will repeat the recording before any adjustment of the treatment will have been made. A recording will be considered insufficient, when 20 per cent of the readings are either missing, or labeled as inaccurate by the software of the recorder, or show a diastolic pressure that is higher than the systolic.

E. Apparatus to be used

The use of a single type of apparatus by all centres was recommended in view of the homogeneity of the data and their later analysis by a single computer programme (Investigators'

meeting of 18 March, 1988). However, since Syst-Eur will last for several years and technical innovation in the field of ambulatory blood pressure monitoring is moving fast, it is unlikely that manufacturers will continue to produce a type of recorder that is being marketed at the start of Syst-Eur. In addition, assessing the ambulatory and office blood pressures in the prediction of cardiovascular risk constitutes the principal goal of the present study and does not necessarily imply the use of a single type of recorder. The use of several types of recorders may even increase the clinical applicability of the present side-project.

Only devices based on non-invasive techniques will be employed. Both Korotkoff sound detecting and oscillometric devices may measure blood pressure accurately and can be used in the present study (Syst-Eur investigators' meeting of 4 November, 1988). However, before being utilized in the study, recorders should have been validated according to the guidelines provided by the Association for the Advancement of Medical Instrumentation (Hope SL et al., 1988) or by the ad-hoc working group of the British Hypertension Society (E. O'Brien, personal communication, minutes of ABP-Committee meeting of 25 October, 1989).§

The blood pressure recorder to be utilized should enable non-invasive monitoring of blood pressure over at least 24-hour. Thus, it should be powered by batteries allowing an autonomy of at least 24-hours or 80 inflation. The cuff should be inflated

---

§ For a list of acceptable devices see Addendum I. (The list will be updated as new validation studies are published).

automatically and the timing of the inflations programmable over the day. As recommended by the British Hypertension Society a minimum bladder size of 35 x 12 cm must be used. Furthermore, it should be possible to check the calibration of the blood pressure monitor against an ordinary mercury column. Finally, a software package for data entry on a local (IMB-compatible) micro-computer should be available, unless the local investigator will enter the data manually.

F. Standardisation of the measurements

It is essential for the study that all centres involved have the necessary expertise for ambulatory blood pressure monitoring. For centres which do not have any previous experience a training session will be organized.

During the study the calibration of the devices should be checked according to the recommendations of the manufacturer, but at least at yearly intervals. Prior to each recording the arm circumference of the patient should be measured and the appropriate cuff size determined (minimum bladder size : 35 x 12 cm : see section E). Arm circumference and cuff size should be noted. The cuff may be secured either to the left or to the right arm of the patient, as most convenient for the subjects. Only when there is a systematic pressure difference between both arms of at least 10 mmHg by ordinary sphygmomanometry, the arm giving the highest pressure readings should be chosen. However, for each individual patient throughout the study the same cuff size should be used and recordings made as much as possible with

the same type of recorder and with the cuff secured to the same arm.

Patients or their relatives should keep a logbook during the blood pressure recordings. Each local centre will be allowed to use the type of logbook, with which it is familiar. It is important that the patients note in the logbook, as precisely as possible, the moment of awaking, defined as the first visual sensation after a night's sleep. Moreover, on the days of the ambulatory blood pressure recordings the doses and the times of administration of the study drugs should be similar, as compared with the days of the clinic visits, when the casual blood pressure readings are obtained. It is therefore strongly recommended to schedule ambulatory pressure recordings as close as possible to the second and third visit during the placebo run-in period, to the visit six months after randomisation and to the yearly visits during double-blind treatment and supervised open follow-up.

Throughout the 24-hour period the recorders will be programmed to obtain pressure measurements with an interval of 30 minutes. During the day the subjects will hold their arm in an extended position, if possible supported, during the pressure measurements. It is important that subjects are encouraged to wear the recorders for at least 25 hour so that full 24 hour pressure tracings can be obtained.

G. Analysis

At the local centres data will be entered on a micro-computer. For each recording the local investigator will complete a special record form "Report on 24-hour blood pressure". Within 1 to 3 months after the recordings, this record form, together with the ambulatory pressure measurements, will be sent to the Coordinating Office in Leuven. Data obtained during the placebo run-in phase will only be sent after it is known whether or not the patient will be randomized into the main study so that the Syst-Eur patient identificationnumber can be added to the files (see minutes of investigators' meeting of 25 October, 1989). All data obtained during the placebo run-in period, including those in patients who eventually were not randomized will be sent to the Coordinating Office.

Ambulatory pressure measurements will be sent as MS-DOS files by electronic transmission via modem or via the EARN system. Alternatively, the MS-DOS files can also be mailed on 3 or 5 inch diskettes which can be read by IMB-compatible micro-computer. The MS-DOS files should have the format of an ASCII, DBASE3, or SAS-file (see minutes of investigators' meeting of 25 October,1989).

Analyses will take place at the Coordinating Office and will involve daytime, nighttime (O'Brien E. et al.,1988) and overall averages of the ambulatory blood pressure. Editing of the data to exclude inaccurate results may be considered, but will, if necessary, only be performed at the Coordinating Office.

H. Collaborating centres

Participation in the side-project on 24-hour blood pressure monitoring is optional. However, if a centre decides to participate, all patients must be entered into the side-project. For a list of participating centres see Addendum II .

I. References

Amery A, Birkenhäger W, Brixko R, Bulpitt C, Clement D, Deruyttere H, De Schaepdryver A, Dollery C, Fagard R, Forette F, Forte J, Hamdy R, Henry JF, Joossens JV, Leonetti G, Lund-Johansen P, O'Malley K, Petrie JC, Strasser T, Tuomilehto J, Williams B. Efficacy of antihypertensive drug treatment according to age, sex, blood pressure, and previous cardiovascular disease in patients over the age of 60. Lancet 1986; ii : 589-592.

Clement DL on behalf of the Protocol Committee. Home versus Office Monitoring of Blood Pressure : a European study on high blood pressure. Protocol of the study. J Hypertension 1989, in press.

Hope SL, Alun-Jones E, Sleight P. Validation of the accuracy of the Medilog ABP non-invasive blood pressure monitor. J Ambulatory Monitoring 1988; 1 : 39-51.

Mancia G, Bertinieri G, Grassi G, Parati G, Pomidossi G, Ferrari A, Gregorini L, Zanchetti A. Effects of blood pressure measurement by the doctor on patient's blood pressure and heart rate. Lancet 1983; ii : 695-698.

O'Brien E, Sheridan J, O'Malley K. Dippers and non-dippers. Lancet 1988; ii : 397.

Perloff D, Sokolow M, Cowan R. The prognostic value of ambulatory blood pressures. JAMA 1983; 249 : 2792-2798.

Petrie JC, O'Brien ET, Littler WA, de Swiet M. Recommendations on blood pressure measurement. Brochure published by the British Medical Journal, 1987.

Waeber B, Scherrer U, Petrillo A, Bidivilli J, Weisberger J, Waeber G, Hofstetter JR, Brunner HR. Are some hypertensive patients overtreated ? A prospective study of ambulatory blood pressure recording. Lancet 1987, ii : 732-734.

ADDENDUM I

EVALUATION OF 24- HOUR AMBULATORY BLOOD PRESSURE MEASURING DEVICES

Eoin O'Brien

The Blood Pressure Unit
Beaumont Hospital
Dublin 9

The validation of blood pressure measuring devices is a complex and labour-intensive procedure and this is particularly so with ambulatory devices. The American Association for the Advancement of Medical Instrumentation (AAMI) has made recommendations for validation of electronic and automated sphygmomanometers and the British Hypertension Society has drawn up a protocol with detailed recommendations for device validation.

Published validation studies of ambulatory systems have been reviewed to identify the ambulatory devices that have been assessed according to the AAMI standard.

The names of ambulatory devices were determined from a review of the literature and validation studies were identified from the literature. Manufacturers were also written to requesting the cost of devices and a list of all validation work conducted on their respective devices. Though many manufacturers have produced a number of different devices, each being a modification of the basic model. obsolete models which have been superceded by newer versions have not been reviewed and current devices available on the market have been considered. As SpaceLabs Inc. is presently introducing a new model (the 90207) and as the manufacturers state that the algoarithm of the 90207 is no different to the 90202, validation studies for both models have been assessed.

Data from abstracts of meeting proceedings have not been included in the this analysis except when no other published work is available.

Sixteen reports of validation procedures (1-16) on 8 ambulatory systems were assessed. Only two devices have been assessed by the AAMI criteria - the SpaceLabs 90202, and the Medilog. Both fulfilled the criteria of the standard and are, therefore, the only systems that can be recommended at present for 24-hour ambulatory measurement of blood pressure.

We are aware that validation studies are presently being conducted according to the AAMI standard on the SpaceLabs 90207, the Accutracker II, the Novocor DIASYS, the Del Mar Avionics Pressurometer IV and the Takeda TM-2420. The results of these studies will be reported to SystEur so that the Recommendations contained in this Addendum can be up-dated at regular intervals.

REFERENCES

SpaceLabs 90202

1. Santucci S, Steiner D, Zimbler M, James GD, Pickering TG.
Validation study of the SpaceLabs 90202 and 5200 ambulatory blood pressure monitors.
Journal of Ambulatory Monitoring 1988;1:211-216.

2. Graettinger WF, Lipson JL, Cheung DG, Weber MA.
Validation of portable noninvasive blood pressure monitoring devices: Comparisons with intra-arterial and sphygmomanometer measurements.
Am Heart J 1988;**116**:1155-1160.

3. von Polnitz A, Hofling B.
Validation of the SpaceLabs Model 90202, a non-invasive, ambulatory blood-pressure monitoring device: intra-arterial and mercury column comparison study.
Journal of Ambulatory Monitoring 1989;2:169-173

4. Santucci S, Cates EM, James GD, Schussel YR, Steiner D, Pickering TG.
A Comparison of Two Ambulatory Blood Pressure Monitors, the Del Mar Avionics Pressurometer IV and the SpaceLabs 90202.
Am J Hypertens 1989;**2**:797-799.

5. O'Brien E, Sheridan J, Atkins N, Mee F, O'Malley K.
Validation of the SpaceLabs 90202 according to the AAMI standard.
Submitted for publication.

SpaceLabs 90207

6. Cates EM, Schussel YR, James GD, Pickering TG.
A validation study of the SpaceLabs 90207 Ambulatory Blood Pressure Monitor (Abstract).
Am J Hypertension 1989;**2**:41A.

Pressurometer IV

7. Germano G, Damiani S, Ferrucci A, Ciavarella M, Romeo F, Calcagnini G.
Reliability and reproducibility of a new device for ambulatory blood pressure recording.
Ann. Med In. 1988;**139**:140-141

8. Germano G, Ferrucci A, Donatella P, Pecchioli V, Napoleoni MG, Strano S.
Sulla ripoducibilita delle registrazioni di un nuovo apparecchio di monitoraggio ambulatoriale della pressione arteriosa.
Cardiologia 1987;**32**:743-746.

2. *Graettinger WF, Lipson JL, Cheung DG, Weber MA.
Validation of portable noninvasive blood pressure monitoring devices: Comparisons with intra-arterial and sphygmomanometer measurements.
Am Heart J 1988;*116*:1155-1160.*

4. *Santucci S, Cates EM, James GD, Schussel YR, Steiner D, Pickering TG.*
A Comparison of Two Ambulatory Blood Pressure Monitors, the Del Mar Avionics Pressurometer IV and the SpaceLabs 90202.
Am J Hypertens 1989;2:797-799.

Accutracker II

9. Torre LB, Rouse IL, Beilin LJ.
Reproducibility of 24-hour non-invasive ambulatory blood-pressure monitoring using the Suntech Accutracker.
Clin Exp PH 1989;16:247-251.

10. Baig MW, Wilson J, Wade G, Lonsdale D, Perrins EJ.
Clinical evaluation of the Accutracker ambulatory blood-pressure monitoring system.
Journal of Ambulatory Monitoring 1989;2:175-182

Medilog ABP

11. Hope S.L, Alun-Jones E, Sleight P.
Validation of the accuracy of the Medilog ABP Non-invasive blood -pressure monitor.
Journal of Ambulatory Monitoring 1988;1:39-51

Diasys 200

12. De Gaudemaris R, Deloraine A, Courturier P, Parat S, Jalbert M, Cugnardey N, Siche JP, Mallion JM.
Ambulatory blood pressure monitoring: comparative evaluation of 4 systems (Devices, Instruments). (Avionics PIV, Novacor Diasys 200, SpaceLabs 90202, Takeda TM2420).(Abstract).
Am J Hypertension 1989;2:121A.

Takeda TM-2420

13. Tochikubo O, Minamisawa K, Miyajima E, Masao I, Yanaga A, Yukinari Y.
A New Compact 24-hour Indirect Blood-Pressure Recorder and Its Clinical Application.
Jap Heart J 1988;29:257-269.

14. Russell AE, Tonkin AL, Wing LMH, Hassam RM, McRsritchi RJ, Aylward PE, Minson RB, Bune AJC, West MJ, Chalmers JP.
Accuracy of the Takeda TM-240 ambulatory blood pressure monitor.
Clin Exp PH 1989;16:253-256.

Colin ABPM 630

15. White WB, Lund-Johansen P, McCabe EJ.
Clinical evaluation of the Colin ABPM 630 at rest and during exercise: an ambulatory blood pressure monitor with gas-powered cuff inflation.
J Hypertension 1989;7:477-483.

Physioport

16. Langewitz W, Dahnert A, Ruddel H.
Zur Validitat der Blutdruckmessung eines neuen tragbaren automatischen BlutdruckmeBgerates (Physioport).
Die Medizinische Welt 1987;38:816-821.

ADDENDUM II : PROVISIONAL LIST OF CENTRES

- The following centres have agreed to participate on 25 October, 1989.

* Prof. D. CLEMENT
  Universitair Ziekenhuis
  Afdeling Cardiologie
  De Pintelaan 185
  B-9000 GENT
  Belgium

  tel. 32/91/40.34.81

* Dr. HOMUTH
  Zentralinstitut für Herz- Kreizlauf Forschung
  der Akademie des Wissenschaften der DDR
  Wiltbergstrasse 50
  1115 Berlin-Buch
  D.D.R.

* Prof. G. LEONETTI and Prof. G. MANCIA
  Centro di Fisiologia Clinica e Ipertensione
  Ospedale Maggiore
  Via F. Sforza 35
  20122 MILAN
  Italy

  tel. 39/2/551.33.60

* Prof. E. O'BRIEN
  Beaumont Hospital
  Dublin 9
  Ireland

  tel. 44/1/37.77.55

* Prof. Dr. E. RITZ
Department of Nephrology
University of Heidelberg
6900 HEIDELBERG
Federal Republic of Germany

* Prof. Dr. J.L. RODICIO
Hospital 1° Octubre
Carretera Andalucia
km 5.400
28041 MADRID
Spain

tel. 34/1/260.27.83

* Dr. P. de LEEUW
Inwendige Geneeskunde
Zuiderziekenhuis
Groene Hilledijk 315
N-3075 EA ROTTERDAM
Nederland

tel. 31/10/439.59.11

* Prof. Dr. J.B. ROSENFELD
Beilinson Medical Center
PETAH TIKVA 49100
Israel

tel. 972/3/922.16.05

* Prof. Dr. R. FAGARD
Inwendige Geneeskunde
Universitair Ziekenhuis Gasthuisberg
Herestraat 49
B-3000 LEUVEN
Belgium

tel. 32/16/21.36.31

* Prof. Dr. P. DUPONT
C.H. de Tivoli
Département de Néphrologie et d'Hypertension
34, Avenue Max Buzet
7100 LA LOUVIERE
Belgium

tel. 32/64/21.11.61

SYST-EUR

REPORT ON 24-HOUR BLOOD PRESSURE

IDENTIFICATION & REGISTRATION (1)

Syst-Eur patient number : ☐☐☐ ☐ ☐☐☐ ☐☐

Patient	Centre
Name or initials : (2)	Centre name : ....
Date of birth : ☐☐ day ☐☐ month ☐☐ year	Name of doctor : (2) ....

INFORMATION ON RECORDING (3)

Phase of study ☐

1 = run-in
2 = double-blind
3 = open follow-up

START

Date ☐☐ day ☐☐ month ☐☐ year

Hour ☐☐ hour ☐☐ min.

END

Date ☐☐ day ☐☐ month ☐☐ year

Hour ☐☐ hour ☐☐ min.

Type of recorder . ...

....

Arm to which cuff was secured ☐

1 = right,
2 = left,
9 = unknown

Arm circumference ☐☐ cm

Bladder size ☐☐ width X ☐☐ length cm

STUDY MEDICATION DURING RECORDING (3)

Hypotensor 1111 (run-in period)

Dose ? (tablets/day) ☐ ▪ ☐

Hour(s) of intake

— morning ☐☐ h. ☐ min.

— evening ☐☐ h. ☐ min.

Hypotensor A

Dose ? (tablets/day) ☐ ▪ ☐

Hour(s) of intake

— morning ☐☐ h. ☐ min.

— evening ☐☐ h. ☐ min.

Hypotensor B

Dose ? (tablets/day) ☐ ▪ ☐

Hour(s) of intake

— morning ☐☐ h. ☐ min.

— evening ☐☐ h. ☐ min.

Hypotensor C

Dose ? (tablets/day) ☐ ▪ ☐

Hour(s) of intake

— morning ☐☐ h. ☐ min.

— evening ☐☐ h. ☐ min.

(1) This report should be completed for each 24 h. blood pressure recording.
(2) Please print.
(3) Please, give hours in figures from 00 tot 24.

ANY OTHER DRUGS WITH POTENTIAL INFLUENCE ON BLOOD PRESSURE ?

Drug 1

Name ....

☐☐☐ / ☐☐☐☐☐

Drug 2

Name ....

☐☐☐ / ☐☐☐☐☐

Drug 3

Name ....

☐☐☐ / ☐☐☐☐☐

SYMPTOMS AND COMPLAINTS DURING RECORDING(1)

Did the patient note any of the following symptoms or complaints in his/her diary ?

☐ 1 = yes, 2 = no, 9 = unknown

If yes, specify symptom code and moment of occurrence :

Symptom code :
- 1 = dizziness, light-headedness, weakness, or equivalent
- 2 = fatigue, tiredness, or equivalent
- 3 = visual disturbances
- 4 = headache
- 5 = flushing, facial erythema
- 6 = nausea, vomiting
- 7 = palpitations, extrasystoles, tachycardia
- 8 = syncope

1. Symptom code ☐ Moment ☐☐ h. ☐☐ min.

2. Symptom code ☐ Moment ☐☐ h. ☐☐ min.

3. Symptom code ☐ Moment ☐☐ h. ☐☐ min.

4. Symptom code ☐ Moment ☐☐ h. ☐☐ min.

5. Symptom code ☐ Moment ☐☐ h. ☐☐ min.

---

(1) Please, give hours in figures from 00 to 24.

☐ for C.O.

ORIGINAL

MAIN MEAL DURING RECORDING (1)

☐☐ h. ☐☐ min.

SLEEP AT NIGHT (1)

START ☐☐ h. ☐☐ min.

(time patient went to sleep)

END ☐☐ h. ☐☐ min.

(first visual sensation after sleep)

BLOOD PRESSURE AT START OF RECORDING

Office blood pressure (mmHg)

		sitting (5min)
1	SBP	☐☐☐
	DBP (IV)(2)	☐☐☐
	DBP (V)	☐☐☐

		sitting (5min)
2	SBP	☐☐☐
	DBP (IV)(2)	☐☐☐
	DBP (V)	☐☐☐

DATA MANAGEMENT

Did you send the recording of the ambulatory BP to the C.O. ?

☐ 1 = yes, 2 = no

— If yes, specify date : ☐☐ day ☐☐ month ☐☐ year

— If yes, specify how ? ☐ 1 = File on diskette, 2 = ASCII file via EARN, 3 = ASCII file via modem,

— If not, indicate how the data will be sent :

....

....

....

COMMENTS

....

....

....

(1) Please, give hours in figures from 00 to 24.
(2) Where Korotkoff sounds persist below 50 mmHg, please give also phase IV DBP.

III. DETERMINATIONS ON STORED SERUM SAMPLES

This side-project consists of a study of biochemical variables, such as serum lipids and other indicators of cardiovascular risk; for this purpose serum should be stored at -80°C in centres participating in this undertaking. Serum samples will be collected at yearly intervals.

Protocol : to be written (Prof. De Schaepdryver).

IV. 24-HOUR ECG-RECORDING IN SYST-EUR

At the Syst-Eur investigators' meeting of 4 November it was decided not to develop a common protocol; however individual centres may include 24-hour ECG-recordings in their follow-up of Syst-Eur patients.

V. ECHOCARDIOGRAPHY IN SYST-EUR

R. Fagard, Hypertension and Cardiovascular Rehabilitation Unit, Leuven, Belgium.

In this section the reasons are given why it was decided not to develop a common protocol.

A. M-mode echocardiography of the left ventricle

1. References

(1) Savage DD et al. Considerations of the use of echocardiography in epidemiology. The Framingham study. Hypertension 9 (suppl) : II-40 - II-44, 1987.

(2) Wallerson DC, Devereux RB. Reproducibility of echocardiographic left ventricular measurements. Hypertension (suppl) : II-6 - II-18, 1987.

2. Acceptability of echocardiograms

. Inversely related to age : following data on the percentage of subjects with acceptable echos were obtained in the Framingham study (ref. 1, fig. 4) :

age	men	women
60-69 years	67	72
70-79 years	48	55
> 80 years	37	48

. However, a significant learning curve was apparent : in the original Framingham cohort the acceptance rate rose from a minimum of 28 % during the first five months of echocardiographic studies to a maximum of 74 to 81 % during studies 2 years later (ref. 1, fig. 2).

. Other factors were : gender, vital capacity, cardiovascular disease (ref. 1); in other studies obesity appears to be important.

3. Definition of an adequate echocardiogram

Echocardiograms are technically satisfactory if the following conditions are fulfilled : (1) generation of a single dominant line representing each interface being imaged, (2) demonstration of continuous interface lines at least 5 mm in length at the point of measurement, (3) demonstration of interfaces with the motion pattern characteristic of the specific cardiac structure being imaged, (4) simultaneous recording of QRS-complex with readily identifiable onset.

4. Technical factors in reproducibility

Patient position and transducer location greatly influence the echocardiographic image. The positions yielding the best measurable tracings on the initial echocardiographic study should be recorded accurately and applied in serial studies. Usual patient position : partial (30-45 degrees) left lateral decubitus (some laboratories use wedges) and 30-degree upright tilt of the head.
Usual transducer position : 3rd or 4th intercostal space, left sternal border, but in some patients 2nd (obesity) or 5th (long trunk) interspace yields the best image.

5. <u>Physiologic variability</u>

. respiratory variation (± 6 % in LVIDd) : recording at end-expiration is recommended;

. heart rate;

. preload, afterload, volume status.

6. <u>Echocardiographic measurement conventions (ref. 2, table 3)</u>

(1) Penn-convention

(2) American Society of Echocardiography

7. <u>Intra-observer and interobserver variability (ref. 2)</u>

(1) intra-observer or measurement variability : fluctuation of measurement on the same echocardiogram, when it is measured multiple times.

(2) interobserver variability (concurrent measurement variability) : interpretation of the same echocardiograms by multiple readers.

(3) the most advantageous combination of number of observers and number of readings per observer : two observers each reading each echocardiogram twice at different occasions or three observers, each reading each echocardiogram once.

8. <u>Temporal variability</u> (overall reproducibility; re-performance variability, ref. 2)

. Serial echocardiograms done on the same patient in a time period considered too short to allow any real change in the measured parameters.

. Ref. 2, fig. 3 : a change in LVIDd of 3 mm and in % D (fractional shortening) of 5.5 % or greater represents a biologically significant difference.

. paired reading of serial tracings on the same subject would reduce temporal variability by approximately 50 % (introduces possibility of observer bias when clue is available).

. two-dimensional guidance of M-mode echocardiograms does not result in greater interstudy reproducibility of measurements.

9. Equipment calibration

Ref. 2, page II-7.

10. Conclusions

(1) Adequate echocardiograms are not possible on all subjects; those with adequate tracings will not be representative of the total study population.

(2) Strict definitions should be established for :
. recording of tracings;
. acceptance of tracing;
. reading of tracings.

(3) The judgment on the acceptability of the echocardiograms and the reading of the tracings should be centralized.

B. Left ventricular Doppler velocimetry

If echocardiography is accepted in SYST-EUR left ventricular inflow Doppler velocimetry would be desirable to study left ventricular diastolic function.

C. Decisions adopted by the Working Party

At the meeting of January 22, 1988 it was decided to leave echocardiographic measurements to the discretion of each centre and not to develop a common protocol.

Fifth draft

22 December 89

SYST-EUR VASCULAR DEMENTIA SIDE PROJECT

Françoise FORETTE

A - INTRODUCTION

Dementia is a major problem in modern societies because of the size of their elderly populations.

Prevalence estimates from community surveys indicate that, on average, 5 % of persons over 65 years of age and probably 15 to 20 % of people over 80 have "severe" dementia. Alzheimer's disease accounts for about 50 to 60 percent of the cases and Multi Infarct Dementia (MID) due to cerebrovascular disease for about 10 to 20 %. Some patients have both disorders.

The incidence of MID reported in Western Population is around 7 per 1000 per year (1,2,3,4) and increases to nearly 16 per 1000 patients years in elderly patients "at risk" for cardiovascular disease (hypertension, hyperlipidemia, smoking...) (5). This incidence can be compared with the incidence of strokes as established by the USA National Survey (5.82 % to 18.24 %) (6).

It is reasonable to expect that, in Systeur, the incidences of MID and Strokes will be of similar magnitude.

Up to now, none of the therapeutic trials in hypertension has considered the issue of possible prevention of Multi-Infarct Dementia (MID) (vascular dementia) by antihypertensive treatment. The present side project aims to study MID as one of the important events occurring in elderly patients with systolic hypertension.

B - AIMS OF THE STUDY

1°) To test the hypothesis that antihypertensive treatment is able to reduce the incidence of Multi-Infarct Dementia.

2°) To follow the cognitive functions of elderly patients under active or placebo antihypertensive treatment.

C - DIAGNOSTIC PROCEDURES

1°) Mental status examinations have a key role in the identification of dementias. The Mini Mental State Examination of Folstein (M.M.S) is a brief, easily scored, test of several cognitive functions and should be administered yearly to all patients participating in the side project. (7)

The MMS assesses orientation to time and space, instantaneous recall, short term memory, operating mechanisms, constructional capacities and the use of language. The MMS may be properly administred by clinical personnel (physician, nurse...) with little training and does not usually require more than ten minutes for completion.

The MMS score is obtained by summing the points assigned to each successfully completed task for a total score ranging from 0 to 30. A score of 23 points or less indicates cognitive impairment. Only in these cases further diagnostic evaluation will be performed

2°) Diagnostic evaluation in patients whose MMS score is 23 or less.

The MMS by it self provides a valuable initial screen for dementia but cannot yield an accurate diagnosis without further evaluation.

The diagnosis of MID can be made with a reasonable degree of accuracy by using a group of validated criteria (8) :

Part I - MMS < 23 points

Part II.1 - Criteria fitting the definition of Dementia

as set by the DSM IIIR (Diagnostic and Statistical Manual of Mental Disorders) (A.P.A. 1987) (9).

Part II.2 - Modified Hachinski Ischemic Score (including CT Scan)(10) (11). (M.I.S)

These additional examinations can be easily performed by investigators experienced in dealing with demented patients (internist, geriatrician, neurologist...). Other investigators can acquire sufficient skill in a short time or, if necessary refer the patient to the appropriate center according to the available ressources.

Part II.1 and 2 will be seldom needed, given the expected incidence of MID (7 to 16 per 1000 person years). It will concern, at the very most one or two patients per center.

REFERENCES

1 - RORSMAN B., HAGNELL O., LANKE J.

Prevalence and incidence of senile and multi-infarc dementia in the Lundby study :

A comparison between the time periods 1947-1957 and 1957-1972.

Neuropsychology : 1986 : 15, 122-129

2 - NILSSON L.V.

The incidence of severe dementia in an urban sample followed from 70 to 79 years of age.

Acta. Psychiatr. Scand. : 1984. 70, 478-486

3 - MOLSA P.K., MARTTILA R.J., RENNE U.K.

Epidemiology of dementia in a finnish population

Acta Neurol. Scand. : 1982, 65, 541-552

4 - SCHOENBERG B.S.

Epidemiology of Alzheimer's disease and other dementing illnesses

J. Chronic. Dis. : 1986, 39, 1095-1104

5 - ROGERS R.L., MEYER J.S., MORTEL K.

Decreased cerebral blood flow precedes multi-infarct dementia but follows senile dementia of Alzheimer type.

Neurology : 1986, 36, 1-6

6 - Report on the National Survey of Stroke by the National Institute of Neurological and Communicative disorders and stroke.

Stroke, 1981, supplément N° 1, 12

7 - FOLSTEIN M.F., FOLSTEIN S.E., MC HUGH R.P.
"Mini Mental State" A practical method for grading the cognitive state of patients for the clinician
Journal of Psychiatric Research : 1975, 12, 189-198

8 - FORETTE F, HENRY JF, ORGOGOZO JM, DARTIGUES JF, PERE JJ, HUGONOT L, ISRAEL L, LORIA Y, GOULLEY F, LALLEMAND A, BOLLER F
Reliability of Clinical Criteria for the Diagnosis of Dementia
Arch. Neurol., 1989 : 46, 646-648

9 - AMERICAN PSYCHIATRIC ASSOCIATION :
Commitee on Nomenclature and Statistics.
Diagnostic and Statistical Manual of Mental Disorders.
Washington DC, American Psychiatric Association, Revised Edition, 1987

10 - HACHINSKI V.C., ILIFF L.D. and col
Cerebral blood flow in dementia
Arch. Neurol. 1975 : 32, 632-637

11 - LOEB C, GANDOLFO C
Diagnostic evaluation of degenerative and vascular dementia
Stroke, 1988, 19 : 560-565

SYST-EUR VASCULAR DEMENTIA SIDE PROJECT

PROTOCOL

Part I : "Mini Mental State" Examination (M.M.S)

To be completed at yearly intervals in all patients participating in the side project

Part II : Diagnostic Evaluation

To be completed only in patients whose MMS score is 23 or less

Part II-1 : DSM III Criteria for Dementia

See instructions page 11

Part II-2 : Modified ischemic score

See instructions page 14

SYST-EUR

"MINI-MENTAL STATE" EXAMINATION (MMS)

IDENTIFICATION & REGISTRATION (1)

Syst-Eur patient number : ☐☐☐ ☐ ☐☐☐ ☐☐

Date of visit : ☐☐ day ☐☐ month ☐☐ year

Phase of study : ☐

1 = run-in
2 = double-blind
3 = open follow-up

Patient	Centre
Name or initials : (2)	Centre name : ....
Date of birth : ☐☐ day ☐☐ month ☐☐ year	Name of doctor : (2) ....

A. ORIENTATION (Ask the following questions)

0 = incorrect
1 = correct
8 = not performed

What is today's date ?	Date	☐
What is the year ?	Year	☐
What is the month ?	Month	☐
What day is today ?	Day	☐
Can you also tell me what season it is ?	Season	☐
Can you also tell me the name of this hospital (clinic) ?	Hospital	☐
What floor are we on ?	Floor	☐
What town or city are we in ?	Town	☐
What county, state or department are we in ?	County	☐
What country are we in ?	Country	☐

B. IMMEDIATE RECALL

Ask the subject if you may test his/her memory. Then say : "**ball**", "**flag**", "**tree**", clearly and slowly. (one second for each word). After you have said all three words, ask him/her to repeat them. The first repetition determines his/her score (0-3) but keep saying them until he/she can repeat all three, up to six tries. If he/she does not eventually learn all three, recall (D) cannot be meaningfully tested.

Ball ☐

Flag ☐

Tree ☐

C. ATTENTION AND CALCULATION

1. Ask the subject to begin with 100 and count backwards by 7. Stop after five subtractions (93, 86, 79, 72, 65). Score the total numbers of correct answers.

93 ☐
86 ☐
79 ☐
72 ☐
65 ☐

If the subject cannot or will not perform the "count backwards test" task, then proceed to C2.

2. Ask the subject to spell the word "**world**" backwards. The score is the number of letters in correct position. For example, "**dlrow**" is 5, "**dlorw**" is 3.

d ☐
l ☐
r ☐
o ☐
w ☐

(1) To be completed at end of the run-in period, at yearly intervals during double-blind treatment, at end of double-blind treatment, at yearly intervals during open follow-up and at end of open follow-up.
(2)Please print.

ORIGINAL

D. RECALL

0 = incorrect
1 = correct
8 = not performed

Ask the subject to recall the three words you previously asked him/her to remember (B).

- Ball ☐
- Flag ☐
- Tree ☐

E. LANGUAGE

Naming

— Show the subject a wrist watch and ask him/her what it is.
— Repeat for pencil.

- Watch ☐
- Pencil ☐

Repetition

Ask the subject to repeat "**No ifs, ands, or buts**"

- Repetition ☐

3-Stage command

Give the subject a piece of plain blank paper and say :

"**Take the paper in your right hand, fold it in half, and put it on the floor**".

- Takes paper in right hand ☐
- Folds paper in half ☐
- Puts paper on floor ☐

Reading

Hold up the card which reads "**Close your eyes**", so the subject can see it clearly. Ask him/her to read it and to do what it says. Score as correct only if he/she actually closes his/her eyes.

- Closes eyes ☐

Writing

Give the subject a blank piece of paper provided and ask him/her to write a sentence. It is to be written spontaneously. It must contain a subject and a verb and be sensible. Correct grammar and punctuation are not necessary.

- Writes sentence ☐

Copying

On a clean piece of paper, draw intersecting pentagons, each side about 2 cm (as shown in the example below), and ask the subject to copy the drawing exactly as it is. All 10 angles must be present and two must intersect to score one point. Tremor and rotation are ignored.

- Draws pentagons ☐

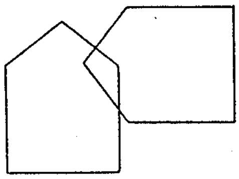

DERIVING TOTAL SCORE

Sum the number of correct replies to the test items. If item (count backwards) was not performed and if instead item C2 (world spelled backward) was used then add the number of correct letters given in proper position (zero to five).

Total Score (1) ☐
(Maximum 30)

(1) If total score is 23 points or less, further diagnostic evaluation is necessary. (See section C2 of protocol "Vascular Dementia side project")

ORIGINAL

SYST-EUR VASCULAR DEMENTIA SIDE PROJECT

TO BE COMPLETED ONLY IF MMS SCORE ≤23

IDENTIFICATION & REGISTRATION

Syst-Eur patient number : [ ][ ][ ] [ ] [ ][ ][ ] [ ][ ]

Date of visit : [ ][ ] day [ ][ ] month [ ][ ] year

Phase of study : [ ]
1 = run-in
2 = double-blind
3 = open follow-u

Patient	Centre
Name or initials :	Centre name : ....
Date of birth : [ ][ ] day [ ][ ] month [ ][ ] year	Name of doctor : ....

Site where the documents relevant to the diagnosis of dementia can be eventually consulted : ____

____

____

____

II. DIAGNOSIS OF DEMENTIA : DSM III R Criteria

Yes = 1
No = 2
Unknown = X

A - Impairment in short and long term memory [ ]

B - At least one of the following

1. Impairment of abstract thinking [ ]
2. Impaired judgment [ ]
3. Aphasia [ ]
4. Apraxia [ ]
5. Agnosia [ ]
6. Constructional difficulty [ ]
7. Personality changes [ ]

C - The disturbance in A and B significantly interferes with work or usual social activities or relationships with others.

D - The disturbance does not occur exclusively during the course of delirium.

E - The disturbance cannot be accounted for by any non organic mental disorder e.g. major depression.

The diagnosis of dementia implies a positive answer in A, at least one in B, and a positive answer in C, D, and E

Do you consider the patient demented ? Yes = 1
No = 2
Unknown = X

III. MODIFIED ISCHAEMIC SCORE (1) : To be filled in if the diagnosis of dementia has been reached.

1- Abrupt onset	Absent = 0	Present = 2	Unknown = X
2 - History of Stroke	Absent = 0	Present = 1	Unknown = X
3 - Focal symptoms	Absent = 0	Present = 2	Unknown = X
4 - Focal signs	Absent = 0	Present = 2	Unknown = X

5 - CT low density areas
Absent = 0
Isolated area present = 2
Multiple areas present = 3
Unknown = X

TOTAL SCORE : Add known scores for items 1 through 5
(scores 0,1, or 2; the sum of these scores may range from 0 to 10)

Score 0 to 2 —— suggests dementia of Alzheimer type
Score 5 to 10 —— suggests multi infarct dementia

Score 3 to 4 leaves the etiology of dementia unsettled

Please return these forms to the Coordinating Office in Leuven.

(1): Loeb C, Gandolfo C.
Diagnostic evaluation of degenerative and vascular dementia .
Stroke, 1988, 19 : 560-565

INSTRUCTIONS TO COMPLETE TO DSM III CRITERIA FOR DEMENTIA (Part II.1)

A - IMPAIRMENT IN SHORT AND LONG TERM MEMORY

* Impairment in short term memory : may be indicated by inability to remember three objects after five minutes.
e.g. : ball, flag, tree in part D of the MMS

* Impairment in long term memory : may be indicated by inability to remember :

- past personal information : date of birth
  place of birth
  occupation
- or facts of common knowledge : date of World War I
  date of World War II
  past president of the country

B -

B1 - IMPAIRMENT OF ABSTRACT THINKING

may be indicated by inability to find similarities and differences between related words, difficulty in defining words and concepts and other similar tasks.

e.g. : similarity test

. instruction : "I am going to name two objects which are similar in some respect and I want you to tell me in which way they are similar : for example, how are an orange and a banana alike (they are fruits).

. coat - dress
. eye - ear
. paper - radio
. car - bicycle
. dog - lion
. fly - tree (living beings)

B2 - IMPAIRMENT JUDGMENT

as indicated

- either by inability (reported by the family) to make reasonable plans to deal with interpersonal, family and job related problems and issues

e.g. : wear a fur coat in summer
go out of the house in pyjamas
make inconsiderate expenses out of proportions with one's means
cancel indipensable or compulsory insurance

- or inability to solve simple problems

e.g. : Does this sentence make sense : I have three brothers, Peter, Paul and me.

The railroad management has noticed that accidents affect mainly the last car. It has been therefore decided to get rid of the last car of every train : Do you think this will take care of the problem ?

B3 - APHASIA

(disorder of langage)

Suggested by word finding difficulties, use of inappropriate words and sometimes by difficulties in comprehension.

e.g. : In part E of the MMS, the patient cannot name watch or pencil even though he recognizes them and know their use. The patient may say: It is used to tell time, it is used to write.

B4 - APRAXIA

(inability to carry out motor activities despite intact comprehension or motor function)

e.g. : Difficulties in dressing, buttoning up one's shirt
can no longer use a domestic appliance

B5 - AGNOSIA

(failure to recognize or identify objects despite intact sensory functions)

e.g. : In part E of the MMS, the patient does not seem to recognize the watch or the pencil and cannot specify their use. He may loose the ability to recognize the face of family members or even his own face.

B6 CONSTRUCTIONAL DIFFICULTY

(inability to copy three dimensional figures, assemble blocks or arrange sticks in specific designs).

e.g. : cannot copy the two intersecting pentagons in the MMS

B7 PERSONALITY CHANGES

(alteration or accentuation of premorbid traits)

e.g. : an active patient becomes apathetic or a quiet person becomes agitated. On other hand, there may be an accentuation of preexisting personality trait : an autoritarian person becomes tyranical

C - THE DISTURBANCE IN A AND B SIGNIFICANTLY INTERFER WITH WORK OR USUAL SOCIAL ACTIVITIES OR RELATIONSHIP WITH OTHERS

e.g. : The patients experience major problems at work, may be fired or forced to an early retirement. Patients who were autonomous may become unable to live alone.

D - THE DISTURBANCE DOES NOT OCCUR EXCLUSIVELY DURING THE COURSE OF DELIRIUM

e.g. : some patients may present acute confusional episodes without being necessarily demented. On the other hand, demented patients may also present confusional states. Their deterioration continues when the confusional episode is over.

Only a definitive history of pre-existing dementia allows one to decide that an individual with delirium also has dementia.

E - **THE DISTURBANCE CANNOT BE ACCOUNTED FOR BY ANY NON ORGANIC MENTAL DISORDER e.g. MAJOR DEPRESSION**

This mean that non organic mental disorders such as depression have been reasonably excluded and that the specific organic factor (under the circumstances, the cerebrovascular process) is judged to be etiologically related to the disturbances.

INSTRUCTIONS TO FILL THE MODIFIED ISCHEMIC SCORE (Part II.2)

1 - Abrupt onset

The onset is typically abrupt (days or weeks) rather than uniformly progressive and the course is stepwise and fluctuating with rapid changes.

2 - History of strokes

Stroke (ICD code 430-431 or 436)

Acute disturbance of focal (or global) cerebral function with symptoms lasting for more than 24 hours or leading to death, with no apparent cause other than vascular. These codes include completed stroke as well as reversible ischaemic neurologic deficit (RIND). The latter is defined as an acute disturbance of focal neurological function with symptoms lasting for more than 24 hours.

3 - Focal symptoms (reported by the patient)

Focal symptoms may include :

* sensory deficit : numbness, abdnormal sensation in limbs, face occuring typically unilateraly
* motor deficit : hemiparesis, weakness of an arm or a leg
* episode of visual loss, hemianopsia...

4 - Focal signs may include : (observed by the doctor)

* any focal neurological deficit, such as unilateral motor weakness or sensory deficit, reflex asymmetries, extensor plantar response

Associated features :

* small stepped gait
* dysarthria, dysphagia
* pseudobulbar palsy with uncontrollable laughing and crying...

5 - CT low density areas

* large (infarcts) or small (lacunes) hypodensities
* unique or multiple
* unilateral or bilateral

These hypodensities are often associated with some degree of cortical atrophy and, less frequently with ventricular dilatations.

Patients with vascular dementia may also present extensive periventricular densities extending into the central white matter.

PART III

APPENDICES TO THE

PROTOCOL OF

SYST-EUR

APPENDIX I

LITERATURE REFERENCES TO SECTION II OF THE MAIN PROTOCOL

1. Kannel W.B., Gordon T.
Evaluation of cardiovascular risk in the elderly : the Framingham Study.
Bull. NY Acad. Med. 54 : 573-591, 1978.

2. Kannel W.B., Gordon T., Schwartz M.J.
Systolic versus diastolic blood pressure and risk of coronary heart disease : the Framingham Study.
Am. J. Cardiol. 27 : 335-346, 1971.

3. Staessen J., Cattaert A., Fagard R., Lijnen P., Vanhees L., Amery A.
Epidemiology of treated compared to untreated hypertention.
In : Hypertension. Eds. Genest J., Kuchel D., Hamet P., Cantin M., McGraw-Hill Book Company, New York, 1983, pp. 1069-1093.

4. European Working Party on High blood pressure in the Elderly (EWPHE).
An international trial of antihypertensive therapy in elderly patients : objectives, protocol and organization.
Arch. Int. Pharmacodyn. Ther. 275 : 300-334, 1985.

5. Amery A., Birkenhäger W., Brixko P., Bulpitt C., Clement D., Deruyttere M., De Schaepdryver A., Dollercy C., Fagard R., Forette F., Forte J., Hamdy R., Henry J.F., Joossens J.V.

Leonetti G., Lund-Johansen P., O'Malley K., Petrie J., Strasser T., Tuomilehto J., Williams B.
Mortality and morbidity results from the European Working Party on High blood pressure in the Elderly Trial.
Lancet i : 1349-1354, 1985.

6. Amery A., Birkenhäger W., Brixko P., Bulpitt C., Clement D., Deruyttere M., De Schaepdryver A., Dollery C., Fagard R., Forette F., Forte J., Hamdy R., Henry J.F., Joossens J.V., Leonetti G., Lund-Johansen P., O'Malley K., Petrie J., Strasser T., Tuomilehto J., Williams B.
Efficacy of antihypertensive drug treatment according to age, sex, blood pressure and previous cardiovascular disease in patients over the age of 60.
Lancet ii : 589-592, 1986.

7. Coope J., Warrender T.S.
Randomized trial of treatment of hypertension in elderly patients in primary care.
Br. Med. J. 293 : 1145-1151, 1986.

Firm guidelines for the treatment of systolic hypertension do not exist:

- 1986 Guidelines for the treatment of mild hypertension: Memorandum from a WHO/ISH meeting, Bull. WHO 1986, 64: 31-35.
- When and how to treat arterial hypertension. p. 55-60 in: Cardiovascular Care of the Elderly, T. Strasser, ed., WHO, Geneva, 1987.

APPENDIX II

ESTIMATED NUMBER OF PATIENTS REQUIRED FOR THE SYST-EUR TRIAL

Astrid Fletcher
Royal Postgraduate Medical School
Hammersmith Hospital
Du Cane Road
LONDON W12 OHS
United Kingdom

There are a variety of discussion points arising from the calculations.

I. Estimating the expected event rate in the placebo group

1. Rates from other studies

Data from a variety of population studies and trials (sub-groups and a pilot study) have been used to estimate the sample size. These provide the only published rates for subjects with Isolated Systolic Hypertension (ISH).

Limitations of the data are :

1.1. Age

Different studies include different age groups. Moreover, the variation in risk within these age groups is not presented. For example, little data is available on the risk for older patients (75+).

1.2. Sex

The data from the trials is not sex-specific, i.e., the rates are combined. However, the rates will

vary according to the proportions of men and women in the trial.

1.3. Level of blood pressure

Risk for levels of blood pressure above the cut off point is not available. ISH is defined as SBP > 160 and DBP < 90 for all studies and trials except :

a) Framingham (SBP ≥ 160 DBP < 95)

b) EWPHE (SBP > 160 DBP 90-95)

c) Coope and Warrender (SBP > 170 DBP < 90)

1.4. Population variation

Differences in total mortality suggest background population differences with "healthy" trial subjects experiencing a lower mortality. Geographical and social class variations in cardiovascular mortality account for some differences.

1.5. Reliability of the rates

Since the rates are derived from a small number of events, the sample size estimates based on the lower and upper confidence limits for the rates are also presented, assuming a Poisson distribution (95 % CL are not presented when the necessary data to calculate them is not given).

2. Temporal trends

Presented rates from various studies may not be realistic estimates of the expected rate in the SYST-EUR trial over

the next 5 years, due to temporal trends. CHD and cerebrovascular rates are falling in some European countries and rising in others, e.g., between 1950-1970 stroke mortality fell in England, Wales, Scotland, Israel and Finland, but rose in Portugal, Belgium and Ireland. In the U.S.A. there has been an acceleration in stroke mortality decline in the last few years. Reasons for the changes in stroke mortality are complex, but, relevant to this trial, it is probable that some of the changes relate to factors other than the treatment of hypertension.

Similar trends in CHD mortality have also been observed, and here it is likely that factors other than the treatment of hypertension are influencing these changes.

II. Choosing the appropriate event rate for the calculations

Cardiovascular disease is the major cause of mortality for subjects with ISH. The much higher rates for cardiovascular mortality compared to cerebrovascular mortality mean that fewer subjects would need to be randomized to detect significant reductions. However, several considerations argue against the choice of the cardiovascular mortality rate.

1. Previous trials have shown only marginal benefits in CHD reduction compared to stroke reduction, probably due to the overwhelming effect of other CHD risk factors, or,

perhaps, due to adverse treatment effects. If there was no reduction in CHD mortality, (proportionately greater than stroke) the benefits of a reduction in stroke mortality would not be detected.

2. The study would lack the power to examine separately the stroke and cardiac disease mortality, even if the cardiovascular mortality was reduced.

The incidence of fatal and non-fatal stroke has been shown to be reduced by antihypertensive treatment in most intervention studies. In elderly hypertensives the reduction in stroke incidence produced by active treatment averages 40 % (J. Staessen et al., Eur. Heart J., 1988, 9 : 215-222). Sample size calculations were therefore based on stroke rates.

III. Appropriate size of reduction

All calculations presented are based on an assumption of a rate reduction in the actively treated group of 40 %. This is of the order of stroke reduction seen in most trials.

IV. Appropriate power and significance level

Estimates of sample size are presented for different powers :

80 % 85 % 90 % 95 %

It is important to have a high power to be confident of detecting <u>no difference</u> between the two groups and therefore a power of less than 80 % was not considered. The significance was kept constant at 2 tailed p = 0.01.

V. Competing risks

The sample size estimates have been adjusted for losses to the cohort due to mortality from other causes. The underlying mortality in the various studies and trials has been used for this.

Sample size estimates for different populations and groups are presented on pages $A_{II.6}$, $A_{II.7}$, $A_{II.8}$, $A_{II.9}$, $A_{II.10}$, $A_{II.11}$, $A_{II.12}$.
These estimate the size of the control or the experimental group, i.e., NOT the sum of both.

Pc	is the observed event rate (number of events per person-year)
$I-\beta$	is the Power of the Test
LC_L	is the lower 95 % confidence limit of the event rate
UC_L	is the upper 95 % confidence limit of the event rate

RANCHO BERNARDO, CALIFORNIA COHORT, Garland et al., Am. J. Epidemiol. 1983, 118 : 365-376.

The following estimates are based on MORTALITY RATES for isolated systolic hypertension (SBP > 160 and DBP < 90) in 165 adults, aged > 60 years and with an average follow-up of 6.4 yrs. The underlying mortality is 219/1000 in men and 101/1000 in women.

STROKE

		Men			Women	Both		
		LC_L	RATE	UC_L		LC_L	RATE	UC_L
	Pc	0.011	0.041	0.104	No deaths from stroke	0.005	0.02	0.051
(1-β)	0.8	3238	839	307		6414	1567	592
	0.85	3615	937	342		7163	1750	662
	0.9	4120	1068	390		8164	1994	754
	0.95	4937	1279	468		9782	2389	904

CARDIOVASCULAR

		Men			Women		
		LC_L	RATE	UC_L	LC_L	RATE	UC_L
	Pc	0.04	0.089	0.169	0.004	0.019	0.056
(1-β)	0.8	861	365	174	7114	1472	478
	0.85	961	408	194	7944	1644	534
	0.9	1096	464	221	9053	1873	608
	0.95	1313	556	265	10849	2245	729

Both

		LC_L	RATE	UC_L
	Pc	0.028	0.054	0.094
(1-β)	0.8	1109	558	305
	0.85	1238	623	341
	0.9	1411	710	388
	0.95	1691	850	465

Framingham Study Cohort

Ref. Kannel et al., Circulation 1980, 61 : 1179-1182.

The following estimates are based on MORTALITY AND MORBIDITY RATES for isolated systolic hypertension (SBP > 160 and DBP < 95) in a cohort of men and women aged 55-74 years (ISH cohort size and number of events are not given). Average follow-up is 20 years. An underlying mortality of 56/1000 and 29/1000 in men and women respectively is used. 95 % CI could not be calculated.

CARDIOVASCULAR MORTALITY

		Men	Women
	Pc	0.0295	0.0244
(1-β)	0.8	856	987
	0.85	956	1102
	0.9	1090	1256
	0.95	1306	1508

CARDIOVASCULAR MORBIDITY

		Men	Women
	Pc	0.056	0.025
(1-β)	0.8	458	964
	0.85	508	1077
	0.9	576	1227
	0.95	694	1420

Framingham Study Cohort

Ref. Kannel et al., JAMA 1981, 245 : 1225-1229.

Subjects with SBP > 160 and DBP < 95.
The following estimates are based on stroke incidence rates in 544 men and 1295 women aged 50 to 79 years and followed for 24 years. Estimates are presented for the rates and for the 95 % CI of the rates. The underlying mortality of 70/1000 in men and 40/1000 in women is assumed.

		Men				Women		
		LC_L	RATE	UC_L		LC_L	RATE	UC_L
	Pc	0.0084	0.0147	0.024	Pc	0.0054	0.0085	0.0178
(1-β)	0.8	3168	1796	1089		4658	2949	1393
	0.85	3538	2006	1216		5202	3293	1556
	0.9	4032	2286	1386		5928	3753	1773
	0.95	4832	2739	1661		7104	4497	2124

Randomized controlled trials with ISH subgroups

EWPHE trial

These estimates are based on mortality data from 119 patients with SBP ≥ 160 and DBP 90-95 in the placebo group. The underlying mortality is 80/1000. 95 % CI are not given.

Cardiovascular mortality

Rate = 0.049

1-β	0.8	562
	0.85	628
	0.9	671
	0.95	857

Stroke mortality

Rate = 0.012

1-β	0.8	2226
	0.85	2486
	0.9	2834
	0.95	3395

Randomized controlled trials with an ISH subgroup

Treatment of hypertension in the elderly trial.

Ref. Coope and Warrender, BMJ 1986, 1145-1151.

These estimates are based on data from approximately 107 subjects with systolic pressure over 170 and DBP less than 90 randomized to control in the above trial. The age range was 60-79 years, and approximately two thirds were women. An underlying mortality of 34/1000 is taken.

Cardiovascular death rate

		LC_L	RATE	UC_L
	Pc	0.0372	0.0208	0.01021
(1-β)	0.8	678	1174	2420
	0.85	757	1311	2703
	0.9	863	1494	3080
	0.95	1034	1790	3691

These estimates are based on mortality and morbidity rates in 107 subjects (approximately 60 % women, and 60 % aged 70 years or more) followed for an average of 34 months. Rates and 95 % CI for rates are prevented. Underlying mortality is 22.7/1000.

Stroke events

		LC_L	RATE	UC_L
	Pc	0.00704	0.0192	0.0419
(1-β)	0.8	3445	1245	604
	0.85	3847	1391	675
	0.9	4385	1585	769
	0.95	5254	1899	922

Combined major events

(Stroke, left ventricular failure, myocardial infarction, and sudden death)

		LC_L	RATE	UC_L
	Pc	0.0198	0.0389	0.0671
(1-β)	0.8	1256	649	385
	0.85	1402	725	430
	0.9	1598	826	490
	0.95	1915	990	587

SUMMARY

Stroke and cardiovascular rates in different studies for subjects with ISH

Where male and female rates have been presented separately, they have been approximated as a single rate, using a weighting based on 1:2 male/female ratio.

	Cardiovascular Rate §		Stroke Rate §		Ages
SHEP Pilot Study	39		19	Fatal and NF	60+
Coope	21			Fatal	60-79
Rancho Bernardo	54		20	Fatal	60+
Framingham	26			Fatal	55-74
Framingham	36	55-74	11	Fatal and NF	50-79
EWPHE	49		12	Fatal	60+

§ Number of events per 1,000 person-years.

CONCLUSIONS

1. The cerebrovascular rate (CVA) should be the basis for calculation of the sample size: there is good evidence from trials on systolic and diastolic blood pressure reduction combined for a reduction by treatment of the stroke rate. However, in the present trial, where only systolic pressure will be lowered, the null hypothesis should be one of no difference and two-tailed tests should be used.

2. The CVA rate should include fatal and non-fatal events.

3. Assuming the ratio of fatal to non-fatal strokes is approximately 1:2, estimates of the fatal and non-fatal CVA rate range from 11/1000 (Framingham) to 60/1000 (Rancho Bernard). However the Framingham study includes younger subjects (50-79) and the Rancho Bernardo population may be unusual, with a high underlying mortality.
   Data from trials with the appropriate age range and BP level, and using similar approximations, suggest the rate may vary from 19/1000 (SHEP) to 36/1000 (EWPHE).

4. The proportion of men to women will be approximately 1:2 reflecting both the proportion in these age groups in the population, and those with ISH.

NUMBER OF PATIENTS NEEDED ACCORDING TO VARIOUS EXPERTS

Source	End point	Reduction	Power	2 p	Number of patients §	Expert
EWPHE (90-95 mm Hg)	Cardiovasc. mortality 49 %.	40 %	90	1 %	1342	AF
SHEP	Combined major events 39 %.	40 %	90	1 %	1652<br>1600<br>1672	AF<br>G<br>JS
SHEP	Stroke (fatal & non-fatal) 19 %.	40 %	90	1 %	3170<br>2999<br>3486	AF<br>G<br>JS
Coope (< 90 mm Hg)	Cardiovasc. deaths 20 %.	40 %	90	1 %	2988	AF

§ For both treatment groups.

Variation relates to differing assumptions about non-cardiovascular mortality and loss to follow-up.

APPENDIX III

GUIDELINES FOR BLOOD PRESSURE MEASUREMENT*

EQUIPMENT

A regular mercury sphygmomanometer will be used for blood pressure measurement. The sphygmomanometer must be checked to make sure that it is in good order. If any part of the apparatus is defective or unsuitable alternative equipment must be used. The following points should be checked :

Mercury column - The meniscus should be clearly visible, not obscured by oxidised mercury on the inside of the glass. Before inflation it must be at zero.

Cuff - The bladder, tubing, connections, inflation bulb, and valves should all be sound. The sheath containing the bladder should also be in good condition and have a secure fastening. Provided it is long enough to wrap round the arm and can be easily secured, the length of the sheath is not important.

The length of the bladder is one determinant of the area of pressure applied to the artery. If the bladder is too short the blood pressure will be overestimated, since the pressure is not fully transmitted to the artery. The bladder should nearly or completely encircle the patient's arm, and the length should be at least 80 % of the circumference of the arm. A 35 cm bladder is recommended. Most commercially available bladders are only 33 cm long.

* Proposal by E. O'Brien and adopted with slight modifications at the meeting of January 22, 1988.

The width of the bladder determines the length of the segment of artery to be occluded. Too narrow a bladder leads again to overestimation of blood pressure for the same reasons that too short a one does, but the error is not likely to be as great as that resulting from the use of bladders that are too short. A bladder width 12 cm is recommended.

Inflation-deflation device - Failure to achieve a pressure of 40 mm Hg above the estimated systolic blood pressure or 200 mm Hg after 3-5 seconds of rapid inflation is a sign of equipment malfunction. So too is the inability of the equipment to deflate smoothly when the controlling release valve is operated at 2-3 mm/s or at each pulse beat. The commonest source of error in the inflation-deflation system is the control release valve. Faulty control valves, dirty vents, and perished tubing should be replaced.

Stethoscope - the stethoscope should be in good condition with clean, well fitting earpieces.

Maintenance - sphygmomanometers should be checked for defects every six months.

PROCEDURE

The following practical points should be addressed :

Explanation to patient - the observer should outline the procedure briefly. In particular, he or she should warn the patient of the minor discomfort caused by inflation and deflation of the cuff and tell the patient that the measurement may be repeated several times.

Defence reaction - the defence reaction, causing an increase in blood pressure, tends to subside once the patient becomes accustomed to the procedure and the observer.

Position of arm - the arm should be horizontal and supported at the level of the mid-sternum because dependency of the arm below heart level leads to an overestimation of systolic and diastolic pressure of about 10 mm Hg. Correspondingly, raising the arm above heart level leads to underestimation of these pressures.

Application of cuff - the patient should be in a warm environment. Tight restrictive clothing should be removed from the arm. The position of maximal pulsation of the brachial artery in the arm, just above the antecubital fossa, should be palpated. A cuff with a long enough bladder should then be applied to the upper arm, with the tubing placed superiorly so that it does not interfere with auscultation. The lower edge of the bladder should be 2-3 cms above the marked point.

Position of manometer - the mercury column must be vertical, at eye level, and not more than three feet from the observer.

Estimation of systolic pressure - to detect the presence of an auscultatory gap, the systolic pressure should be estimated before the operator uses the stethoscope. The brachial artery pulse should be palpated and the cuff inflated for 3-5 seconds until the pulsation disappears. The point of disappearance represents the systolic pressure.

Auscultatory measurement of systolic and diastolic pressure - the stethoscope is placed gently over the artery at the point of maximal pulsation. The instrument must not be pressed too firmly or touch the cuff, or the diastolic pressure may be underestimated. The pressure is then raised by inflating the bladder to 30 mm Hg above the previously estimated systolic blood pressure. Next, the pressure is reduced at 2-3 mm Hg per second, or pulse beat. The point at which repetitive, clear tapping sounds first appear for at least two consecutive beats gives the systolic blood pressure. The point where the repetitive sounds finally disappear gives the diastolic blood pressure (phase 5). Both measurements should be taken to the nearest 2 mm Hg. In some elderly patients sounds may continue until the zero point. In such patients the final, distinct muffling of the repetitive sounds (phase 4) is taken as the diastolic pressure. If phase 4 is used this should be clearly recorded (200/90 mm Hg phase 4).

Digit preference - whereby observers choose to record, say, only to the nearest 0 to 5 mm Hg, is another source of bias. Such bias is best avoided by recording to the nearest 2 mm Hg.

Number of measurements - At each visit two measurements should be made in each position i.e. in the supine position after two minutes rest, in the sitting position after 5 min rest and in the standing position (after two minutes). If these readings do not agree to within 10-15 mm Hg a further reading should be taken. At least one minute should elapse between measurements. The mean of the two readings in each position is taken as the blood pressure for that visit.

<u>Measurement in both arms</u> - blood pressure should be measured in both arms at the initial assessment, and if there is a reproducible difference of 10 mm Hg for systolic pressure and 5 mm Hg for diastolic pressure the arm with the highest reading should be used for follow-up of patients.

<u>Times of measurement</u> - it is recommended that measurements are made not earlier than 2 hours after the last intake of medication.

<u>Training of personnel</u> - participants in the study should be familial with the techniques of blood pressure measurement and with the potential errors outlined above. It is recommended that those designated to measure blood pressure should have their technique checked by a trained observer and their accuracy measured using a double-headed stethoscope. For the training of staff involved in blood pressure measurements the film by Prof. Rose should be considered and circulated among the participating centres. These recommendations are based on those of the British Hypertension Society prepared by Professor J.C. Petrie, University of Aberdeen; Dr. E.T. O'Brien, The Charitable Infirmary, Dublin; Professor W.A. Littler, University of Birmingham; and Dr. M. de Swiet, Cardio-thoracic Institute, Brompton Hospital, London.

APPENDIX IV

DEFINITIONS OF CARDIOVASCULAR EVENTS*

1. Sudden death, cause unknown (ICD code 798)

Any death of unknown cause occurring instantaneously (798.1) or within an estimated 24 hours after the onset of acute symptoms or signs (798.2). Also unattended death (798.9) where the body of the deceased was found and no cause could be discovered and patients who are found death should be included.

If a cause can be identified e.g. by post mortem death should be coded accordingly and not classified as sudden death of unknown cause.

2. Myocardial infarction

2.1 Acute myocardial infarction (ICD code 410)

For the diagnosis of myocardial infarction at least two of the following three criteria must be met :

a) History of retrosternal pain with or without radiation to the shoulder, arms, jaws or abdomen. Pain should last for at least 30 minutes and not respond to nitroglycerine during the attack.

b) ECG abnormalities indicative of myocardial infarction : Minnesota Code numbers 1-2-1, 1-2-2, 1-2-3, 1-2-6, 1-3-1 through 1-3-6, 2-4, 4-0, 4-1, 5-1.

* Proposal by P. De Leeuw and adopted with slight modifications at the meeting of January 22, 1988.

c) Changes in the enzymes CPK, ASAT and LDH. Peak levels of CPK and ASAT must occur within 72 hours after onset of clinical symptoms; LDH must peak within 14 days. Preferably the levels of at least two of these enzymes should be elevated to twice the upper limit of normal or above.

A diagnosis of myocardial infarction may also be made when during the study ECG changes develop which denote definite myocardial infarction (Minnesota Code numbers 1-1-1 through 1-1-7, 1-2-4, 1-2-5, 1-2-7, 1-2-8), even without clinical symptoms or biochemical abnormalities.

2.2 Old myocardial infarction (ICD code 412)

this code will be used for coding myocardial infarction in the initial record forms.

3. Stroke (ICD code 430-434 or 436)

Acute disturbance of focal (or global) cerebral function with symptoms lasting for more than 24 hours or leading to death, with no apparent cause other than vascular. These codes include completed stroke as well as reversible ischaemic neurologic deficit (RIND). The latter is defined as an acute disturbance of focal neurological function with symptoms lasting for more than 24 hours.

4. Transient ischaemic attack (TIA) (ICD code 435)

Acute disturbance of focal neurological function with symptoms lasting for less than 24 hours and thought to be related to vascular disease.

Symptoms may include sensory defects, motor defects, amaurosis fugax, cortical blindness, hemianopsia, or speech disorders.

References

Stroke

Aho K, Harmsen P, Hatano S et al. Cerebrovascular disease in the community : results of a WHO collaborative study. Bull WHO 1980; 58 : 113.

TIA

Genton E, Barnett HJM, Fields WS et al. XIV. Cerebral ischemia : the role of thrombosis and on antithrombotic therapy. Joint Committee for Stroke Resources. Stroke 1977; 8 : 147.

Heyman A, Leviton A, Nefzger D. et al. XI. Transient focal cerebral ischemia : epidemiological and clinical aspects. Stroke 1977; 5 : 277.

APPENDIX V

RANDOMIZATION AND SUPPLY OF STUDY MEDICATION

I. RANDOMIZATION

For each centre and for each stratification group within each centre a list of patient numbers will be drafted (see Table I). With the use of a random numbers generator (SAS Language Guide for Personal Computers, Version 6 Edition, SAS Institute Inc, Cary, North Carolina page 73, SAS-function RANBIN) after stratification for centre, gender, and presence or absence of cardiovascular complications each patient number will be linked either to active study medication (A) or to placebo (P).

Table I : List of patient numbers

Centre 321 (Leuven)

Stratum I	000 (P) - 088 (A) - 202 (A) - 508 (P) - 621 (P) - 934 (A) - etc
Stratum II	365 (A) - 847 (P) - 309 (A) - 279 (A) - 523 (P) - 106 (A) - etc
Stratum III	782 (A) - 295 (P) - 518 (P) - 421 (P) - 529 (P) - 781 (A) - etc
Stratum IV	268 (A) - 736 (P) - 137 (P) - 846 (P) - 603 (A) - 452 (A) - etc

To randomize a patient the following procedures will be followed :

1. The list of patient numbers corresponding to the centre number and stratum will be employed.

2. From this list the lowest number, which has not yet been assigned to another patient will be given to the patient to be randomized.

3. The code corresponding to the patient number determines whether the patient will receive active study medication or placebo.

II. DISPATCHING OF STUDY MEDICATION

II.1. Procedures at the Central Drug Dispatching Centre of Bayer in Germany

- Tablets of Hypotensor A (active and placebo) will be manufactured by Bayer. Tablets of Hypotensor B (active and placebo) and of Hypotensor C (active and placebo) will be provided by Merck Sharp & Dohme. All drugs however will be packed and dispatched by Bayer. In addition to a Central Drug Dispatching Centre in Leverkusen, Bayer will therefore set up Regional Drug Dispatching Centres which will subserve one country or a small group of countries.

- Bayer Germany will fill bottles with Hypotensor A, Hypotensor B and Hypotensor C (active tablets and matching placebos).
  All bottles with study medication will be labeled with the name of the trial (SYST-EUR). In addition, according to the drug or the matching placebo they

contain, bottles will be labeled Hypotensor A, Hypotensor B or Hypotensor C. Each bottle of a particular Hypotensor will contain enough tablets for 35 days of treatment on the maximal daily dose, e.g. for nitrendipine (active or placebo) the maximal daily dose is 2 tablets and accordingly bottles with Hypotensor A will contain 70 tablets.

- In addition to the indication of the type of the Hypotensor the label of each bottle will also carry a bottle identification number. The Central Drug Dispatching Centre of Bayer Germany will provide for each Hypotensor lists with the bottle identification numbers (placebo and verum). This number will identify in a unique way each separate bottle allow for those who are entitled to access the code to make the distinction between placebos and tablets containing active drugs.

- Bottles with active and placebo study medication will be packed in separate boxes. Boxes with study medication and the corresponding lists with bottle identification numbers will be shipped by Bayer Germany to the Regional Drug Dispatching Centres. A copy of the lists with the bottle identification numbers will also be sent to the Coordinating Office in Leuven.

- When bottle identification numbers become too long one might envisage to use from a certain year on labels and lists with a different colour.

II.2. Procedures at the Coordinating Office in Leuven

- When a patient is being followed during the single-blind placebo run-in period of the study the local investigators will send to the Coordinating Office two short and one extensive report form. As soon as these three initial record forms are received at the Coordinating Office a patient will be considered for randomization.

- When a patient meets all criteria for entry into the study and does not present any condition listed as an exclusion criterion he/she will be randomized, after stratification for centre, gender and presence or absence of cardiovascular complications.

- Upon randomization the Coordinating Office will communicate the patient identification number to the local investigator and to the Regional Drug Dispatching Centres. This patient number will consist of 4 blocks of digits : (1) centre number = 3 digits; (2) one digit identifying the strata; (3) patient number = 3 digits; (4) two check digits (see section VII of main protocol).

The Coordinating Office will also instruct the Regional Drug Dispatching Centre whether a particular patient should be given active study medication or placebo. At the same time the Coordinating Office will send a sealed envelope with the code (active or placebo) for this particular patient to the local investigator. This envelope should only be opened in an emergency situation, which cannot be handled in another way (see section XIII.2 of main protocol).

II.3. Procedures at the Regional Drug Dispatching Centres

- The Regional Drug Dispatching Centres will receive from Bayer Germany boxes with Hypotensor A, Hypotensor B and Hypotensor C and lists with the bottle identification numbers. Bottles with Hypotensor A, Hypotensor B and Hypotensor C and bottles with active and placebo tablets will arrive at the Regional Drug Dispatching Centre packed in separate boxes and should also be stored on separate shelves.

- The Regional Drug Dispatching Centre will receive from the Coordinating Office in Leuven for each patient (1) a patient identification number and (2) the instruction whether a particular patient should be given active or placebo medication.

- The responsible person at the Regional Drug Dispatching Centre will write the patient identification number on the labels of the bottles with study medication. Bottles with Hypotensor A, Hypotensor B, and Hypotensor C, carrying both the patient and bottle identification numbers should be sent without any further delay to the local investigator. Enough study medication will be provided for one year of treatment on the maximal dose of Hypotensor A, Hypotensor B and Hypotensor C.

- The names of the local representatives in charge of the Regional Drug Dispatching Centres should be available at the Coordinating Office in Leuven (name, office and home addresses; office and home telephone numbers, if available, telefax number and EARN number). At any time a representative of the Coordinating Office may make a field visit to the Regional Drug Dispatching Centre to check their operating standards.

- Under no circumstance the Regional Drug Dispatching Centre shall disclose to local investigators whether a patient has been allocated to active study medication or placebo.

II.4. Procedures at the Local Centres where patients are being followed

- The local investigators willreceive from the Coordinating Office the patient identification number and a sealed envelope with the corresponding code. Study medication will be provided by the Regional Drug Dispatching Centre.

- The local investigator should check whether the patient identification number on the bottle labels corresponds with the patient number communicated by the Coordinating Office. The local investigator will then as soon as possible hand over the study medication to the patient.

- Local investigators will notify the Regional Drug Dispatching Centre that they need more medication for a patient when the supply of any study drug is insufficient for a period of three months. The Regional Drug Dispatching Centre will then ship the study medication for the following year.

III. ADDENDUM TO APPENDIX V : LABELS

III.1. Placebo run-in period

FOR CLINICAL TRIALS ONLY
MADE BY BAYER LEVERKUSEN

SYST-EUR
HYPOTENSOR A

1111

EXP Date

Pat.-ID.

III.2. Double-blind study

FOR CLINICAL TRIALS ONLY
MADE BY BAYER LEVERKUSEN

SYST-EUR
HYPOTENSOR A

Bottle N°

Batch N°

EXP Date

Pat.-ID.

Hypotensor B or C

Produced by MSD, USA

Packed by Bayer

APPENDIX VI

PATIENT CHARTS

SYST-EUR

LOGBOOK*

Centre name : ....

Centre number : ....

Logbook number : ....

* List all patients eligible for placebo run-in period and send logbook to Coordinating Office in Leuven every 6 months.

COPY

PATIENT<br>Name — First Name	Gender<br>M = male<br>F = female	Date of Birth	Date of Visit	Blood Pressure<br>SBP/DBP	Treatment at time of BP-recording (1)	Admitted in run-in period of trial (2)	Admitted in double-blind period of trial (2)
1		day month year	day month year				
2		day month year	day month year				
3		day month year	day month year				
4		day month year	day month year				
5		day month year	day month year				
6		day month year	day month year				
7		day month year	day month year				
8		day month year	day month year				
9		day month year	day month year				
10		day month year	day month year				

(1) Yes or no ; if yes, specify.
(2) Yes or no ; if no, specify reason.

ORIGINAL

A VI.3

SYST-EUR
SHORT REPORT DURING PLACEBO RUN-IN PERIOD

IDENTIFICATION & REGISTRATION (1) ☐☐☐ ☐ ☐☐☐ ☐☐

Visit : First or third visit (3)

Date of visit : ☐☐ day ☐☐ month ☐☐ year

Patient	Centre
**Name or initials :** (2)	**Centre name :** ....
**Date of birth :** ☐☐ day ☐☐ month ☐☐ year	**Name of doctor :** (2) ....

INFORMATION ON TREATMENT

Study medication at moment of visit

Hypotensor A

(1111)

Dose ? (tablets/day) ☐.☐
- 1/2 tablet per day = 0.5
- 1 tablet per day = 1.0
- 1 1/2 tablets per day = 1.5
- 2 tablets per day = 2.0

Dose reduction or interruption since last visit ? ☐
- 2 = no,
- 3 = yes, dose reduction
- 4 = yes, interruption,
- 8 = not applicable, 9 = unknown

If yes, reason(s) for dose reduction or interruption
1. .... ☐ ☐☐☐.☐
2. .... ☐ ☐☐☐.☐
3. .... ☐ ☐☐☐.☐

Any other drugs (e.g. aspirin, laxatives)

Drug 1

Name : .... ☐☐☐ / ☐☐☐☐☐

Drug 2

Name : .... ☐☐☐ / ☐☐☐☐☐

Drug 3

Name : .... ☐☐☐ / ☐☐☐☐☐

Drug 4

Name : .... ☐☐☐ / ☐☐☐☐☐

Drug 5

Name : .... ☐☐☐ / ☐☐☐☐☐

Drug 6

Name : .... ☐☐☐ / ☐☐☐☐☐

acco D1900A—1989

(1) To be completed at the first and third out-patient visit during the placebo run-in period.
(2) Please print.
(3) Circle correct answer.

☐ for C.O.

ORIGINAL

PHYSICAL EXAMINATION

Blood pressure (mmHg)		supine	sitting(5min)	standing(2min)
1	SBP			
	DBP (IV)*			
	DBP (V)			
2	SBP			
	DBP (IV)*			
	DBP (V)			

Sitting pulse rate (bpm)

DECISIONS & COMMENTS

Does the patient continue placebo run-in period ?(1)

1 = yes, 2 = no, 9 = unknown

If no, specify reason :

....

....

....

Comments :

....

....

....

(1) Please reply yes (code = 1) when after third run-in visit the patient is continued on placebo tablets before being randomized.

* Optional

for C.O.

ORIGINAL

SYST-EUR

EXTENSIVE REPORT DURING PLACEBO RUN-IN PERIOD

IDENTIFICATION & REGISTRATION (1) ☐☐☐ ☐ ☐☐☐ ☐☐

Visit : Second visit

Date of visit : ☐☐ day ☐☐ month ☐☐ year

Patient	Centre
**Name or initials : (2)**	**Centre name :** ....
**Gender :** ☐ M = male, F = female	
**Date of birth :** ☐☐ day ☐☐ month ☐☐ year	**Name of doctor : (2)** ....

LOGBOOK

Please indicate where the patient was entered in your logbook :

Logbook number : ☐☐ **Row number :** ☐☐☐

HISTORY

Personal history

Present diseases

1 .... ☐ ☐☐☐·☐

2 .... ☐ ☐☐☐·☐

3 .... ☐ ☐☐☐·☐

4 .... ☐ ☐☐☐·☐

5 .... ☐ ☐☐☐·☐

Past diseases and operations — **year of last occurrence**

1 .... ☐ ☐☐☐·☐ 19 ☐☐

2 .... ☐ ☐☐☐·☐ 19 ☐☐

3 .... ☐ ☐☐☐·☐ 19 ☐☐

4 .... ☐ ☐☐☐·☐ 19 ☐☐

5 .... ☐ ☐☐☐·☐ 19 ☐☐

Family history

Is father deceased ? ☐ 1 = yes, 2 = no, 9 = unknown

If yes : age of death ☐☐☐ years

cause of death ....

.... ☐ ☐☐☐·☐

Is mother deceased ? ☐ 1 = yes, 2 = no, 9 = unknown

If yes : age of death ☐☐☐ years

cause of death ....

.... ☐ ☐☐☐.☐

(1) To be completed at the 2nd visit during the placebo run-in period.

(2) Please print.

☐ for C.O. ORIGINAL

DIAGNOSIS OF HYPERTENSION (HT)

Stage of HT : ☐

1 = HT without organ involvement
2 = HT with LVH only.
3 = HT with MI or angina only.
4 = malignant HT
5 = HT with CNS involvement only.
6 = HT with renal involvement only.
7 = HT with eye fundus grade III only.
8 = HT with multiple organ involvement.
9 = unknown.

Aetiological diagnosis of HT :

Essential HT : ☐ 1 = yes (certain), 2 = no, 3 = possible, 4 = unlikely, 9 = unknown

If secondary HT, specify cause :

....

☐ ☐☐☐ . ☐

SMOKING & DRINKING HABITS

Is patient ex-smoker ? ☐

Is patient smoking now ? ☐

1 = yes,
2 = no,
9 = unknown

If yes, give consumption per day of :

cigarettes	cigars	pipes
☐☐	☐☐	☐☐

Does the patient consume alcohol now ? ☐

1 = yes,
2 = no,
9 = unknown

If yes, how many glasses per week of :

beer	wine
☐☐☐	☐☐☐
**spirits**	**apéritif or fortified wine**
☐☐☐	☐☐☐

ACTIVITIES OF DAILY LIVING (ADL)

if "yes" score = 1 if "no" score = 0

Can the patient bath alone ? ☐

Can the patient dress alone ? ☐

Can the patient go to the toilet alone ? ☐

Can the patient move in and out bed and chair alone ? ☐

Is the patient entirely self-controlled for urination and defaecation ? ☐

Can the patient eat alone ? ☐

Total ADL score (sum of 6 scores) : ☐

EDUCATION

Patient followed full-time school until age : ☐☐

INFORMATION ON TREATMENT

Previous antihypertensive treatment

Was the patient on treatment with antihypertensive drugs during **the last 6 months** ?

☐ 1 = yes, 2 = no, 9 = unknown

If yes, specify if the patient was on one of the following antihypertensive drugs :

1 = yes,
2 = no,
3 = unknown

— Centrally acting drugs ? ☐

— Diuretics ? ☐

— Beta-blockers ? ☐

— Calcium entry blockers ? ☐

— ACE-inhibitors ? ☐

— Other antihypertensive drugs ? ☐

Study medication at moment of visit

Hypotensor A
(1111)

Dose ? (tablets/day) ☐ . ☐

1/2 tablet per day = 0.5
1 tablet per day = 1.0
1 1/2 tablets per day = 1.5
2 tablets per day = 2.0

Dose reduction or interruption since last visit ? ☐

2 = no,
3 = yes, dose reduction
4 = yes, interruption,
8 = not applicable,
9 = unknown

If yes, reason(s) for dose reduction or interruption :

1 ....
☐ ☐☐☐ . ☐

2 ....
☐ ☐☐☐ . ☐

3 ....
☐ ☐☐☐ . ☐

☐ for C.O.

ORIGINAL

Any other drugs (e.g. aspirin, laxatives)

Drug 1

Name : ....

☐☐☐ / ☐☐☐☐☐

Drug 2

Name : ....

☐☐☐ / ☐☐☐☐☐

Drug 3

Name : ....

☐☐☐ / ☐☐☐☐☐

Drug 4

Name : ....

☐☐☐ / ☐☐☐☐☐

Drug 5

Name : ....

☐☐☐ / ☐☐☐☐☐

Drug 6

Name : ....

☐☐☐ / ☐☐☐☐☐

PHYSICAL EXAMINATION

Blood pressure (mmHg)		supine	sitting(5min)	standing(2min)
1	SBP	☐☐☐	☐☐☐	☐☐☐
	DBP (IV)*	☐☐☐	☐☐☐	☐☐☐
	DBP (V)	☐☐☐	☐☐☐	☐☐☐
2	SBP	☐☐☐	☐☐☐	☐☐☐
	DBP (IV)*	☐☐☐	☐☐☐	☐☐☐
	DBP (V)	☐☐☐	☐☐☐	☐☐☐

Sitting pulse rate (bpm) ☐☐☐

Weight (kg) ☐☐☐.☐

Height (cm) : ☐☐☐.☐

Fundoscopy — **Right eye** ☐ **Left eye** ☐

(stage Keith-Wagener)

1 = grade I, 2 = grade II, 3 = grade III, 4 = papilloedema,
5 = lens opacity, 8 = normal, 9 = unknown

Other physical signs ? ☐ 1 = yes, 2 = no

If yes, specify :

1 .... ☐ ☐☐☐.☐

2 .... ☐ ☐☐☐.☐

3 .... ☐ ☐☐☐.☐

4 .... ☐ ☐☐☐.☐

5 .... ☐ ☐☐☐.☐

URINE TESTS

Proteinuria	☐	1 = positive, 2 = traces, 3 = negative, 9 = unknown
Glucosuria	☐	
White blood cells	☐	1 = many (≧ 10 per field), 2 = rare, 3 = none, 9 = unknown
Red blood cells	☐	
Granular casts	☐	

BLOOD TESTS

Was patient fasting ? ☐ 1 = yes, 2 = no, 9 = unknown

Haematocrit (%)	☐☐.☐
Haemoglobin (mmol/L)	☐☐.☐
WBC (10^9 cells/L)	☐☐.☐
RBC (10^{12}cells/L)	☐.☐☐
Creatinine (µmol/L)	☐☐☐.☐
Uric acid (mmol/L)	☐.☐☐
Na (mmol/L)	☐☐☐.☐
K (mmol/L)	☐.☐☐
Glucose (mmol/L)	☐☐.☐
Cholesterol : — total (mmol/L)	☐☐.☐
— HDL (mmol/L)	☐☐.☐
Triglycerides (g/L)	☐☐.☐
Alk. phosph. (U/L)	☐☐☐
SGOT (U/L)	☐☐☐
SGPT (U/L)	☐☐☐
gGT (U/L)	☐☐☐
Insulin (µU/ml)*	☐☐☐

* Optional

☐ for C.O.

ORIGINAL

ELECTROCARDIOGRAM

ECG normal ? ☐ 1 = yes, 2 = no, 8 = not performed, 9 = unknown

If abnormal, specify : ....

....

....

Original sent to C.O. on ☐☐ day ☐☐ month ☐☐ year

If not sent, indicate reason : ....

....

....

Height of : calibration signal (1 mV) ☐☐ (mm)

RaVL ☐☐ (mm)

SV_1 ☐☐ (mm)

RV_5 ☐☐ (mm)

Atrial fibrillation ☐ 1 = yes, 2 = no, 9 = unknown

OPTIONAL EXAMINATIONS

Will the patient participate in the following side-projects ?

1. Quality of live assessment* ☐
2. 24-h BP recordings* ☐
3. Storage of serum* ☐
4. Multiple infarction dementia* ☐

1 = yes,
2 = no,
9 = unknown

Were the following examinations performed ?

1. Chest X-ray* ☐ 1 = yes, 2 = no, 9 = unknown

cardiac diameter ☐☐☐ (mm)

thoracic diameter ☐☐☐ (mm)

2. Intravenous pyelography* ☐
1 = normal,
2 = abnormal,
8 = not performed,
9 = unknown

If abnormal, specify : ....

....

....

3. Renal angiography* ☐
1 = normal,
2 = abnormal,
8 = not performed,
9 = unknown

If abnormal, specify : ....

....

....

DECISIONS & COMMENTS

Does the patient continue placebo run-in period ?

☐ 1 = yes, 2 = no, 9 = unknown

If no, specify reason :

....

....

....

Comments :

....

....

....

*Optional ☐ for C.O. ORIGINAL

SYST-EUR

CHECKLIST

IDENTIFICATION & REGISTRATION (1)

Date of visit : ☐☐ ☐☐ ☐☐
day month year

PATIENT	CENTRE
Name of initials : (2)	Centre name : (2) ....
Date of birth : ☐☐ ☐☐ ☐☐ (day month year)	Name of doctor : (2) ....

A. INCLUSION CRITERIA (3)

	Yes	No
1. 60 years or older	☐	☐
2. BP during run-in period at 3 visits combined (= average of 3 x 2 readings)		
— SBP_{sit} : 160-219 mmHg	☐	☐
— DBP_{sit} : ≦ 94 mmHg	☐	☐
— SBP_{stand} : ≧ 140 mmHg	☐	☐
3. Patient willing to cooperate and regular follow-up is possible	☐	☐

for C.O. only

Gender	☐
Complications	☐
Stratum	☐
SBP_{sit}	☐☐☐
DBP_{sit}	☐☐☐
SBP_{stand}	☐☐☐
Supervisor	☐☐☐☐

☐☐☐ ☐ ☐☐☐ ☐☐

B. EXCLUSION CRITERIA (3)

	Yes	No
**1. Certain causes of BP elevation**		
a. Specific cause of SBP elevation, such as hyperthyroidism	☐	☐
b. Conditions which will be corrected by surgery	☐	☐
c. Severe aortic insufficiency, A-V fistula	☐	☐
**2. Certain complications of hypertension**		
a. Presence at recruitment of		
— vascular retinopathy grade III or IV	☐	☐
— overt congestive heart failure	☐	☐
— dissecting aneurysm	☐	☐
— severe renal failure (serum creatinine 180 µmol/L or more)	☐	☐
b. History of		
— repeated severe nose bleeding due to hypertension	☐	☐
— cerebral or subarachnoid haemorrhage	☐	☐
— myocardial infarction < 1 year before randomization	☐	☐
**3. Other diseases**		
— dementia	☐	☐
— hepatic dysfunction	☐	☐
— malignant neoplasm	☐	☐
— diabetes mellitus with frequent insulin adjustments	☐	☐
— patient permanently bed-ridden	☐	☐
— need for drugs with BP lowering activity	☐	☐
**4. Poor collaboration**	☐	☐

acco D1900C—1989

(1) Please complete the checklist together with the report of the third visit during the placebo run-in period and send both together to the Coordinating Office.
(2) Please print.
(3) Tick correct answer.

ORIGINAL

SYST-EUR

SHORT REPORT DURING DOUBLE-BLIND PERIOD

IDENTIFICATION & REGISTRATION (1)

Syst-Eur patient number : ☐☐☐ ☐ ☐☐☐ ☐☐

Date of visit : ☐☐ day ☐☐ month ☐☐ year

Patient	Centre
Name or initials : (2)	Centre name : ....
Date of birth : ☐☐ day ☐☐ month ☐☐ year	Name of doctor : (2) ....

INFORMATION ON TREATMENT

Study medication at moment of visit

	Hypotensor A	Hypotensor B	Hypotensor C
Dose ? (tablets/day)	☐.☐	☐.☐	☐.☐
Dose reduction or interruption since last visit ?<br>2 = no, 3 = yes, dose reduction<br>4 = yes, interruption,<br>8 = not applicable, 9 = unknown	☐	☐	☐
If yes, reason for dose reduction or interruption	........<br>........<br>........	........<br>........<br>........	........<br>........<br>........
	☐ ☐☐☐.☐<br>☐ ☐☐☐.☐<br>☐ ☐☐☐.☐	☐ ☐☐☐.☐<br>☐ ☐☐☐.☐<br>☐ ☐☐☐.☐	☐ ☐☐☐.☐<br>☐ ☐☐☐.☐<br>☐ ☐☐☐.☐

Any other drugs (e.g. aspirin, laxatives)

Drug 1

Name : ....

☐☐☐ / ☐☐☐☐☐

Drug 2

Name : ....

☐☐☐ / ☐☐☐☐☐

Drug 3

Name : ....

☐☐☐ / ☐☐☐☐☐

Drug 4

Name : ....

☐☐☐ / ☐☐☐☐☐

Drug 5

Name : ....

☐☐☐ / ☐☐☐☐☐

Drug 6

Name : ....

☐☐☐ / ☐☐☐☐☐

acco D781A—1988

1. **To be completed at quarterly intervals during double-blind treatment.**
   If a patient leaves the double-blind part of the study, a special form "Report at the End of the Double-Blind Period" should be completed.
2. **Please print.**

☐ for C.O.

ORIGINAL

HISTORY

Did patient have any complaints since last visit ?

☐ 1 = yes, 2 = no, 9 = unknown

If yes, specify :

1 .... ☐ ☐☐☐.☐

2 .... ☐ ☐☐☐.☐

3 .... ☐ ☐☐☐.☐

4 .... ☐ ☐☐☐.☐

5 .... ☐ ☐☐☐.☐

PHYSICAL EXAMINATION

Blood pressure (mmHg)		supine	sitting(5min)	standing(2min)
1	SBP	☐☐☐	☐☐☐	☐☐☐
	DBP (IV)*	☐☐☐	☐☐☐	☐☐☐
	DBP (V)	☐☐☐	☐☐☐	☐☐☐
2	SBP	☐☐☐	☐☐☐	☐☐☐
	DBP (IV)*	☐☐☐	☐☐☐	☐☐☐
	DBP (V)	☐☐☐	☐☐☐	☐☐☐

Sitting pulse rate (bpm) ☐☐☐

Other physical signs ? ☐ 1 = yes, 2 = no

If yes, specify :

1 .... ☐ ☐☐☐.☐

2 .... ☐ ☐☐☐.☐

3 .... ☐ ☐☐☐.☐

4 .... ☐ ☐☐☐.☐

5 .... ☐ ☐☐☐.☐

BLOOD TESTS**

WBC (10^9 cells/L) ☐☐.☐

K (mmol/L) ☐.☐☐

Creatinine (μmol/L) ☐☐☐.☐

DISEASES & FINDINGS

New diseases & findings since last visit ?
(including dementia)

1 .... ☐ ☐☐☐.☐

2 .... ☐ ☐☐☐.☐

3 .... ☐ ☐☐☐.☐

4 .... ☐ ☐☐☐.☐

5 .... ☐ ☐☐☐.☐

Previous diseases & findings still active ?
(including dementia)

1 .... ☐ ☐☐☐.☐

2 .... ☐ ☐☐☐.☐

3 .... ☐ ☐☐☐.☐

4 .... ☐ ☐☐☐.☐

5 .... ☐ ☐☐☐.☐

DECISIONS & COMMENTS

Does the patient continue double-blind study ?(1)

☐ 1 = yes, 2 = no, 9 = unknown

If no, specify reason :

....

....

....

☐ ☐☐☐.☐/☐ ☐☐☐.☐/☐ ☐☐☐.☐

Comments :

....

....

....

☐ ☐☐☐.☐/☐ ☐☐☐.☐/☐ ☐☐☐.☐

* Optional

** Only compulsory during first year of ACE inhibition (otherwise optional)

(1) If a patient leaves the double-blind part of the study, a special form "Report at the End of the Double-Blind Period" should be completed.

☐ for C.O.

ORIGINAL

SYST-EUR

EXTENSIVE REPORT DURING DOUBLE-BLIND PERIOD

IDENTIFICATION & REGISTRATION (1)

Syst-Eur patient number : ☐☐☐ ☐ ☐☐☐ ☐☐

Date of visit : ☐☐ day ☐☐ month ☐☐ year

**Patient**	**Centre**
**Name or initials : (2)**	**Centre name :** ....
**Date of birth :** ☐☐ day ☐☐ month ☐☐ year	**Name of doctor : (2)** ....

INFORMATION ON TREATMENT

Study medication at moment of visit

	Hypotensor A	Hypotensor B	Hypotensor C
Dose ? (tablets/day)	☐.☐	☐.☐	☐.☐
Dose reduction or interruption since last visit ?<br>2 = no, 3 = yes, dose reduction<br>4 = yes, interruption,<br>8 = not applicable, 9 = unknown	☐	☐	☐
If yes, reason for dose reduction or interruption	........................<br>........................<br>........................	........................<br>........................<br>........................	........................<br>........................<br>........................
	☐ ☐☐☐.☐<br>☐ ☐☐☐.☐<br>☐ ☐☐☐.☐	☐ ☐☐☐.☐<br>☐ ☐☐☐.☐<br>☐ ☐☐☐.☐	☐ ☐☐☐.☐<br>☐ ☐☐☐.☐<br>☐ ☐☐☐.☐

Any other drugs (e.g. aspirin, laxatives)

Drug 1

Name : ....

☐☐☐ / ☐☐☐☐☐

Drug 2

Name : ....

☐☐☐ / ☐☐☐☐☐

Drug 3

Name : ....

☐☐☐ / ☐☐☐☐☐

Drug 4

Name : ....

☐☐☐ / ☐☐☐☐☐

Drug 5

Name : ....

☐☐☐ / ☐☐☐☐☐

Drug 6

Name : ....

☐☐☐ / ☐☐☐☐☐

acco D7818—198

1. **To be completed at yearly intervals during double-blind treatment.**
   If a patient leaves the double-blind part of the study, a special form "Report at the End of the Double-Blind Period" should be completed.
2. **Please print.**

☐ for C.O.

ORIGINAL

SMOKING & DRINKING HABITS

Is patient smoking now ? ☐ 1 = yes, 2 = no, 9 = unknown

If yes, give consumption per day of :

☐☐ cigarettes ☐☐ cigars ☐☐ pipes

Does the patient consume alcohol now ? ☐

1 = yes, 2 = no, 9 = unknown

If yes, how many glasses per week of :

☐☐☐ beer ☐☐☐ wine

☐☐☐ spirits ☐☐☐ apéritif or fortified wine

ACTIVITIES OF DAILY LIVING (ADL)

if "yes" score = 1 if "no" score = 0

Can the patient bath alone ? ☐

Can the patient dress alone ? ☐

Can the patient go to the toilet alone ? ☐

Can the patient move in and out bed and chair alone ? ☐

Is the patient entirely self-controlled for urination and defaecation ? ☐

Can the patient eat alone ? ☐

Total ADL score (sum of 6 scores) : ☐

HISTORY

Did patient have any complaints since last visit ?

☐ 1 = yes, 2 = no, 9 = unknown

If yes, specify :

1 .... ☐ ☐☐☐.☐

2 .... ☐ ☐☐☐.☐

3 .... ☐ ☐☐☐.☐

4 .... ☐ ☐☐☐.☐

5 .... ☐ ☐☐☐.☐

PHYSICAL EXAMINATION

Blood pressure (mmHg)

		supine	sitting(5min)	standing(2min)
1	SBP	☐☐☐	☐☐☐	☐☐☐
	DBP (IV)*	☐☐☐	☐☐☐	☐☐☐
	DBP (V)	☐☐☐	☐☐☐	☐☐☐
2	SBP	☐☐☐	☐☐☐	☐☐☐
	DBP (IV)*	☐☐☐	☐☐☐	☐☐☐
	DBP (V)	☐☐☐	☐☐☐	☐☐☐

Sitting pulse rate (bpm) ☐☐☐

Weight (kg) ☐☐☐.☐

Fundoscopy **Right eye** ☐ **Left eye** ☐

(stage Keith-Wagener)

1 = grade I, 2 = grade II, 3 = grade III, 4 = papilloedema, 5 = lens opacity, 8 = normal, 9 = unknown

Other physical signs ? ☐ 1 = yes, 2 = no

If yes, specify :

1 .... ☐ ☐☐☐.☐

2 .... ☐ ☐☐☐.☐

3 .... ☐ ☐☐☐.☐

4 .... ☐ ☐☐☐.☐

5 .... ☐ ☐☐☐.☐

URINE TESTS

Proteinuria ☐
Glucosuria ☐
(1 = positive, 2 = traces, 3 = negative, 9 = unknown)

White blood cells ☐
Red blood cells ☐
Granular casts ☐
(1 = many (≧ 10 field), 2 = rare, 3 = none, 9 = unknown)

Was urine sample sent for evaluation of compliance ?

— If yes, specify date : ☐☐ day ☐☐ month ☐☐ year

— If not, indicate reason, why not : ....

.... ☐☐

* Optional ☐ for C.O. ORIGINAL

BLOOD TESTS

Was patient fasting ? ☐ 1 = yes, 2 = no, 9 = unknown

Haematocrit (%) ☐☐.☐

Haemoglobin (mmol/L) ☐☐.☐

WBC (10^9 cells/L) ☐☐.☐

RBC (10^{12}cells/L) ☐.☐☐

Creatinine (μmol/L) ☐☐☐.☐

Uric acid (mmol/L) ☐.☐☐

Na (mmol/L) ☐☐☐.☐

K (mmol/L) ☐.☐☐

Glucose (mmol/L) ☐☐.☐

Cholesterol :
— total (mmol/L) ☐☐.☐
— HDL (mmol/L) ☐☐.☐

Triglycerides (g/L) ☐☐.☐

Alk. phosph. (U/L) ☐☐☐

SGOT (U/L) ☐☐☐

SGPT (U/L) ☐☐☐

gGT (U/L) ☐☐☐

Insulin (U/ml)* ☐☐☐

ELECTROCARDIOGRAM

ECG normal ? ☐ 1 = yes, 2 = no, 8 = not performed, 9 = unknown

If abnormal, specify : ....

....

....

☐☐☐☐☐ ☐☐☐☐☐ ☐☐☐☐☐

Original sent to C.O. on ☐☐ day ☐☐ month ☐☐ year

If not sent, indicate reason : ....

....

.... ☐☐

Height of : calibration signal (1 mV) ☐☐ (mm)

RaVL ☐☐ (mm)

SV_1 ☐☐ (mm)

RV_5 ☐☐ (mm)

Atrial fibrillation ☐ 1 = yes, 2 = no, 9 = unknown

DISEASES & FINDINGS

New diseases & findings since last visit ?
(including dementia)

1 ....
☐ ☐☐☐.☐

2 ....
☐ ☐☐☐.☐

3 ....
☐ ☐☐☐.☐

4 ....
☐ ☐☐☐.☐

5 ....
☐ ☐☐☐.☐

Previous diseases & findings still active ?
(including dementia)

1 ....
☐ ☐☐☐.☐

2 ....
☐ ☐☐☐.☐

3 ....
☐ ☐☐☐.☐

4 ....
☐ ☐☐☐.☐

5 ....
☐ ☐☐☐.☐

OPTIONAL EXAMINATIONS

Did the patient participate in one of the following side-projects ?

1. Quality of live assessment* ☐
2. 24-h BP recordings* ☐
3. Storage of serum* ☐
4. Multiple infarction dementia* ☐

1 = yes,
2 = no,
9 = unknown

DECISIONS & COMMENTS

Does the patient continue double-blind study ?(1) ☐ 1 = yes, 2 = no, 9 = unknown

If no, specify reason :

....

....

....

☐ ☐☐☐.☐/☐ ☐☐☐.☐/☐ ☐☐☐.☐

Comments :

....

....

....

☐ ☐☐☐.☐/☐ ☐☐☐.☐/☐ ☐☐☐.☐

(1) If a patient leaves the double-blind part of the study, a special form "report at the End of the Double-Blind Period" should be completed.

*Optional ☐ for C.O. **ORIGINAL**

SYST-EUR

REPORT AT THE END OF THE DOUBLE-BLIND PERIOD

IDENTIFICATION & REGISTRATION (1)

Syst-Eur patient number : ☐☐☐ ☐ ☐☐☐ ☐☐

Date of report : ☐☐ ☐☐ ☐☐
day month year

Patient

Name or initials : (2)

....

Date of birth :
☐☐ ☐☐ ☐☐
day month year

Centre

Centre name :

....

Name of doctor : (2)

....

DATE

Specify date of major fatal or non-fatal event, date of patient withdrawal, date on which patient defected from study or date of missed appointment :

☐☐ ☐☐ ☐☐
day month year

REASON

Specify reason why double-blind treatment was stopped:

Death ☐ 1 = yes, 2 = no

If yes, cause of death :

Immediate cause : ....

....
☐ ☐☐☐.☐

Underlying cause(s) :

1. ....
☐ ☐☐☐.☐

2. ....
☐ ☐☐☐.☐

Contributory cause(s) :

1. ....
☐ ☐☐☐.☐

2. ....
☐ ☐☐☐.☐

If yes, was autopsy performed, ☐
and
was protocol sent to C.O. ? ☐

1 = yes,
2 = no,
9 = unknown

Non-fatal events

1 = yes
2 = no

— **Cerebrovascular accident (excluding TIA)**
> cerebral haemorrhage ☐
> subarachnoid haemorrhage ☐
> thrombosis ☐

1 = yes
2 = no

> embolism ☐

— **Retinal lesions**
> exudates or haemorrhage ☐
> papilloedema ☐

— **Myocardial infarction** ☐

— **Congestive heart failure,** requiring diuretics or vasodilators or any antihypertensive drug ☐

— **Dissecting aneurysm** ☐

— **Increase in serum creatinine** by 100 % or to 360 μmol/L (2mg/dl) at 2 consecutive visits ☐

Withdrawal

— **Systolic or diastolic blood pressure too high** ☐

— **Prescription of antihypertensive drugs (≧ 3 months)** ☐

If yes, specify why :

1. ....
☐ ☐☐☐.☐

2. ....
☐ ☐☐☐.☐

— **Code broken** ☐

If yes, specify why :

1. ....
☐ ☐☐☐.☐

2. ....
☐ ☐☐☐.☐

(1) To be completed at end of double-blind period.
(2) Please print.

☐ for C.O.

acco D781C1—1988

ORIGINAL

1 = yes
2 = no

— Interfering disease(s) or adverse drug effect(s)

If yes, specify :

1. ....

2. ....

— Patient followed for 7 years on double-blind medication

— End of whole trial

Defector

Patient refuses :

— to attend clinic

If yes, specify why :

1. ....

2. ....

1 = yes
2 = no

— to take any double-blind medication

If yes, specify why :

1. ....

2. ....

Other reason(s)

If yes, specify :

1. ....

2. ....

FOLLOW-UP

Has the patient been examined at clinic ?

1 = yes, 2 = no, 9 = unknown

If yes, please complete the following sections on pages 2 through 5.

INFORMATION ON TREATMENT

Study medication at moment of visit

	Hypotensor A	Hypotensor B	Hypotensor C
Dose ? (tablets/day)			
Dose reduction or interruption since last visit ?<br>2 = no, 3 = yes, dose reduction<br>4 = yes, interruption,<br>8 = not applicable, 9 = unknown			
If yes, reason for dose reduction or interruption	....	....	....

for C.O.

ORIGINAL

Any other drugs (e.g. aspirin, laxatives)

Drug 1

Name : .... ☐☐☐ / ☐☐☐☐☐

Drug 2

Name : .... ☐☐☐ / ☐☐☐☐☐

Drug 3

Name : .... ☐☐☐ / ☐☐☐☐☐

Drug 4

Name : .... ☐☐☐ / ☐☐☐☐☐

Drug 5

Name : .... ☐☐☐ / ☐☐☐☐☐

Drug 6

Name : .... ☐☐☐ / ☐☐☐☐☐

HISTORY

Did patient have any complaints since last visit ?

☐ 1 = yes, 2 = no, 9 = unknown

If yes, specify :

1 .... ☐ ☐☐☐.☐

2 .... ☐ ☐☐☐.☐

3 .... ☐ ☐☐☐.☐

4 .... ☐ ☐☐☐.☐

5 .... ☐ ☐☐☐.☐

PHYSICAL EXAMINATION

Blood pressure (mmHg)

		supine	sitting(5min)	standing(2min)
1	SBP	☐☐☐	☐☐☐	☐☐☐
	DBP (IV)*	☐☐☐	☐☐☐	☐☐☐
	DBP (V)	☐☐☐	☐☐☐	☐☐☐
2	SBP	☐☐☐	☐☐☐	☐☐☐
	DBP (IV)*	☐☐☐	☐☐☐	☐☐☐
	DBP (V)	☐☐☐	☐☐☐	☐☐☐

Sitting pulse rate (bpm) ☐☐☐

Weight (kg) ☐☐☐.☐

Fundoscopy (stage Keith-Wagener) — **Right eye** ☐ **Left eye** ☐

1 = grade I, 2 = grade II, 3 = grade III, 4 = papilloedema, 5 = lens opacity, 8 = normal, 9 = unknown

Other physical signs ? ☐ 1 = yes, 2 = no

If yes, specify :

1 .... ☐ ☐☐☐.☐

2 .... ☐ ☐☐☐.☐

3 .... ☐ ☐☐☐.☐

4 .... ☐ ☐☐☐.☐

5 .... ☐ ☐☐☐.☐

*Optional ☐ for C.O. ORIGINAL

BLOOD TESTS

Was patient fasting ? ☐ 1 = yes, 2 = no, 9 = unknown

Haematocrit (%) ☐☐.☐

Haemoglobin (mmol/L) ☐☐.☐

WBC (10^9 cells/L) ☐☐.☐

RBC (10^{12}cells/L) ☐.☐☐

Creatinine (μmol/L) ☐☐☐.☐

Uric acid (mmol/L) ☐.☐☐

Na (mmol/L) ☐☐☐.☐

K (mmol/L) ☐.☐☐

Glucose (mmol/L) ☐☐.☐

Cholesterol :
— total (mmol/L) ☐☐.☐
— HDL (mmol/L) ☐☐.☐

Triglycerides (g/L) ☐☐.☐

Alk. phosph. (U/L) ☐☐☐

SGOT (U/L) ☐☐☐

SGPT (U/L) ☐☐☐

gGT (U/L) ☐☐☐

Insulin (U/ml)* ☐☐☐

ELECTROCARDIOGRAM

ECG normal ? ☐ 1 = yes, 2 = no, 8 = not performed, 9 = unknown

If abnormal, specify : ....

....

....

☐☐☐☐☐ ☐☐☐☐☐ ☐☐☐☐☐

Original sent to C.O. on ☐☐ day ☐☐ month ☐☐ year

If not sent, indicate reason : ....

....

.... ☐☐

Height of : calibration signal (1mv) ☐☐ (mm)

RaVL ☐☐ (mm)

SV_1 ☐☐ (mm)

RV_5 ☐☐ (mm)

Atrial fibrillation ☐ 1 = yes, 2 = no, 9 = unknown

URINE TESTS

Proteinuria ☐
Glucosuria ☐
(1 = positive, 2 = traces, 3 = negative, 9 = unknown)

White blood cells ☐
Red blood cells ☐
Granular casts ☐
(1 = many (≧ 10 field), 2 = rare, 3 = none, 9 = unknown)

Was urine sample sent for evaluation of compliance ?

— If yes, specify date : ☐☐ day ☐☐ month ☐☐ year

— If not, indicate reason, why not : ....

.... ☐☐

ACTIVITIES OF DAILY LIVING (ADL)

if "yes" score = 1 if "no" score = 0

Can the patient bath alone ? ☐

Can the patient dress alone ? ☐

Can the patient go to the toilet alone ? ☐

Can the patient move in and out bed and chair alone ? ☐

Is the patient entirely self-controlled for urination and defaecation ? ☐

Can the patient eat alone ? ☐

Total ADL score (sum of 6 scores) : ☐

* Optional ☐ for C.O. ORIGINAL

SMOKING & DRINKING HABITS

Is patient smoking now ? ☐ 1 = yes, 2 = no, 9 = unknown

If yes, give consumption per day of :

☐☐ cigarettes ☐☐ cigars ☐☐ pipes

Does the patient consume alcohol now ? ☐ 1 = yes, 2 = no, 9 = unknown

If yes, how many glasses per week of :

☐☐☐ beer ☐☐☐ wine

☐☐☐ spirits ☐☐☐ apéritif or fortified wine

DISEASES & FINDINGS

New diseases & findings since last visit ?
(including dementia)

1 .... ☐ ☐☐☐.☐

2 .... ☐ ☐☐☐.☐

3 .... ☐ ☐☐☐.☐

4 .... ☐ ☐☐☐.☐

5 .... ☐ ☐☐☐.☐

evious diseases & findings still active ?
(including dementia)

1 .... ☐ ☐☐☐.☐

2 .... ☐ ☐☐☐.☐

3 .... ☐ ☐☐☐.☐

4 .... ☐ ☐☐☐.☐

5 .... ☐ ☐☐☐.☐

DECISIONS & COMMENTS

Does the patient continue supervised open follow-up ?

☐ 1 = yes, 2 = no, 9 = unknown

If no, specify reason :

....

....

....

☐ ☐☐☐.☐/☐ ☐☐☐.☐/☐ ☐☐☐.☐

If only non-supervised follow-up is possible, please indicate how information will be collected in the future :

— via General Practitioner ☐

— via other hospital ☐

— via population register ☐

— via Office for Vital Statistics ☐

— other ☐

1 = yes, 2 = no, 9 = unknown

Please, specify name(s), addresse(s) & telephone number(s) of source(s) of information :

....

....

....

....

....

....

Comments :

....

....

....

☐ ☐☐☐.☐/☐ ☐☐☐.☐/☐ ☐☐☐.☐

☐ for C.O.

ORIGINAL

SYST-EUR

SHORT REPORT DURING SUPERVISED OPEN FOLLOW-UP

IDENTIFICATION & REGISTRATION (1)

Syst-Eur patient number : ☐☐☐ ☐ ☐☐☐ ☐☐

Date of visit : ☐☐ day ☐☐ month ☐☐ year

Patient

Name or initials : (2)

....

Date of birth :

☐☐ day ☐☐ month ☐☐ year

Centre

Centre name :

....

Name of doctor : (2)

....

INFORMATION ON TREATMENT

Drug 1

Name : .... ☐☐☐ / ☐☐☐☐☐

Drug 2

Name : .... ☐☐☐ / ☐☐☐☐☐

Drug 3

Name : .... ☐☐☐ / ☐☐☐☐☐

Drug 4

Name : .... ☐☐☐ / ☐☐☐☐☐

Drug 5

Name : .... ☐☐☐ / ☐☐☐☐☐

Drug 6

Name : .... ☐☐☐ / ☐☐☐☐☐

HISTORY

Did patient have any complaints since last visit ?

☐ 1 = yes, 2 = no, 9 = unknown

If yes, specify :

1 .... ☐ ☐☐☐.☐

2 .... ☐ ☐☐☐.☐

3 .... ☐ ☐☐☐.☐

4 .... ☐ ☐☐☐.☐

5 .... ☐ ☐☐☐.☐

acco D781D—1988

(1) **To be completed at quarterly intervals during supervised open follow-up.**
If a patient leaves the supervised open follow-up, a special form "Report at the End of Supervised Open Follow-up" should be completed.

2. Please print.

☐ for C.O.

ORIGINAL

PHYSICAL EXAMINATION

Blood pressure (mmHg)

		supine	sitting(5min)	standing(2min)
1	SBP			
	DBP (IV)*			
	DBP (V)			
2	SBP			
	DBP (IV)*			
	DBP (V)			

Sitting pulse rate (bpm)

Other physical signs ? 1 = yes, 2 = no

If yes, specify :

1 ....

2 ....

3 ....

4 ....

5 ....

BLOOD TESTS**

WBC (10^9 cells/L) .

K (mmol/L) .

Creatinine (μmol/L) .

DISEASES & FINDINGS

New diseases & findings since last visit ?
(including dementia)

1 ....

2 ....

3 ....

4 ....

5 ....

Previous diseases & findings still active ?
(including dementia)

1 ....

2 ....

3 ....

4 ....

5 ....

DECISIONS & COMMENTS

Does the patient continue supervised open follow-up ?(1)

1 = yes, 2 = no, 9 = unknown

If no, specify reason :

....

....

....

Comments :

....

....

....

* Optional

** Only compulsory during first year of ACE inhibition (otherwise optional)

(1) If a patient leaves the supervised open follow-up, a special form "Report at the End of Supervised Open Follow-up" should be completed.

for C.O.

ORIGINAL

SYST-EUR
EXTENSIVE REPORT DURING SUPERVISED OPEN FOLLOW-UP

IDENTIFICATION & REGISTRATION (1)

Syst-Eur patient number : ☐☐☐ ☐ ☐☐☐ ☐☐

Date of visit : ☐☐ day ☐☐ month ☐☐ year

Patient	Centre
**Name or initials :** (2)	**Centre name :** ....
**Date of birth :** ☐☐ day ☐☐ month ☐☐ year	**Name of doctor :** (2) ....

INFORMATION ON TREATMENT

Drug 1

Name : .... ☐☐☐ / ☐☐☐☐☐

Drug 2

Name : .... ☐☐☐ / ☐☐☐☐☐

Drug 3

Name : .... ☐☐☐ / ☐☐☐☐☐

Drug 4

Name : .... ☐☐☐ / ☐☐☐☐☐

Drug 5

Name : .... ☐☐☐ / ☐☐☐☐☐

Drug 6

Name : .... ☐☐☐ / ☐☐☐☐☐

SMOKING & DRINKING HABITS

Is patient smoking now ? ☐ 1 = yes, 2 = no, 9 = unknown

If yes, give consumption per day of :

☐☐ cigarettes ☐☐ cigars ☐☐ pipes

Does the patient consume alcohol now ? ☐ 1 = yes, 2 = no, 3 = unknown

If yes, how many glasses per week of :

☐☐☐ beer ☐☐☐ wine

☐☐☐ spirits ☐☐☐ apéritif or fortified wine

ACTIVITIES OF DAILY LIVING (ADL)

if "yes" score = 1 if "no" score = 0

Can the patient bath alone ? ☐

Can the patient dress alone ? ☐

Can the patient go to the toilet alone ? ☐

Can the patient move in and out bed and chair alone ? ☐

Is the patient entirely self-controlled for urination and defaecation ? ☐

Can the patient eat alone ? ☐

Total ADL score (sum of 6 scores) : ☐

1. **To be completed at yearly intervals during supervised open follow-up.**
   If a patient leaves the supervised open follow-up, a special form "Report at the End of Supervised Open Follow-up" should be completed.
2. **Please print.**

☐ for C.O.

HISTORY

Did patient have any complaints since last visit ?

☐ 1 = yes, 2 = no, 9 = unknown

If yes, specify :

1 .... ☐ ☐☐☐.☐

2 .... ☐ ☐☐☐.☐

3 .... ☐ ☐☐☐.☐

4 .... ☐ ☐☐☐.☐

5 .... ☐ ☐☐☐.☐

PHYSICAL EXAMINATION

Blood pressure (mmHg)

		supine	sitting(5min)	standing(2min)
1	SBP	☐☐☐	☐☐☐	☐☐☐
	DBP (IV)*	☐☐☐	☐☐☐	☐☐☐
	DBP (V)	☐☐☐	☐☐☐	☐☐☐
2	SBP	☐☐☐	☐☐☐	☐☐☐
	DBP (IV)*	☐☐☐	☐☐☐	☐☐☐
	DBP (V)	☐☐☐	☐☐☐	☐☐☐

Sitting pulse rate (bpm) ☐☐☐

eight (kg) ☐☐☐.☐

Fundoscopy — **Right eye** ☐ — **Left eye** ☐

(stage Keith-Wagener)

1 = grade I, 2 = grade II, 3 = grade III, 4 = papilloedema,
5 = lens opacity, 8 = normal, 9 = unknown

Other physical signs ? ☐ 1 = yes, 2 = no

If yes, specify :

1 .... ☐ ☐☐☐.☐

2 .... ☐ ☐☐☐.☐

3 .... ☐ ☐☐☐.☐

4 .... ☐ ☐☐☐.☐

5 .... ☐ ☐☐☐.☐

URINE TESTS

Proteinuria ☐

Glucosuria ☐

1 = positive,
2 = traces,
3 = negative,
9 = unknown

White blood cells ☐

Red blood cells ☐

Granular casts ☐

1 = many (≧ 10 field),
2 = rare,
3 = none,
9 = unknown

BLOOD TESTS

Was patient fasting ? ☐ 1 = yes, 2 = no, 9 = unknown

Haematocrit (%)	☐☐.☐
Haemoglobin (mmol/L)	☐☐.☐
WBC (10^9 cells/L)	☐☐.☐
RBC (10^{12}cells/L)	☐.☐☐
Creatinine (µmol/L)	☐☐☐.☐
Uric acid (mmol/L)	☐.☐☐
Na (mmol/L)	☐☐☐.☐
K (mmol/L)	☐.☐☐
Glucose (mmol/L)	☐☐.☐
Cholesterol : — total (mmol/L)	☐☐.☐
— HDL (mmol/L)	☐☐.☐
Triglycerides (g/L)	☐☐.☐
Alk. phosph. (U/L)	☐☐☐
SGOT (U/L)	☐☐☐
SGPT (U/L)	☐☐☐
gGT (U/L)	☐☐☐
Insulin (U/ml)*	☐☐☐

*Optional ☐ for C.O. ORIGINAL

ELECTROCARDIOGRAM

ECG normal ? ☐ 1 = yes, 2 = no, 8 = not performed, 9 = unknown

If abnormal, specify : ....

....

....

Original sent to C.O. on ☐☐ day ☐☐ month ☐☐ year

If not sent, indicate reason : ....

....

.... ☐☐

Height of : calibration signal (1mV) ☐☐ (mm)

RaVL ☐☐ (mm)

SV1 ☐☐ (mm)

RV5 ☐☐ (mm)

Atrial fibrillation ☐ 1 = yes, 2 = no, 9 = unknown

DISEASES & FINDINGS

New diseases & findings since last visit ?
(including dementia)

....

2 ....

3 ....

4 ....

5 ....

Previous diseases & findings still active ? (including dementia)

1 ....

2 ....

3 ....

4 ....

5 ....

OPTIONAL EXAMINATIONS

Did the patient participate in one of the following side-projects ?

1. Quality of live assessment* ☐
2. 24-h BP recordings* ☐
3. Storage of serum* ☐
4. Multiple infarction dementia* ☐

1 = yes,
2 = no,
9 = unknown

DECISIONS & COMMENTS

Does the patient continue supervised open follow-up ?(1) ☐ 1 = yes, 2 = no, 9 = unknown

If no, specify reason :

....

....

....

Comments :

....

....

....

(1) If a patient leaves the supervised open follow-up, a special from "Report at the End of Supervised Open Follow-up" should be completed.

* Optional ☐ for C.O. ORIGINAL

SYST-EUR

REPORT AT THE END OF SUPERVISED OPEN FOLLOW-UP

IDENTIFICATION & REGISTRATION (1)

Syst-Eur patient number : ☐☐☐ ☐ ☐☐☐ ☐☐

Date of report : ☐☐ day ☐☐ month ☐☐ year

Patient

Name or initials : (2)

....

Date of birth :

☐☐ day ☐☐ month ☐☐ year

Centre

Centre name :

....

Name of doctor : (2)

....

DATE

Specify date of major fatal or non-fatal event, date of patient withdrawal, date on which patient defected from study or date of missed appointment :

☐☐ day ☐☐ month ☐☐ year

REASON

Specify reason why supervised open follow-up was ended :

Death ☐ 1 = yes, 2 = no

If yes, cause of death :

Immediate cause : ....

....

Underlying cause(s) : ☐ ☐☐☐.☐

1. .... ☐ ☐☐☐.☐

2. .... ☐ ☐☐☐.☐

Contributory cause(s) :

1. .... ☐ ☐☐☐.☐

2. .... ☐ ☐☐☐.☐

If yes, was autopsy performed, ☐

and

was protocol sent to C.O. ? ☐

1 = yes, 2 = no, 9 = unknown

Non-fatal events (1 = yes, 2 = no)

— **Cerebrovascular accident (excluding TIA)**
  > cerebral haemorrhage ☐
  > subarachnoid haemorrhage ☐
  > thrombosis ☐
  > embolism ☐

— **Retinal lesions**
  > exudates or haemorrhage ☐
  > papilloedema ☐

— **Myocardial infarction** ☐

— **Congestive heart failure,** requiring diuretics or vasodilators or any antihypertensive drug ☐

— **Dissecting aneurysm** ☐

— **Increase in serum creatinine** by 100 % or to 360 μmol/L (2mg/dl) at 2 consecutive visits ☐

Withdrawal

— **Interfering disease(s) or adverse drug effect(s)** ☐

If yes, specify :

1. .... ☐ ☐☐☐.☐

2. .... ☐ ☐☐☐.☐

— **End of whole trial** ☐

Defector

Patient refuses to attend clinic : ☐

If yes, specify why :

1. .... ☐ ☐☐☐.☐

2. .... ☐ ☐☐☐.☐

Other reason(s) ☐

If yes, specify :

1. .... ☐ ☐☐☐.☐

2. .... ☐ ☐☐☐.☐

FOLLOW-UP

Has the patient been examined at clinic ? ☐ 1 = yes, 2 = no, 9 = unknown

If yes, please complete the following sections on pages 2 through 4.

(1) To be completed at end of supervised open follow-up.
(2) Please print.

☐ for C.O.

INFORMATION ON TREATMENT

Drug 1

Name : .... ☐☐☐ / ☐☐☐☐☐

Drug 2

Name : .... ☐☐☐ / ☐☐☐☐☐

Drug 3

Name : .... ☐☐☐ / ☐☐☐☐☐

Drug 4

Name : .... ☐☐☐ / ☐☐☐☐☐

Drug 5

Name : .... ☐☐☐ / ☐☐☐☐☐

Drug 6

Name : .... ☐☐☐ / ☐☐☐☐☐

SMOKING & DRINKING HABITS

Is patient smoking now ? ☐ 1 = yes, 2 = no, 9 = unknown

If yes, give consumption per day of :

☐☐ cigarettes ☐☐ cigars ☐☐ pipes

Does the patient consume alcohol now ? ☐ 1 = yes, 2 = no, 9 = unknown

If yes, how many glasses per week of :

☐☐☐ beer ☐☐☐ wine

☐☐☐ spirits ☐☐☐ apéritif or fortified wine

ACTIVITIES OF DAILY LIVING (ADL)

if "yes" score = 1 if "no" score = 0

Can the patient bath alone ? ☐

Can the patient dress alone ? ☐

Can the patient go to the toilet alone ? ☐

Can the patient move in and out bed and chair alone ? ☐

Is the patient entirely self-controlled for urination and defaecation ? ☐

Can the patient eat alone ? ☐

Total ADL score (sum of 6 scores) : ☐

HISTORY

Did patient have any complaints since last visit ?

☐ 1 = yes, 2 = no, 9 = unknown

If yes, specify :

1 .... ☐ ☐☐☐.☐

2 .... ☐ ☐☐☐.☐

3 .... ☐ ☐☐☐.☐

4 .... ☐ ☐☐☐.☐

5 .... ☐ ☐☐☐.☐

☐ for C.O. ORIGINAL

PHYSICAL EXAMINATION

Blood pressure (mmHg)

		supine	sitting(5min)	standing(2min)
1	SBP			
	DBP (IV)*			
	DBP (V)			
2	SBP			
	DBP (IV)*			
	DBP (V)			

Sitting pulse rate (bpm)

Weight (kg)

Fundoscopy **Right eye** **Left eye**

(stage Keith-Wagener)

1 = grade I, 2 = grade II, 3 = grade III, 4 = papilloedema,
5 = lens opacity, 8 = normal, 9 = unknown

Other physical signs ? 1 = yes, 2 = no

If yes, specify :

1 ....

2 ....

3 ....

4 ....

5 ....

URINE TESTS

Proteinuria
Glucosuria

1 = positive,
2 = traces,
3 = negative,
9 = unknown

White blood cells
Red blood cells
Granular casts

1 = nany (≥ 10 field),
2 = rare,
3 = none
4 = unknown

BLOOD TESTS

Was patient fasting ? 1 = yes, 2 = no, 9 = unknown

Haematocrit (%)
Haemoglobin (mmol/L)
WBC (10^9 cells/L)
RBC (10^{12}cells/L)
Creatinine (μmol/L)
Uric acid (mmol/L)
Na (mmol/L)
K (mmol/L)
Glucose (mmol/L)
Cholesterol :
— total (mmol/L)
— HDL (mmol/L)
Triglycerides (g/L)
Alk. phosph. (U/L)
SGOT (U/L)
SGPT (U/L)
gGT (U/L)
Insulin (U/ml)*

*Optional

for C.O.

ORIGINAL

ELECTROCARDIOGRAM

ECG normal ? ☐ 1 = yes, 2 = no, 8 = not performed, 9 = unknown

If abnormal, specify : ....

....

....

Original sent to C.O. on ☐☐ day ☐☐ month ☐☐ year

If not sent, indicate reason : ....

....

....

Height of : calibration signal (1mV) ☐☐ (mm)

RaVL ☐☐ (mm)

SV1 ☐☐ (mm)

RV5 ☐☐ (mm)

Atrial fibrillation ☐ 1 = yes, 2 = no, 9 = unknown

DISEASES & FINDINGS

New diseases & findings since last visit ?
(including dementia)

1 ....
2 ....
3 ....
4 ....
5 ....

Previous diseases & findings still active ?
(including dementia)

1 ....
2 ....
3 ....
4 ....
5 ....

DECISIONS & COMMENTS

Please indicate how information will be collected during non-supervised open follow-up ?

— via General Practitioner ☐
— via other hospital ☐
— via population register ☐
— via Office for Vital Statistics ☐
— other ☐

1 = yes,
2 = no,
9 = unknown

Please, specify name(s), addresse(s) & telephone number(s) of source(s) of information :

....
....
....
....
....
....

Comments :

....
....
....

* Optional ☐ for C.O. ORIGINAL

SYST-EUR
ANNUAL REPORT DURING NON-SUPERVISED OPEN FOLLOW-UP

IDENTIFICATION & REGISTRATION (1)

Syst-Eur patient number : ☐☐☐ ☐ ☐☐☐ ☐☐

Date of visit : ☐☐ day ☐☐ month ☐☐ year

Patient

Name or initials : (2)

....

Date of birth :

☐☐ day ☐☐ month ☐☐ year

Centre

Centre name :

....

Name of doctor : (2)

....

VITAL STATUS

Is patient deceased ? ☐ 1 = yes, 2 = no, 9 = unknown

If patient is deceased, please specify :

date of death ☐☐ day ☐☐ month ☐☐ year

Cause(s) of death :

Immediate cause . ...

.... ☐ ☐☐☐.☐

Underlying cause(s) :

1 .... ☐ ☐☐☐.☐

2 .... ☐ ☐☐☐.☐

Contributory cause(s) :

1 .... ☐ ☐☐☐.☐

2 .... ☐ ☐☐☐.☐

If patient is alive, please indicate whether he (she) is currently taking antihypertensive drugs :

☐ 1 = yes, 2 = no, 9 = unknown

DISEASES & FINDINGS

New diseases & findings since last report ?
(including dementia)

1 .... ☐ ☐☐☐.☐

2 .... ☐ ☐☐☐.☐

3 .... ☐ ☐☐☐.☐

4 .... ☐ ☐☐☐.☐

5 .... ☐ ☐☐☐.☐

Previous diseases & findings still active ?
(including dementia)

1 .... ☐ ☐☐☐.☐

2 .... ☐ ☐☐☐.☐

3 .... ☐ ☐☐☐.☐

4 .... ☐ ☐☐☐.☐

5 .... ☐ ☐☐☐.☐

COMMENTS

....

....

....

☐ ☐☐☐.☐/☐ ☐☐☐.☐/☐ ☐☐☐.☐

acco D781G—1988

(1) **To be completed at yearly intervals during non-supervised open follow-up, i.e. in patients who do not continue to attend the clinic.**

(2) Please print.

☐ for C.O. ORIGINAL

APPENDIX VII

ACTIVITIES OF DAILY LIVING*

1. Aims of antihypertensive drug treatment

The aim of antihypertensive treatment in the elderly is :

* to reduce cardiovascular morbidity and/or mortality
* while maintening general well-being

Being independent in activities of daily living constitutes an important aspect of general well-being, and can be rated in a simple way.

2. Activities of daily living (ADL)

Several indexes or scales have been proposed to measure independence in activities of daily living. Such scales must have been validated and must be reliable, even if administered by non-specialized clinical investigators in a simplified fashion. The procedure should not be time consuming.

Among all the published and validated scales, Katz's index seems to be the most appropriate for the purpose of SYST-EUR since it provides the simplest and the shortest rating system and it is known to be sensitive to intra-individual changes (for reference see Katz S., Downs T.D., Cash H.R., Grotz R.C., Gerontologist 1970, 1 : 20-30).

---

* Proposal by F. Forette and adopted with slight modifications at the meeting of March 18, 1988.

3. <u>Scoring by the original Katz index</u>

The original Katz index evaluates the dependence or independence on six functions : bathing, dressing, toileting, transfer (mobility), continence and feeding.

The original scoring system is based on the theory of a pattern of disappearance of basic functions (e.g. first bathing, then dressing, and so forth). While this scoring system is useful to investigators interested in the research of the ageing process itself, it makes scoring rather complicated. Moreover, the theory behind the scoring system has not always been verified. Therefore a scoring system is proposed based on 5 functions and 2 grades (dependent or independent).

4. <u>The simplified scoring system for activities of daily living to be used in SYST-EUR</u>

	yes	no
- Can the patient bath alone ?		
- Can the patient dress alone ?		
- Can the patient go to the toilet alone ?		
- Can the patient move in and out bed and chair alone ?		
- Is the patient entirely self-controlled for urination and defaecation ?		
- Can the patient eat alone ?		
Total score		

APPENDIX VIII

ECG-RECORDINGS IN MULTICENTRE CLINICAL TRIALS

Electrocardiographic recordings in multicentre clinical trials, such as SYST-EUR require standardization of ECG equipment, positioning of the electrodes, and procedures of recording and coding.

A. ECG equipment

All ECG recorders, either single channel or multichannel, must meet the following technical specifications : the writing stylus has to be stable in order to give a flat stable line in the neutral position. The width of this line should not exceed 0.5 mm. The paper speed of the machine must be accurate. Baseline oscillation due to AC interference has to be avoided. For each ECG, calibration of each channel has to be done by a stable calibrator. The calibration must be adjusted each time for each channel to reach exactly 10 mm. During calibration filters have to be de-activated.

B. Electrodes

Stainless steel electrodes are recommended for the limb leads and for the chest leads infant size cup electrodes.

Special care has to be taken to position the precordial electrodes, as described by Prineas et al. in the Minnesota Code Manual of Electrocardiographic Findings, 1982 :

---

* Proposal by R. Van Hoof, which remains to be discussed.

"Electrode V_2 : 1) Locate the angle between sternum and second left rib with the index and middle fingers of the right hand; 2) Count down to the fourth intercostal space below it; 3) Locate V_2 in the fourth intercostal space at the left sternal border. Mark V_2 location with a dot (with a good quality felt tip pen).

"Electrode V_1 : Locate electrode V_1 in the fourth intercostal space at the right sternal border. This should be at the same levels as V_2 and immediately to the right of the sternum. Mark V_1 location with a dot.

"Anterior 5th interspace marker (E point) : Identify the fifth intercostal space below V_2 in the manner previously described. Follow this space to the midsternal line and mark this point. This is the "E" point.

"Electrodes V_3 - V_6 : 1) Locate the V_6 electrode position at the same level of the E point in the midaxillary line. Mark this location with a dot. This identifies the horizontal level for V_4 - V_6 electrodes; 2) Using a metric tape, measure the horizontal distance in centimeters from the E point to V_6 to the nearest 0.5 cm. The midpoint distance is the V_4 electrode location. Mark this location; 3) Using a flexible ruler, measure the distance between V_4 and V_6. The midpoint is the location of the V_5 electrode. Place a dot at this site; 4) In a similar manner measure the distance between V_2 and V_4. The midpoint is the location of the V_3 electrode. This site should also be marked."

Only a very small amount of jelly has to be placed under each electrode. Jelly-contact between two adjacent electrodes has to be avoided.

If placement of the precordial electrodes is difficult due to excess hair on the breast, the subject's consent is asked to remove the excess hair at the sites of the electrodes. An other possibility is to fix the precordial electrodes by a broad rubber band, placed around the thorax.

C. Recording procedures

It is important that the same procedures are followed for each ECG, taken in this trial.

An ECG should be recorded not earlier than two hours after a meal, smoking or heavy exercise and, if possible, not earlier than 30 minutes after blood sampling. The ECG-room should have an ambiant temperature of nearly 25°C to avoid muscle tremor. The subject, stripped to the waist, is asked to lie on the recording table with the arms relaxed and to breath quietly and to avoid movements. A subject, who undergoes an ECG for the first time in his life, is reassured that there will be no discomfort.

A calibration mark should be recorded, in each channel. This calibration mark has to be adjusted to exactly 10 mm. Leads recorded with a reduced calibration (because of high voltage) should be preceded by a calibration mark of exactly 5 mm.

The tracings are recorded at a paper speed of 25 mm per second and for each lead, I, II, III, aVR, aVL, aVF, V_1 to V_6, a strip of at least 5 seconds (125 mm) should be obtained. The

paper speed of all ECG-machines should be checked before the start of the trial in each participating centre.

If baseline noise or baseline fluctuations occur during the recording procedure, the procedure should be stopped, the patient, the electrodes and the apparatus checked, and the ECG should be repeated in better conditions.

Each ECG should contain complete patient identification (name, date of birth, study number and date). The leads should be labelled appropriately by the technician. All ECGs should be submitted to the physician in charge of the patient who will also check the validity and the quality of the recordings. Cutting of the ECG has to be avoided and the original ECG has to be sent to the Coordinating Office.

D. <u>Fault detection procedures</u>

(by R.J. Prineas et al., in The Minnesota Code Manual of Electrocardiographic Findings, 1982)

"... If problems with noise or drift are encountered, electrodes should be replaced. The following is a guide for determining which electrodes may be at fault. The underlined electrodes are the predominant determinants of the lead and, therefore, are the usual electrodes affecting a given lead. After adjustment and/or replacement of suspect electrodes, all leads should be tested again for quality.

Lead affected	Possible Faulty Electrode
I	RL, RA, LA
II	RL, RA, LL
III	RL, LA, LL
aVR	RL, RA, LL, LA
aVL	RL, LL, RA, LA
aVF	RL, LL, RA, LA
V_n	RL, LL, RA, LA, V_n

(V_n n = from 1 to 6)

E. Coding procedures

Each ECG will be coded at the Coordinating Office immediately upon arrival by two independent coders. These two coders should be trained and tested before, in order to minimize inter-coder differences. Each ECG which has been differently coded by the two regular coders, should be noted independently by a third coder.

Codes should be recorded on a standard form, especially designed for the trial. The coders can use either the Minnesota Code (R.J. Prineas et al., 1982), or a slight adaptation of the Minnesota Code, currently in use to code the ECGs of the EWPHE-trial (to be published).

Literature

1. R.J. Prineas, R.S. Crow, H. Blackburn : The Minnesota Code Manual of Electrocardiographic Findings - Standards and Procedures for Measurement and Classification. Laboratory of Physiological Hygiene, School of Public Health, University of Minnesota. Ed. : John Wright - PSG Inc - 1982.

2. H.V. Pipberger, R.E. Artsbaecher, A.S. Berson et al. : Recommendations for standardization of leads and of specifications for instruments in electrocardiography and vectocardiography. American Heart Association Committee on Electrocardiography. Circulation 1975; 52 : 11-31.

APPENDIX IX

CLASSIFICATION OF HYPERTENSION

These guidelines are taken from the Report by the WHO Expert Committee (Reference : Arterial Hypertension, Report of a WHO Expert Committee, World Health Organization Technical Report Series 628, World Health Organization, Geneva, 1978, pp. 8-11).

1. Classification according to extent of organ damage : stages of hypertension

The rate of progression of hypertension varies from one individual to another depending on many influences, but the extent of organ involvement corresponds most closely to the level of pressure. Nevertheless, both blood pressure and organ impairment should be evaluated separately, since markedly high pressures, carrying a high risk, may be seen without organ damage and, conversely, organ damage may be present with only moderate elevation of blood pressure.

1.1. Stage I

No objective signs of organic changes are evident.

1.2. Stage II

At least one of the following signs of organ involvement is present :

- Left ventricular hypertrophy on physical examination, chest X-ray, electrocardiography, echocardiography, etc.

- Generalized and focal narrowing of the retinal arteries.

- Proteinuria and/or slight elevation of plasma creatinine concentration.

1.3. Stage III

Both symptoms and signs have appeared as a result of damage to various organs from hypertensive disease. These include :

- Heart : left ventricular failure.

- Brain : cerebral, cerebellar, or brain stem haemorrhage; hypertensive encephalopathy.

- Optic fundi : retinal haemorrhages and exudates with or without papilloedema. These features are pathognomonic of the malignant (accelerated) phase.

Other conditions frequently present in Stage III but less clearly a direct consequence of hypertension include :

- Heart : angina pectoris; myocardial infarction.
- Brain : intracranial arterial thrombosis.
- Vessels : dissecting aneurysm; arterial occlusive disease.
- Kidney : renal failure.

2. Classification by aetiology

2.1. Essential or primary hypertension

This is defined as high blood pressure without evident organic cause.

2.2. Secondary hypertension

This is defined as hypertension with identifiable cause. The possible causes are classified below.

(1) Hypertension due to the administration of drugs.

- Hormonal contraceptives.
- Licorice and carbenoxolone.
- ACTH and corticosteroids.
- Others.

(2) Organic disease.

- Coarctation of the aorta.
- Renal diseases (renal artery stenosis; glomerulonephritis; pyelonephritis; radiation nephritis; renal tuberculosis; renal cysts; hydronephrosis; renal tumours, including renin-secreting tumours; renal failure).
- Diseases of the adrenal cortex (primary hyperaldosteronism; Cushing's syndrome; tumours producing excess of other corticosteroids, e.g., corticosterone and desoxycortone; inborn errors of corticosteroid biosynthesis).
- Diseases of the adrenal medulla (phaeochromocytoma).

APPENDIX X

TRIAL TERMINATION

A. Fletcher

Royal Postgraduate Medical School

The Systeur trial is designed to test the hypothesis that there is no difference in the stroke event rate (fatal and non-fatal) between subjects on active treatment and placebo.

A previous report to the Steering Committee suggested that a total of 15,000 patient years would need to be accumulated to detect a 40 % reduction in an event rate of 20/1000 with a power of 90 % and a significance level of 1 %.

The trial may be stopped early, i.e. before the calculated patients and events have accumulated, because of a differential effect on morbidity or mortality between placebo and active, or because of the difference between placebo and active treatment is so small that continuation of the trial is not justified. We should take account of both these situations although our main concern in this report is with the former, which places a heavier "ethical" responsability on the Steering Committee.

Although statistical results are a major guideline in the decision to stop the trial prematurely, rigid stopping rules

should be avoided. A serious limitation of stopping rules is that they cannot reflect all of the possible outcomes that might arise during the course of the trial, and a more flexible approach is required although the evidence provided by statistical tests must remain of paramount importance. Other considerations influencing the decision relate to the overall risks and benefits, the likelihood that the observed result would be reversed if the trial continued, the additional information and confidence to be obtained by continuing the trial, and, most important, whether there is any bias in event ascertainment between the two groups. Other, and perhaps more controversial areas relate to the consistency of results across subgroups and centres.

In this report we are primarily concerned with the planning of interim analyses to provide statistical reports as the basis for decision making.

Interim Analyses

The following points need to be resolved.

1. How many interim analyses should be done ?

2. When should interim analyses be done ? i.e. should they be time based or event based ?

3. How should the Type 1 error be adjusted to take account of interim analyses ?

4. What is the effect of irregular patient recruitment on the planning of analyses ?

5. What is the effect of a lower than expected event rate in planning interim analyses ?

6. Which statistic will be used to test the differences between the groups ?

7. How many end points will be tested ?

The following discussion will attempt to resolve some of these points with reference to the use of group sequential methods. The common basis of these methods is the provision of boundaries which are the plots of the values of the standardized normal deviate (2) against the number of analyses (Figure 1). The Z values are therefore adjusted to take account of repeated testing. Pocock's and Peto's methods use a constant critical value throughout the study including the end, but with the disadvantage of requiring a large critical value at the end of the study. Peto's method also uses a very large Z value of $\pm$ 3.0.

The O'Brien Fleming approach involves using a very large Z value at the beginning which is relaxed as the trial progresses and more end-points accrue. There are many advantages to this approach; the trial will only stop early if the results are extreme, and the final Z value is approximately the same as if a single test were done.

Most methods involve specifying the total number of analyses in advance with the disadvantage that factors such as slower recruitment, and less end-points may extend the trial and therefore involve further analyses.

Several authors have shown that between 2 and 5 analyses provide the greatest gains in terms of statistical, practical and ethical considerations.

The statistic used to test the difference between the groups at each analysis will be either the Cox Proportional Hazards Model ratio, or the logrank statistic comparing two survival curves. Either of these statistics can be used with group sequential methods to provide monitoring boundaries. For example the Beta-Blocker Heart Attack Trial used the O'Brien Fleming Boundary for the logrank statistic for a 2 sided significance level of 5 % with 7 data reviews (Figure 2). Strictly, the actual figures plotted should be computed in time intervals determined by equal numbers of deaths although the methods are fairly robust to variations in the number of deaths.

We may wish to consider newer procedures, such as the calculation of repeated Confidence Intervals, or a more flexible approach wich does not require the specification of the actual number of analyses in advance.

The present recommendations for decision making allow the Steering Committee to monitor mortality and morbidity during the trial.

1. Calculate boundaries for overall significance levels of 10 %, 5 % and 1 % for total mortality, stroke events (fatal and non-fatal), cardiovascular mortality, cardiac events (fatal and non-fatal) and cardiac mortality.

   The O'Brien Fleming method gives greater weight to later occuring survival differences and is appropriate in a hypertensive trial.

   The Cox Proportional Hazards Model or the logrank are appropriate statistics for the boundaries.

2. Estimate the recruitment rate of patients and the length of time to obtain the calculated sample size in order to decide on the number of analysis including the final one.

3. The criteria for analyses should be event based. However this will mean that different analyses are carried out at different times due to the monitoring of a variety of events. Further consideration needs to be given to this point.

4. The boundaries for a treatment benefit or adverse effect need not be symmetric since they reflect different priorities, for example, the Type 1 error should be very small in deciding to stop the trial for a treatment benefit, but could be larger for an adverse treatment effect.

   This would mean that stopping the trial would be considered if the 1 % boundaries for a treatment effect or the lower

10 % boundaries for an adverse treatment effect are crossed for the following; total mortality, cardiovascular mortality, cardiac mortality, fatal and non-fatal cardiovascular events combined, fatal and non-fatal cerebrovascular events combined and fatal and non-fatal cardiac events combined.

5. Methods for calculating the likelihood of reversal of the data and gain in precision with continuation should be employed.

References

1. Armitage P., McPherson C.K., Rowe B.C.: Repeated significance tests on accumulating data. J. Roy. Statist. Soc. A 1969, 132:235-244.
2. Pocock S.J.: Group sequential methods in the design and analysis of clinical trials. Biometrika 1977, 64:191-199.
3. Peta R., Pike M.C., Armitage P. et al.: Design and analysis of randomized clinical trials requiring prolonged observations of each patient. Br. J. Cancer 1976, 34:585-612.
4. O'Brien P.C., Fleming T.R.: A multiple testing procedure for clinical trials. Biometrics 1979, 35:549-556.

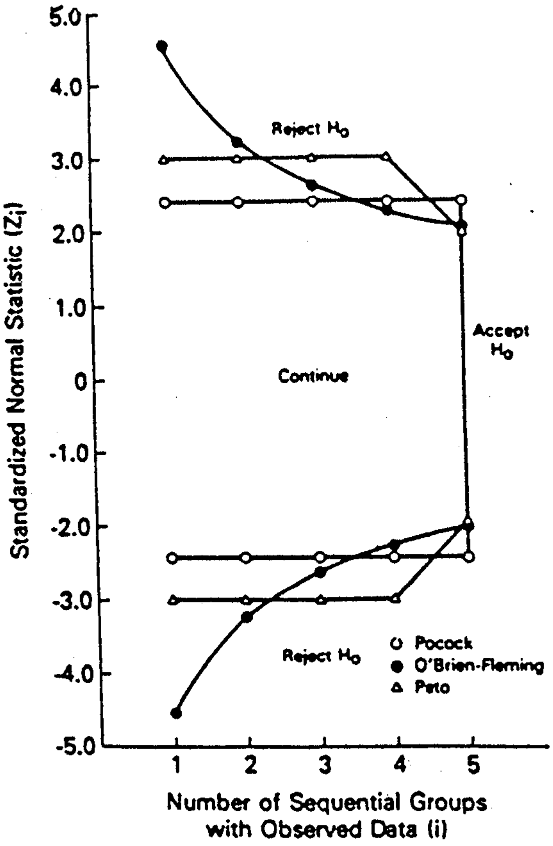

Figure 1'

Figure 2 Beta-Blocker Heart Attack Trial Monitoring Boundary

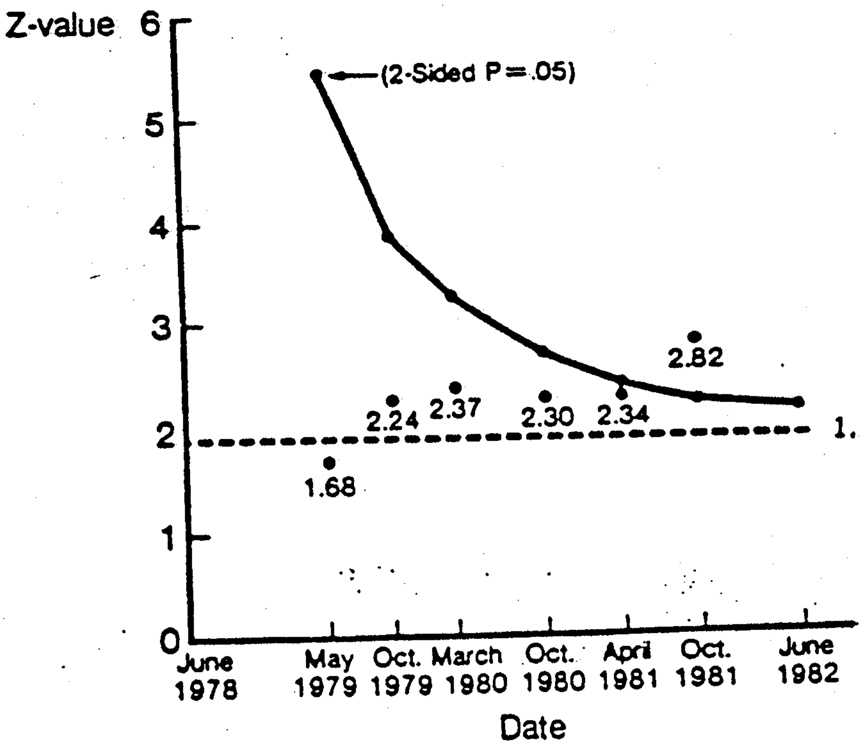

Syst-Eur Monitoring Boundaries

5 interim analyses

α = 0.01 overall

	1	2	3	4	5
Z =	5.21	3.68	3.01	2.61	2.33

α = 0.05 overall

	1	2	3	4	5
Z =	4.38	3.10	2.53	2.19	1.96

α = 0.10 overall

	1	2	3	4	5
Z =	3.67	2.59	2.12	1.83	1.64

PROTECTION OF PRIVACY OF THE SYST-EUR PATIENTS

1. Location of the data

The original patient' forms are kept at the Coordinating Office in Leuven. Copies of the patient forms are retained at the local centres.

The central database, containing the information mentioned in chapter XII of the main protocol, will be stored on the mini-computer at the Coordinating Office. A copy of this database will be kept at the central computer of the University of Leuven. In the central database, patients are identified only by their patient number. Other identification data such as name and date of birth will be stored in a separate database on a personal computer at the Coordinating Office.

2. Persons who have right of access to the data

Only the members of the Coordinating Office have access to the central database and to the individual patient forms. The Ethical Committee and the Monitoring Committee can request data from individual patients through the Coordinating Office. Information must not be disclosed to any other person.

3. Security measures against unauthorized access to the data

a. Data kept at the local centres

The local investigator is responsible for the protection of the data in his/her centre.

b. Data kept at the Coordinating Office

The computer at the Coordinating Office and the central computer at the University of Leuven can be reached through several national and international computer networks. Unauthorized access to the data is prevented by the use of passwords which are only known by the members of the Coordinating Office. Moreover, the identification data of the patients is stored on a separate computer which is not accessible through any network.

Backups will be stored in closed cabinets.

APPENDIX XII : PROPOSAL FOR COMMITTEES

1. Steering Committee:

   - members : a representative of each centre entering a sufficient number of patients (principal investigators)
   - elects its chairman
   - meets at regular intervals
   - is responsible for the overall control of the trial within the framework of the protocol; for recruitment of new centres; for organizing yearly meetings; etc.
   - secretary to be decided.

2. Ethical Committee

   - members: to be decided
   - meets when necessary for ethical reasons or for application of stopping guidelines
   - reviews all data and decides on stopping the trial.

3. Advisers

   - members: to be decided: scientists who advise on scientific matters of the trial

4. Monitoring Committee

   - members: to be decided (proposal: C.J. Bulpitt, A. Fletcher, J. Staessen, L. Thijs)
   - elects its chairman
   - meets at monthly intervals
   - reviews all data and reports to Ethical and Steering Committees

5. Drug Committee

- members: a representative of the Ethical Committee and Monitoring Committee and representatives of the pharmaceutical companies which provide the drugs: e.g. Dr. Ziegler, Dr. Verhaest.
- responsible for drug conditioning and dispatching

6. EEC-Project Management Group

- following members already accepted to serve in this committee: A. Amery, W. Birkenhäger, F. Bühler, F. de Padua, C. Dollery, G. Fodor, F. Forette, D. Ganten, L. Hanssen, K. O'Malley, J. Rodicio, T. Strasser, A. Tourkantonis, J. Tuomilehto, Ch. van Ypersele de Strihou, A. Zanchetti
- task: contact with the EEC

7. National Committees

- members: all collaborators of each country

8. Coordinating Office

- will be responsible for the day-to-day conduct of the study; stratification and randomization of patients; data processing
- reports to the Monitoring Committee and/or to the Steering Committee, depending on the subject

9. Regional Drug Dispatching Centres

- centres in each country or groups of countries
- these centres are responsible for the drug supply to the individual centres in their area. In addition, the responsible person in the Regional Drug Dispatching centre writes the patient number on the bottles with study medication.

10. Committees for Side- Projects

For each side-project a committee will be nominated.

APPENDIX XIII

LIST OF ADDRESSES

* Prof. Dr. A. AMERY
Internal Medicine-Cardiology
University Hospital Gasthuisberg
Herestraat 49
B-3000 LEUVEN
Belgium

* Prof. Dr. W. BIRKENHÄGER
Zuiderziekenhuis
Inwendige Geneeskunde
Groene Hilledijk 135
N-3075 EA ROTTERDAM
The Netherlands

* Prof. Dr. F. BÜHLER
Department of Research
University Hospital
CH-4031 BASEL
Switzerland

* Prof. Dr. C.J. BULPITT
Geriatric Unit
Hammersmith Hospital
Du Cane Raod
LONDON W12 0HS
U.K.

* Dr. M.O. CARRAGETA
Hospital de Santa Maria
Nucleo de Cardiologia Preventiva
Terapeutica Medica - Piso 4
Avenida Egaz Moniz
LISBOA 4
Portugal

* Dr. A. CHOCKALINGHAM
Assistant Professor
Faculty of Medicine
Memorial University St. John's
NEWFOUNDLAND A1B 3V6
Canada

* Prof. Dr. D. CLEMENT
Universitair Ziekenhuis
Afdeling Cardiologie
De Pintelaan 185
B-9000 GENT
Belgium

* Dr. P. CONNE
Médecin-Chef de l'Hôpital de Loëx
Institutions Universitaires de
Gériatrie-Genève
Route de Mon-Idée
CH-1226 GENEVE-THONEX
Switzerland

* Dr. J. COX
Beaumont Hospital
Royal College of Surgeons
St. Stephens' Green
DUBLIN 2
Ireland

* Dr. P. de LEEUW
Zuiderziekenhuis
Inwendige Geneeskunde
Groene Hilledijk 315
N-3075 EA ROTTERDAM
The Netherlands

* Dr. F. de PADUA
Hospital de Santa Maria
Nucleo de Cardiologia Preventiva
Terapeutica Medica - Piso 4
Avenida Egaz Moniz
LISBOA 4
Portugal

* Dr. J.F. DE PLAEN
Chef de Clinique
Cliniques Universitaires Saint-Luc
Service de Néphrologie
10, Avenue Hippocrate
B-1200 BRUXELLES
Belgium

* Prof. Dr. A. DE SCHAEPDRYVER
Heymans Instituut
De Pintelaan 185
B-9000 GENT
Belgium

* Prof. Dr. C.T. DOLLERY
Dept. of Clinical Pharmacology
Royal Postgraduate Medical School
Hammersmith Hospital
Ducane Road
LONDON W12 OHS
England

* Prof. Dr. P. DUPONT
C.H. de Tivoli
Dép. de Néphrologie et d'Hypertension
34, Avenue Max Buzet
B-7100 LA LOUVIERE
Belgium

* Dr. D. DUPREZ
Universitair Ziekenhuis
Afdeling Cardiologie
De Pintelaan 185
B-9000 GENT
Belgium

* Prof. Dr. R. FAGARD
U.Z. Gasthuisberg
Inwendige Geneeskunde
Herestraat 49
B-3000 LEUVEN
Belgium

* Dr. A. FLETCHER
Geriatric Unit
Hammersmith Hospital
Du Cane Road
LONDON W12 OHS
England

* Dr. J.G. FODOR
Prof. of Clinical Epidemiology
Faculty of Medicine
Memorial University St. John's
NEWFOUNDLAND A1B 3V6
Canada

* Dr. F. FORETTE
Chef de Service
Hôpital Broca
54/56 rue Pascal
75013 PARIS
France

* Dr. J. FORTE
Hospital de Santa Maria
Cardiologia Preventiva - Piso 4
Avenida Egaz Moniz
LISBOA 4
Portugal

* Dr. D. GANTEN
German Institute for High Blood
Pressure Research
Im Neuenheimer Feld 366
6900 HEIDELBERG
Federal Republic of Germany

* Dr. L. GAUTHEY
1er Chef de Clinique
Hôpital de Gériatrie
Institutions Universitaires
de Gériatrie-Genève
Route de Mon-Idée
CH-1226 GENEVE-THONEX
Switzerland

* Dr. J.F. HENRY
Hôpital Broca
54/56 rue Pascal
75013 PARIS
France

* Prof. Dr. G. HITZENBERGER
I. Medizinische Universitätsklinik
Abteilung für Klinische Pharmakologie
Lazarettgasse 14
A-1090 WIEN
Austria

* Dr. M.J. JAMIESON
Clinical Pharmacology Unit
Dept. Medicine/Therapeutics
University of Aberdeen
Polwarth Building
Foresterhill
ABERDEEN AB9 2ZB
U.K.

* Dr. T.A. JEFFERS
Clinical Pharmacology Unit
Dept. Medicine/Therapeutics
University of Aberdeen
Polwarth Building
Foresterhill
ABERDEEN AB9 2ZB
U.K.

* Dr.W. KIOWSKI
Dept. of Cardiology
University Hospital
CH-4031 BASEL
Switzerland

* Prof. Dr. S.K. KIVELA
Dept. Public Health
University of Oulu
Aapistie 3
90220 OULU
Finland

* Dr. A. KOISTINEN
Joensuu Health Centre
Noljakantie 17
80130 JOENSU
Finland

* Dr. J.M. KRZESINSKI
C.H.U. Sart Timan
Service de Néphrologie
B-4000 LIEGE 1
Belgium

* Mr M. LAERMANS
Hypertension Unit
Research Building
Campus Gasthuisberg
Herestraat 49
B-3000 LEUVEN
Belgium

* Dr. J. LAVAIN
Beaumont Hospital
Beaumont
DUBLIN 9
Ireland

* Dr. M. LASZL
Center for Prevention of
Cardiovascular Diseases
Barzilai Medical Center
6 Alizar Tager Street
AFRIDAR ASHKELON 78416
Israël

* Dr. A. LAZARIDIS
Assistant Professor
First Dept. Internal Med.
University Hospital AHEPA
54006 THESSALONIKI
Greece

* Dr. G. LEONETTI
Centro di Fisiologia Clinica e
Ipertensione
Ospedale Maggiore
Univ. di Milano
Via F. Sforza 35
20133 MILAN
Italy

* Dr. T.F. LÜSHER
Department of Research
University Hospital
CH-4031 BASEL
Switzerland

* Prof. Dr. G. MANCIA
Centro di Fisiologia Clinica e
Ipertensione
Ospedale Maggiore
Università di Milano
Via F. Sforza 35
20122 MILAN
Italy

* Dr. MANN
Medizinische Universitätsklinik
Sektion Néphrologie
Bergheimer Strasse 56a
D-6900 HEIDELBERG
Federal Republic of Germany

* Dr. T. McDONALD
Clinical Pharmacology Unit
Dept. Medicine/Therapeutics
University of Aberdeen
Polwarth Building
Foresterhill
ABERDEEN AB9 2ZB
U.K.

* Prof. Dr. J.P. MICHEL
Médecin-Chef
Institutions Universitaires
de Gériatrie-Genève
Route de Mon-Idée
CH-1226 GENEVE-THONEX
Switzerland

* Prof. A. NISSINEN
University of Kuopio
Dept. Community Health
P.O. Box 670211
KUOPIO
Finland

* Dr. J. NOEL
Beaumont Hospital
Beaumont
DUBLIN 9
Ireland

* Dr. E. O'BRIEN
Beaumont Hospital
Beaumont
DUBLIN 9
Ireland

* Prof. Dr. K. O'MALLEY
Royal College of Surgeons
St. Stephens' Green
DUBLIN 2
Ireland

* Dr. G. PARATI
Centro di Fisiologia Clinica e
Ipertensione
Università di Milano
Ospedale Maggiore
Via F. Sforza 35
20122 MILAN
Italy

* Prof. Dr. J.C. PETRIE
Clinical Pharmacology Unit
Dept. Medicine/Therapeutics
University of Aberdeen
Polwarth Building
Foresterhill
ABERDEEN AB9 2ZB
U.K.

* Dr. E. RITZ
Medizinische Universitätsklinik
Sektion Nephrologie
Bergheimer Strasse 56a
D-6900 HEIDELBERG
Federal Republic of Germany

* Prof. Dr. J.L. RODICIO
Hospital 1° Octubre
Carretara Andalucia
km. 5.400
28041 MADRID
Spain

* Dr. G. RORIVE
C.H.U. Sart Timan
Service de Néphrologie
B-4000 LIEGE 1
Belgium

* Prof. Dr. J.B. ROSENFELD
Beilinson Medical Center
PETAH TIKVA 49100
Israël

* Dr. J. ROSENTHAL
Ulm University Medical Center
Steinhoevelstrasse
D-7900 ULM (Donau)
Germany

* Dr. M. SAFAR
Centre de Diagnostic
Hôpital Broussais
96, rue Didot
75674 PARIS
France

* Dr. V. SASDOVITCH
Hôpital Broca
54/56 rue Pascal
75013 PARIS
France

* Dr. D. SLOVIC
Geriatric Unit
Hammersmith Hospital
Du Cane Road
LONDON W12 0HS
U.K.

* Dr. J. STAESSEN
Internal Medicine-Cardiol.
University Hospital
Gasthuisberg
Herestraat 49
B-3000 LEUVEN
Belgium

* Dr. T. STRASSER
World Hypertension League
20, Avenue du Bouchet
1209 GENEVA
Switzerland

* Drs. L. THIJS
Hypertension Unit
Research Building
Campus Gasthuisberg
Herestraat 49
B-3000 LEUVEN
Belgium

* Prof. A. TOURKANTONIS
Director and Chairman
First Dept. Internal Med.
University Hospital AHEPA
54006 THESSALONIKI
Greece

* Prof. Dr. TUOMILEHTO
National Public Health Institute
Department of Epidemiology
Hämeentie 155
00560 HELSINKI
Finland

* Prof. Dr. Ch. van Ypersele
Chef de Service
Cliniques Universitaires Saint-Luc
Service de Néphrologie
10, Avenue Hippocrate
B-1200 BRUXELLES
Belgium

* Dr. L. VERHAEST
M.S.D.
Steenweg op Waterloo, 1135
B-1180 BRUSSEL
België

* Dr. R.J. VISKOPER
Director
Center for Prevention of
Cardiovascular Diseases
Barzilai Medical Center
6 Alizar Tager Street
AFRIDAR ASHKELON 78416
Israël

* Dr. J. WEBSTER
Clinical Pharmacology Unit
Dept. Medicine/Therapeutics
University of Aberdeen
Polwarth Building
Foresterhill
ABERDEEN AB9 2ZB
U.K.

* Dr. J. ZABLUDOWSKI
Beilinson Medical Center
PETAH TIKVA 49100
Israël

* Prof. Dr. A. ZANCHETTI
Centro di Fisiologia Clinica e
Ipertensione
Ospedale Maggiore
Università di Milano
Via F. Sforza 35
20122 MILAN
Italy

* Dr. med. R. ZIEGLER
Bayer AG
Geschäftsbereich Pharma EP
Klifo 1-Hochdruck
Pharma-Forschungszentrum
Aprather Weg - Postfach 10 17 09
D-5600 WUPPERTAL 1
Germany

SYST EUR

SHORT REPORT DURING PLACEBO RUN-IN PERIOD

1/2

IDENTIFICATION & REGISTRATION (1) [redacted]

Visit : First or third visit (3)

Date of visit : ☐☐ ☐☐ ☐☐
day month year

Patient

Name or initials : (2)

....

Date of birth :

☐☐ ☐☐ ☐☐
day month year

Centre

Centre name :

....

Name of doctor : (2)

....

INFORMATION ON TREATMENT

Study medication at moment of visit

Hypotensor A

(1111)

Dose ? (tablets/day) ☐.☐
- 1/2 tablet per day = 0.5
- 1 tablet per day = 1.0
- 1 1/2 tablets per day = 1.5
- 2 tablets per day = 2.0

Dose reduction or interruption since last visit ? ☐
- 2 = no,
- 3 = yes, dose reduction
- 4 = yes, interruption,
- 8 = not applicable, 9 = unknown

If yes, reason(s) for dose reduction or interruption
1. .... ☐ ☐☐☐.☐
2. .... ☐ ☐☐☐.☐
3. .... ☐ ☐☐☐.☐

Any other drugs (e.g. aspirin, laxatives)

Drug 1

Name : .... ☐☐☐ / ☐☐☐☐☐

Drug 2

Name : .... ☐☐☐ / ☐☐☐☐☐

Drug 3

Name : .... ☐☐☐ / ☐☐☐☐☐

Drug 4

Name : .... ☐☐☐ / ☐☐☐☐☐

Drug 5

Name : .... ☐☐☐ / ☐☐☐☐☐

Drug 6

Name : .... ☐☐☐ / ☐☐☐☐☐

acco D1900A—1989

(1) To be completed at the first and third out-patient visit during the placebo run-in period.
(2) Please print.
(3) Circle correct answer.

☐ for C.O.

ORIGINAL

PHYSICAL EXAMINATION

Blood pressure (mmHg)		supine	sitting(5min)	standing(2min)
1	SBP			
	DBP (IV)*			
	DBP (V)			
2	SBP			
	DBP (IV)*			
	DBP (V)			

Sitting pulse rate (bpm)

DECISIONS & COMMENTS

Does the patient continue placebo run-in period ?(1)

[illegible] yes 2 = no 9 = unknown

If no, specify reason :

Comments :

(1) Please reply yes (code = 1) when after third run-in visit the patient is continued on placebo tablets before being randomized.

* Optional

for C.O.

ORIGINAL

SYST-EUR

SHORT REPORT DURING DOUBLE-BLIND PERIOD

IDENTIFICATION & REGISTRATION (1)

Syst-Eur patient number : ☐☐☐ ☐ ☐☐☐ ☐☐

Date of visit : ☐☐ day ☐☐ month ☐☐ year

Patient

Name or initials : (2)

....

Date of birth :

☐☐ day ☐☐ month ☐☐ year

Centre

Centre name :

....

Name of doctor : (2)

....

INFORMATION ON TREATMENT

Study medication at moment of visit

	Hypotensor A	Hypotensor B	Hypotensor C
Dose ? (tablets/day)	☐.☐	☐.☐	☐.☐
Dose reduction or interruption since last visit ?<br>2 = no, 3 = yes, dose reduction<br>4 = yes, interruption,<br>8 = not applicable, 9 = unknown	☐	☐	☐
If yes, reason for dose reduction or interruption	..........<br>..........<br>..........	..........<br>..........<br>..........	..........<br>..........<br>..........

Any other drugs (e.g. aspirin, laxatives)

Drug 1

Name : ....

☐☐☐ / ☐☐☐☐☐

Drug 2

Name : ....

☐☐☐ / ☐☐☐☐☐

Drug 3

Name : ....

☐☐☐ / ☐☐☐☐☐

Drug 4

Name : ....

☐☐☐ / ☐☐☐☐☐

Drug 5

Name : ....

☐☐☐ / ☐☐☐☐☐

Drug 6

Name : ....

☐☐☐ / ☐☐☐☐☐

acco D781A—1988

1. **To be completed at quarterly intervals during double-blind treatment.**
   If a patient leaves the double-blind part of the study, a special form "Report at the End of the Double-Blind Period" should be completed.
2. **Please print.**

☐ for C.O.

ORIGINAL

[redacted]

2 ....

3 ....

4 ....

5 ....

PHYSICAL EXAMINATION

Blood pressure (mmHg)

		supine	sitting(5min)	standing(2min)
1	SBP			
	DBP (IV)*			
	DBP (V)			
2	SBP			
	DBP (IV)*			
	DBP (V)			

Sitting pulse rate (bpm)

Other physical signs ? 1 = yes, 2 = no

If yes, specify :

1 ....

2 ....

3 ....

4 ....

5 ....

DISEASES & FINDINGS

New diseases & findings since last visit ?
(including dementia)

1 ....

2 ....

3 ....

4 ....

5 ....

Previous diseases & findings still active ?
(including dementia)

1 ....

2 ....

3 ....

4 ....

5 ....

DECISIONS & COMMENTS

Does the patient continue double-blind study ?(1)

1 = yes, 2 = no, 9 = unknown

If no, specify reason :

....

....

....

Comments :

....

....

....

* **Optional**

** **Only compulsory during first year of ACE inhibition (otherwise optional)**

(1) If a patient leaves the double-blind part of the study, a special form "Report at the End of the Double-Blind Period" should be completed.

for C.O. **ORIGINAL**

SYST-EUR

EXTENSIVE REPORT DURING PLACEBO RUN-IN PERIOD

IDENTIFICATION & REGISTRATION (1)

Visit : Second visit

Date of visit : ☐☐ day ☐☐ month ☐☐ year

Patient

Name or initials : (2)

....

Gender : ☐ M = male, F = female

Date of birth :

☐☐ day ☐☐ month ☐☐ year

Centre

Centre name :

....

Name of doctor : (2)

....

LOGBOOK

Please indicate where the patient was entered in your logbook :

Logbook number : ☐☐ **Row number :** ☐☐☐

HISTORY

Personal history

Present diseases

1 ....

2 ....

3 ....

4 ....

5 ....

**Past diseases and operations**	**year of last occurrence**
1	19 ☐☐
2	19 ☐☐
3	19 ☐☐
4	19 ☐☐
5	19 ☐☐

Family history

Is father deceased ? ☐ 1 = yes, 2 = no, 9 = unknown

If yes : age of death ☐☐☐ years

cause of death ....

....

Is mother deceased ? ☐ 1 = yes, 2 = no, 9 = unknown

If yes : age of death ☐☐☐ years

cause of death ....

....

(1) To be completed at the 2nd visit during the placebo run-in period.

(2) Please print.

☐ for C.O.

ORIGINAL

DIAGNOSIS OF HYPERTENSION (HT)

Stage of HT : ☐

1 = HT without organ involvement
2 = HT with LVH only.
3 = HT with MI or angina only.
4 = malignant HT
5 = HT with CNS involvement only.
6 = HT with renal involvement only.
7 = HT with eye fundus grade III only.
8 = HT with multiple organ involvement.
9 = unknown.

Aetiological diagnosis of HT :

Essential HT : ☐ 1 = yes (certain), 2 = no, 3 = possible, 4 = unlikely, 9 = unknown

If secondary HT, specify cause :

....

SMOKING & DRINKING HABITS

Is patient ex-smoker ? ☐

Is patient smoking now ? ☐

1 = yes,
2 = no,
9 = unknown

If yes, give consumption per **day** of :

cigarettes	cigars	pipes
☐☐	☐☐	☐☐

Does the patient consume alcohol now ? ☐

1 = yes,
2 = no,
9 = unknown

If yes, how many glasses per **week** of :

beer	wine	spirits	apéritif or fortified wine
☐☐☐	☐☐☐	☐☐☐	☐☐☐

ACTIVITIES OF DAILY LIVING (ADL)

if "yes" score = 1 if "no" score = 0

Can the patient bath alone ? ☐

Can the patient dress alone ? ☐

Can the patient go to the toilet alone ? ☐

Can the patient move in and out bed and chair alone ? ☐

Is the patient entirely self-controlled for urination and defaecation ? ☐

Can the patient eat alone ? ☐

Total ADL score (sum of 6 scores) : ☐

EDUCATION

Patient followed full-time school until age : ☐☐

INFORMATION ON TREATMENT

Previous antihypertensive treatment

Was the patient on treatment with antihypertensive drugs during **the last 6 months** ?

☐ 1 = yes, 2 = no, 9 = unknown

If yes, specify if the patient was on one of the following antihypertensive drugs :

1 = yes,
2 = no,
3 = unknown

— Centrally acting drugs ? ☐

— Diuretics ? ☐

— Beta-blockers ? ☐

— Calcium entry blockers ? ☐

— ACE-inhibitors ? ☐

— Other antihypertensive drugs ? ☐

Study medication at moment of visit

Hypotensor A
(1111)

Dose ? (tablets/day) ☐.☐

1/2 tablet per day = 0.5
1 tablet per day = 1.0
1 1/2 tablets per day = 1.5
2 tablets per day = 2.0

Dose reduction or interruption since last visit ? ☐

2 = no,
3 = yes, dose reduction
4 = yes, interruption,
8 = not applicable,
9 = unknown

If yes, reason(s) for dose reduction or interruption :

1 ....

2 ....

3 ....

☐ for C.O.

ORIGINAL

Any other drugs (e.g. aspirin, laxatives)

Drug 1

Name : .... /

Drug 2

Name : .... /

Drug 3

Name : .... /

Drug 4

Name : .... /

Drug 5

Name : .... /

Drug 6

Name : .... /

PHYSICAL EXAMINATION

Blood pressure (mmHg)		supine	sitting(5min)	standing(2min)
1	SBP			
	DBP (IV)*			
	DBP (V)			
2	SBP			
	DBP (IV)*			
	DBP (V)			

Sitting pulse rate (bpm)

Weight (kg) .

Height (cm) : .

Fundoscopy — **Right eye** — **Left eye**

(stage Keith-Wagener)

1 = grade I, 2 = grade II, 3 = grade III, 4 = papilloedema,
5 = lens opacity, 8 = normal, 9 = unknown

Other physical signs ? 1 = yes, 2 = no

If yes, specify :

1 ....

2 ....

3 ....

4 ....

5 ....

URINE TESTS

Proteinuria

Glucosuria

1 = positive,
2 = traces,
3 = negative,
9 = unknown

White blood cells

Red blood cells

Granular casts

1 = many (≧ 10 per field),
2 = rare,
3 = none,
9 = unknown

BLOOD TESTS

Was patient fasting ? 1 = yes, 2 = no, 9 = unknown

Haematocrit (%)

Haemoglobin (mmol/L)

WBC (10^9 cells/L)

RBC (10^{12}cells/L)

Creatinine (μmol/L)

Uric acid (mmol/L)

Na (mmol/L)

K (mmol/L)

Glucose (mmol/L)

Cholesterol :
— total (mmol/L)
— HDL (mmol/L)

Triglycerides (g/L)

Alk. phosph. (U/L)

SGOT (U/L)

SGPT (U/L)

gGT (U/L)

Insulin (μU/ml)*

* Optional

for C.O.

ORIGINAL

ELECTROCARDIOGRAM

ECG normal ? ☐ 1 = yes, 2 = no, 8 = not performed, 9 = unknown

If abnormal, specify : ....

....

....

Original sent to C.O. on ☐☐ day ☐☐ month ☐☐ year

If not sent, indicate reason : ....

....

....

Height of : calibration signal (1 mV) ☐☐ (mm)

RaVL ☐☐ (mm)

SV_1 ☐☐ (mm)

RV_5 ☐☐ (mm)

Atrial fibrillation ☐ 1 = yes, 2 = no, 9 = unknown

OPTIONAL EXAMINATIONS

Will the patient participate in the following side-projects ?

1. Quality of live assessment* ☐
2. 24-h BP recordings* ☐
3. Storage of serum* ☐
4. Multiple infarction dementia* ☐

1 = yes, 2 = no, 9 = unknown

Were the following examinations performed ?

1. Chest X-ray* ☐ 1 = yes, 2 = no, 9 = unknown

cardiac diameter ☐☐☐ (mm)

thoracic diameter ☐☐☐ (mm)

2. Intravenous pyelography* ☐ 1 = normal, 2 = abnormal, 8 = not performed, 9 = unknown

If abnormal, specify : ....

....

....

3. Renal angiography* ☐ 1 = normal, 2 = abnormal, 8 = not performed, 9 = unknown

If abnormal, specify : ....

....

....

DECISIONS & COMMENTS

Does the patient continue placebo run-in period ?

☐ 1 = yes, 2 = no, 9 = unknown

If no, specify reason :

....

....

....

Comments :

....

....

....

*Optional ▒ for C.O.

ORIGINAL

SYST-EUR

CHECKLIST

IDENTIFICATION & REGISTRATION (1)

Date of visit : ☐☐ ☐☐ ☐☐
day month year

PATIENT	CENTRE
Name of initials : (2)	Centre name : (2) ....
Date of birth : ☐☐ ☐☐ ☐☐ (day, month, year)	Name of doctor : (2) ....

A. INCLUSION CRITERIA (3)

	Yes	No
1. 60 years or older	☐	☐
2. BP during run-in period at 3 visits combined (= average of 3 x 2 readings)		
— SBP_{sit} : 160-219 mmHg	☐	☐
— DBP_{sit} : ≦ 94 mmHg	☐	☐
— SBP_{stand} : ≧ 140 mmHg	☐	☐
3. Patient willing to cooperate and regular follow-up is possible	☐	☐

for C.O. only

Gender ☐
Complications ☐
Stratum ☐
SBP_{sit} ☐☐☐
DBP_{sit} ☐☐☐
SBP_{stand} ☐☐☐
Supervisor ☐☐☐☐
☐☐☐ ☐ ☐☐☐ ☐☐

B. EXCLUSION CRITERIA (3)

	Yes	No
**1. Certain causes of BP elevation**		
a. Specific cause of SBP elevation, such as hyperthyroidism	☐	☐
b. Conditions which will be corrected by surgery	☐	☐
c. Severe aortic insufficiency, A-V fistula	☐	☐
**2. Certain complications of hypertension**		
a. Presence at recruitment of		
— vascular retinopathy grade III or IV	☐	☐
— overt congestive heart failure	☐	☐
— dissecting aneurysm	☐	☐
— severe renal failure (serum creatinine 180 µmol/L or more)	☐	☐
b. History of		
— repeated severe nose bleeding due to hypertension	☐	☐
— cerebral or subarachnoid haemorrhage	☐	☐
— myocardial infarction < 1 year before randomization	☐	☐
**3. Other diseases**		
— dementia	☐	☐
— hepatic dysfunction	☐	☐
— malignant neoplasm	☐	☐
— diabetes mellitus with frequent insulin adjustments	☐	☐
— patient permanently bed-ridden	☐	☐
— need for drugs with BP lowering activity	☐	☐
**4. Poor collaboration**	☐	☐

acco D1900C—1989

(1) Please complete the checklist together with the report of the third visit during the placebo run-in period and send both together to the Coordinating Office.
(2) Please print.
(3) Tick correct answer.

ORIGINAL

SYST-EUR
EXTENSIVE REPORT DURING DOUBLE-BLIND PERIOD

IDENTIFICATION & REGISTRATION (1)

Syst-Eur patient number : ☐☐☐ ☐ ☐☐☐ ☐☐

Date of visit : ☐☐ day ☐☐ month ☐☐ year

Patient	Centre
**Name or initials : (2)**	**Centre name :** ....
**Date of birth :** ☐☐ day ☐☐ month ☐☐ year	**Name of doctor : (2)** ....

INFORMATION ON TREATMENT

Study medication at moment of visit

	Hypotensor A	Hypotensor B	Hypotensor C
Dose ? (tablets/day)	☐.☐	☐.☐	☐.☐
Dose reduction or interruption since last visit ?<br>2 = no, 3 = yes, dose reduction<br>4 = yes, interruption,<br>8 = not applicable, 9 = unknown	☐	☐	☐
If yes, reason for dose reduction or interruption	....................<br>....................<br>....................	....................<br>....................<br>....................	....................<br>....................<br>....................
	☐ ☐☐☐.☐<br>☐ ☐☐☐.☐<br>☐ ☐☐☐.☐	☐ ☐☐☐.☐<br>☐ ☐☐☐.☐<br>☐ ☐☐☐.☐	☐ ☐☐☐.☐<br>☐ ☐☐☐.☐<br>☐ ☐☐☐.☐

Any other drugs (e.g. aspirin, laxatives)

Drug 1

Name : ....

☐☐☐ / ☐☐☐☐☐

Drug 2

Name : ....

☐☐☐ / ☐☐☐☐☐

Drug 3

Name : ....

☐☐☐ / ☐☐☐☐☐

Drug 4

Name : ....

☐☐☐ / ☐☐☐☐☐

Drug 5

Name : ....

☐☐☐ / ☐☐☐☐☐

Drug 6

Name : ....

☐☐☐ / ☐☐☐☐☐

acco D781B—1988

1. **To be completed at yearly intervals during double-blind treatment.**
   If a patient leaves the double-blind part of the study, a special form "Report at the End of the Double-Blind Period" should be completed.
2. **Please print.**

☐ for C.O

ORIGINAL

SMOKING & DRINKING HABITS

Is patient smoking now ? ☐ 1 = yes, 2 = no, 9 = unknown

If yes, give consumption per <u>day</u> of :

☐☐ cigarettes ☐☐ cigars ☐☐ pipes

Does the patient consume alcohol now ? ☐ 1 = yes, 2 = no, 9 = unknown

If yes, how many glasses per **week** of :

☐☐☐ beer ☐☐☐ wine

☐☐☐ spirits ☐☐☐ apéritif or fortified wine

ACTIVITIES OF DAILY LIVING (ADL)

if "yes" score = 1 if "no" score = 0

Can the patient bath alone ? ☐

Can the patient dress alone ? ☐

Can the patient go to the toilet alone ? ☐

Can the patient move in and out bed and chair alone ? ☐

Is the patient entirely self-controlled for urination and defaecation ? ☐

Can the patient eat alone ? ☐

Total ADL score (sum of 6 scores) : ☐

HISTORY

Did patient have any complaints since last visit ?

☐ 1 = yes, 2 = no, 9 = unknown

If yes, specify :

1 .... ☐ ☐☐☐.☐

2 .... ☐ ☐☐☐.☐

3 .... ☐ ☐☐☐.☐

4 .... ☐ ☐☐☐.☐

5 .... ☐ ☐☐☐.☐

PHYSICAL EXAMINATION

**Blood pressure (mmHg)**		**supine**	**sitting(5min)**	**standing(2min)**
1	**SBP**	☐☐☐	☐☐☐	☐☐☐
	**DBP (IV)***	☐☐☐	☐☐☐	☐☐☐
	**DBP (V)**	☐☐☐	☐☐☐	☐☐☐
2	**SBP**	☐☐☐	☐☐☐	☐☐☐
	**DBP (IV)***	☐☐☐	☐☐☐	☐☐☐
	**DBP (V)**	☐☐☐	☐☐☐	☐☐☐

Sitting pulse rate (bpm) ☐☐☐

Weight (kg) ☐☐☐.☐

Fundoscopy **Right eye** ☐ **Left eye** ☐

(stage Keith-Wagener)

1 = grade I, 2 = grade II, 3 = grade III, 4 = papilloedema, 5 = lens opacity, 8 = normal, 9 = unknown

Other physical signs ? ☐ 1 = yes, 2 = no

If yes, specify :

1 .... ☐ ☐☐☐.☐

2 .... ☐ ☐☐☐.☐

3 .... ☐ ☐☐☐.☐

4 .... ☐ ☐☐☐.☐

5 .... ☐ ☐☐☐.☐

URINE TESTS

Proteinuria ☐

Glucosuria ☐

1 = positive, 2 = traces, 3 = negative, 9 = unknown

White blood cells ☐

Red blood cells ☐

Granular casts ☐

1 = many (≧ 10 field), 2 = rare, 3 = none, 9 = unknown

Was urine sample sent for evaluation of compliance ?

— If yes, specify date : ☐☐ day ☐☐ month ☐☐ year

— If not, indicate reason, why not : ....

....

☐☐

* Optional ☐ for C.O. ORIGINAL

BLOOD TESTS

Was patient fasting ? ☐ 1 = yes, 2 = no, 9 = unknown

Haematocrit (%) ☐☐.☐

Haemoglobin (mmol/L) ☐☐.☐

WBC (10^9 cells/L) ☐☐.☐

RBC (10^{12}cells/L) ☐.☐☐

Creatinine (μmol/L) ☐☐☐.☐

Uric acid (mmol/L) ☐.☐☐

Na (mmol/L) ☐☐☐.☐

K (mmol/L) ☐.☐☐

Glucose (mmol/L) ☐☐.☐

Cholesterol :
— total (mmol/L) ☐☐.☐
— HDL (mmol/L) ☐☐.☐

Triglycerides (g/L) ☐☐.☐

Alk. phosph. (U/L) ☐☐☐

SGOT (U/L) ☐☐☐

SGPT (U/L) ☐☐☐

gGT (U/L) ☐☐☐

Insulin (U/ml)* ☐☐☐

ELECTROCARDIOGRAM

ECG normal ? ☐ 1 = yes, 2 = no, 8 = not performed, 9 = unknown

If abnormal, specify : ....

....

....

☐☐☐☐☐ ☐☐☐☐☐ ☐☐☐☐☐

Original sent to C.O. on ☐☐ day ☐☐ month ☐☐ year

If not sent, indicate reason : ....

....

.... ☐☐

Height of : calibration signal (1 mV) ☐☐ (mm)

RaVL ☐☐ (mm)

SV_1 ☐☐ (mm)

RV_5 ☐☐ (mm)

Atrial fibrillation ☐ 1 = yes, 2 = no, 9 = unknown

DISEASES & FINDINGS

New diseases & findings since last visit ?
(including dementia)

1 .... ☐ ☐☐☐.☐

2 .... ☐ ☐☐☐.☐

3 .... ☐ ☐☐☐.☐

4 .... ☐ ☐☐☐.☐

5 .... ☐ ☐☐☐.☐

Previous diseases & findings still active ?
(including dementia)

1 .... ☐ ☐☐☐.☐

2 .... ☐ ☐☐☐.☐

3 .... ☐ ☐☐☐.☐

4 .... ☐ ☐☐☐.☐

5 .... ☐ ☐☐☐.☐

OPTIONAL EXAMINATIONS

Did the patient participate in one of the following side-projects ?

1. Quality of live assessment* ☐
2. 24-h BP recordings* ☐
3. Storage of serum* ☐
4. Multiple infarction dementia* ☐

1 = yes,
2 = no,
9 = unknown

DECISIONS & COMMENTS

Does the patient continue double-blind study ?(1) ☐ 1 = yes, 2 = no, 9 = unknown

If no, specify reason :

....

....

....

☐ ☐☐☐.☐/☐ ☐☐☐.☐/☐ ☐☐☐.☐

Comments :

....

....

....

☐ ☐☐☐.☐/☐ ☐☐☐.☐/☐ ☐☐☐.☐

(1) If a patient leaves the double-blind part of the study, a special form "report at the End of the Double-Blind Period" should be completed.

*Optional ☐ for C.O. ORIGINAL

SYST[redacted]
[redacted]RT AT THE [redacted] OF THE [redacted]-BLIND [redacted]

IDENTIFICATION & REGISTRATION (1)

Syst-Eur patient number : ☐☐☐ ☐ ☐☐☐ ☐☐

Date of report : ☐☐ (day) ☐☐ (month) ☐☐ (year)

Patient

Name or initials : (2)

....

Date of birth :

☐☐ (day) ☐☐ (month) ☐☐ (year)

Centre

Centre name :

....

Name of doctor : (2)

....

DATE

Specify date of major fatal or non-fatal event, date of patient withdrawal, date on which patient defected from study or date of missed appointment :

☐☐ (day) ☐☐ (month) ☐☐ (year)

REASON

Specify reason why double-blind treatment was stopped:

Death ☐ 1 = yes, 2 = no

If yes, cause of death :

Immediate cause : ....

....

Underlying cause(s) :

1. ....

2. ....

Contributory cause(s) :

1. ....

2. ....

If yes, was autopsy performed, ☐
and
was protocol sent to C.O. ? ☐

1 = yes,
2 = no,
9 = unknown

Non-fatal events (1 = yes, 2 = no)

— **Cerebrovascular accident (excluding TIA)**

> cerebral haemorrhage ☐

> subarachnoid haemorrhage ☐

> thrombosis ☐

> embolism ☐

— **Retinal lesions**

> exudates or haemorrhage ☐

> papilloedema ☐

— **Myocardial infarction** ☐

— **Congestive heart failure,** requiring diuretics or vasodilators or any antihypertensive drug ☐

— **Dissecting aneurysm** ☐

— **Increase in serum creatinine** by 100 % or to 360 μmol/L (2mg/dl) at 2 consecutive visits ☐

Withdrawal

— **Systolic or diastolic blood pressure too high** ☐

— **Prescription of antihypertensive drugs (≧ 3 months)** ☐

If yes, specify why :

1. ....

2. ....

— **Code broken** ☐

If yes, specify why :

1. ....

2. ....

---

(1) To be completed at end of double-blind period.
(2) Please print.

☐ for C.O.

[redacted] [redacted] [redacted] [redacted]

1 = yes
2 = no

— **Interfering disease(s) or adverse drug effect(s)** ☐

If yes, specify :

1. ....

2. ....

— **Patient followed for 7 years on double-blind medication** ☐

— **End of whole trial** ☐

Defector

Patient refuses : ☐

— **to attend clinic**

If yes, specify why :

1. ....

2. ....

1 = yes
2 = no

— **to take any double-blind medication** ☐

If yes, specify why :

1. ....

2. ....

Other reason(s) ☐

If yes, specify :

1. ....

2. ....

FOLLOW-UP

Has the patient been examined at clinic ?

☐ 1 = yes, 2 = no, 9 = unknown

If yes, please complete the following sections on pages 2 through 5.

INFORMATION ON TREATMENT

Study medication at moment of visit

	Hypotensor A	Hypotensor B	Hypotensor C
Dose ? (tablets/day)	☐.☐	☐.☐	☐.☐
Dose reduction or interruption since last visit ?	☐	☐	☐
If yes, reason for dose reduction or interruption	....	....	....

2 = no, 3 = yes, dose reduction
4 = yes, interruption,
8 = not applicable, 9 = unknown

☐ for C.O.

ORIGINAL

[redacted] [redacted]ugs (e.g. asp[redacted] [redacted]

Drug 1

Name : .... /

Drug 2

Name : .... /

Drug 3

Name : .... /

Drug 4

Name : .... /

Drug 5

Name : .... /

Drug 6

Name : .... /

HISTORY

Did patient have any complaints since last visit ?

☐ 1 = yes, 2 = no, 9 = unknown

If yes, specify :

1 ....

2 ....

3 ....

4 ....

5 ....

PHYSICAL EXAMINATION

Blood pressure (mmHg)

		supine	sitting(5min)	standing(2min)
1	SBP			
	DBP (IV)*			
	DBP (V)			
2	SBP			
	DBP (IV)*			
	DBP (V)			

Sitting pulse rate (bpm) ☐☐☐

Weight (kg) ☐☐☐.☐

Fundoscopy — **Right eye** ☐ **Left eye** ☐

(stage Keith-Wagener)

1 = grade I, 2 = grade II, 3 = grade III, 4 = papilloedema,
5 = lens opacity, 8 = normal, 9 = unknown

Other physical signs ? ☐ 1 = yes, 2 = no

If yes, specify :

1 ....

2 ....

3 ....

4 ....

5 ....

*Optional

☐ for C.O.

ORIGINAL

[redacted]

[redacted]OOD TESTS

Was patient fasting ? ☐ 1 = yes, 2 = no, 9 = unknown

Test	Entry
Haematocrit (%)	☐☐ . ☐
Haemoglobin (mmol/L)	☐☐ . ☐
WBC (10^9 cells/L)	☐☐ . ☐
RBC (10^{12}cells/L)	☐ . ☐☐
Creatinine (μmol/L)	☐☐☐ . ☐
Uric acid (mmol/L)	☐ . ☐☐
Na (mmol/L)	☐☐☐ . ☐
K (mmol/L)	☐ . ☐☐
Glucose (mmol/L)	☐☐ . ☐
Cholesterol : — total (mmol/L)	☐☐ . ☐
— HDL (mmol/L)	☐☐ . ☐
Triglycerides (g/L)	☐☐ . ☐
Alk. phosph. (U/L)	☐☐☐
SGOT (U/L)	☐☐☐
SGPT (U/L)	☐☐☐
gGT (U/L)	☐☐☐
Insulin (U/ml)*	☐☐☐

[redacted]ECTROCARDIO[redacted]

ECG normal ? ☐ 1 = yes, 2 = no, 8 = not performed, 9 = unknown

If abnormal, specify : ....

....

....

Original sent to C.O. on ☐☐ day ☐☐ month ☐☐ year

If not sent, indicate reason : ....

....

....

Height of : calibration signal (1mv) ☐☐ (mm)

RaVL ☐☐ (mm)

SV_1 ☐☐ (mm)

RV_5 ☐☐ (mm)

Atrial fibrillation ☐ 1 = yes, 2 = no, 9 = unknown

URINE TESTS

Test	Entry	Codes
Proteinuria	☐	1 = positive, 2 = traces, 3 = negative, 9 = unknown
Glucosuria	☐	
White blood cells	☐	1 = many (≧ 10 field), 2 = rare, 3 = none, 9 = unknown
Red blood cells	☐	
Granular casts	☐	

Was urine sample sent for evaluation of compliance ?

— If yes, specify date : ☐☐ day ☐☐ month ☐☐ year

— If not, indicate reason, why not : ....

....

ACTIVITIES OF DAILY LIVING (ADL)

if "yes" score = 1 if "no" score = 0

Question	Score
Can the patient bath alone ?	☐
Can the patient dress alone ?	☐
Can the patient go to the toilet alone ?	☐
Can the patient move in and out bed and chair alone ?	☐
Is the patient entirely self-controlled for urination and defaecation ?	☐
Can the patient eat alone ?	☐

Total ADL score (sum of 6 scores) : ☐

* Optional ☐ for C.O.

ORIGINAL

SMOKING & DRINKING HABITS

Is patient smoking now ? 1 = yes, 2 = no, 9 = unknown

If yes, give consumption per **day** of :

cigarettes | cigars | pipes

Does the patient consume alcohol now ? 1 = yes, 2 = no, 9 = unknown

If yes, how many glasses per **week** of :

beer | wine

spirits | apéritif or fortified wine

DISEASES & FINDINGS

New diseases & findings since last visit ?
(including dementia)

1 ....

2 ....

3 ....

4 ....

5 ....

[illegible]evious diseases & findings still active ?
(including dementia)

1 ....

2 ....

3 ....

4 ....

5 ....

DECISIONS & COMMENTS

Does the patient continue supervised open follow-up ?

1 = yes, 2 = no, 9 = unknown

If no, specify reason :

....

....

....

If only non-supervised follow-up is possible, please indicate how information will be collected in the future :

— via General Practitioner

— via other hospital

— via population register

— via Office for Vital Statistics

— other

1 = yes, 2 = no, 9 = unknown

Please, specify name(s), addresse(s) & telephone number(s) of source(s) of information :

....

....

....

....

....

....

Comments :

....

....

....

for C.O.

ORIGINAL

SYST-EUR

SHORT REPORT DURING SUPERVISED OPEN FOLLOW-UP

IDENTIFICATION & REGISTRATION (1)

Syst-Eur patient number :

Date of visit : day month year

**Patient**	**Centre**
**Name or initials : (2)**	**Centre name :** ....
**Date of birth :** day month year	**Name of doctor : (2)** ....

INFORMATION ON TREATMENT

Drug 1

Name : .... /

Drug 2

Name : .... /

Drug 3

Name : .... /

Drug 4

Name : .... /

Drug 5

Name : .... /

Drug 6

Name : .... /

HISTORY

Did patient have any complaints since last visit ?

1 = yes, 2 = no, 9 = unknown

If yes, specify :

1 ....

2 ....

3 ....

4 ....

5 ....

acco D781D—1988

(1) **To be completed at quarterly intervals during supervised open follow-up.**
If a patient leaves the supervised open follow-up, a special form "Report at the End of Supervised Open Follow-up" should be completed.

2. Please print.

for C.O.

ORIGINAL

PHYSICAL EXAMINATION

Blood pressure (mmHg)

		supine	sitting(5min)	standing(2min)
1	SBP			
	DBP (IV)*			
	DBP (V)			
2	SBP			
	DBP (IV)*			
	DBP (V)			

Sitting pulse rate (bpm)

Other physical signs ? 1 = yes, 2 = no

If yes, specify :

1 ....

2 ....

3 ....

4 ....

5 ....

BLOOD TESTS**

WBC (10^9 cells/L) ▢▢.▢

K (mmol/L) ▢.▢▢

Creatinine (μmol/L) ▢▢▢.▢

DISEASES & FINDINGS

New diseases & findings since last visit ?
(including dementia)

1 ....

2 ....

3 ....

4 ....

5 ....

Previous diseases & findings still active ?
(including dementia)

1 ....

2 ....

3 ....

4 ....

5 ....

DECISIONS & COMMENTS

Does the patient continue supervised open follow-up ?(1)

1 = yes, 2 = no, 9 = unknown

If no, specify reason :

....

....

....

Comments :

....

....

....

* Optional

** Only compulsory during first year of ACE inhibition (otherwise optional)

(1) If a patient leaves the supervised open follow-up, a special form "Report at the End of Supervised Open Follow-up" should be completed.

▢ for C.O.

ORIGINAL

SYST-EUR

EXTENSIVE REPORT DURING SUPERVISED OPEN FOLLOW-UP

IDENTIFICATION & REGISTRATION (1)

Syst-Eur patient number : ☐☐☐ ☐ ☐☐☐ ☐☐

Date of visit : ☐☐ day ☐☐ month ☐☐ year

Patient	Centre
**Name or initials : (2)**	**Centre name :** ....
**Date of birth :** ☐☐ day ☐☐ month ☐☐ year	**Name of doctor : (2)** ....

INFORMATION ON TREATMENT

Drug 1

Name : .... ☐☐☐ / ☐☐☐☐☐

Drug 2

Name : .... ☐☐☐ / ☐☐☐☐☐

Drug 3

Name : .... ☐☐☐ / ☐☐☐☐☐

Drug 4

Name : .... ☐☐☐ / ☐☐☐☐☐

Drug 5

Name : .... ☐☐☐ / ☐☐☐☐☐

Drug 6

Name : .... ☐☐☐ / ☐☐☐☐☐

SMOKING & DRINKING HABITS

Is patient smoking now ? ☐ 1 = yes, 2 = no, 9 = unknown

If yes, give consumption per <u>day</u> of :

☐☐ cigarettes ☐☐ cigars ☐☐ pipes

Does the patient consume alcohol now ? ☐ 1 = yes, 2 = no, 3 = unknown

If yes, how many glasses per **week** of :

☐☐☐ beer ☐☐☐ wine

☐☐☐ spirits ☐☐☐ apéritif or fortified wine

ACTIVITIES OF DAILY LIVING (ADL)

if "yes" score = 1 if "no" score = 0

Can the patient bath alone ?	☐
Can the patient dress alone ?	☐
Can the patient go to the toilet alone ?	☐
Can the patient move in and out bed and chair alone ?	☐
Is the patient entirely self-controlled for urination and defaecation ?	☐
Can the patient eat alone ?	☐
**Total ADL score** (sum of 6 scores) :	☐

1. **To be completed at yearly intervals during supervised open follow-up.**
   If a patient leaves the supervised open follow-up, a special form "Report at the End of Supervised Open Follow-up" should be completed.
2. **Please print.**

HISTORY

Did patient have any complaints since last visit ?

☐ 1 = yes, 2 = no, 9 = unknown

If yes, specify :

1 .... ☐ ☐☐☐ . ☐

2 .... ☐ ☐☐☐ . ☐

3 .... ☐ ☐☐☐ . ☐

4 .... ☐ ☐☐☐ . ☐

5 .... ☐ ☐☐☐ . ☐

PHYSICAL EXAMINATION

Blood pressure (mmHg)

		supine	sitting(5min)	standing(2min)
1	SBP	☐☐☐	☐☐☐	☐☐☐
	DBP (IV)*	☐☐☐	☐☐☐	☐☐☐
	DBP (V)	☐☐☐	☐☐☐	☐☐☐
2	SBP	☐☐☐	☐☐☐	☐☐☐
	DBP (IV)*	☐☐☐	☐☐☐	☐☐☐
	DBP (V)	☐☐☐	☐☐☐	☐☐☐

Sitting pulse rate (bpm) ☐☐☐

Weight (kg) ☐☐☐ . ☐

Fundoscopy **Right eye** ☐ **Left eye** ☐

(stage Keith-Wagener)

1 = grade I, 2 = grade II, 3 = grade III, 4 = papilloedema,
5 = lens opacity, 8 = normal, 9 = unknown

Other physical signs ? ☐ 1 = yes, 2 = no

If yes, specify :

1 .... ☐ ☐☐☐ . ☐

2 .... ☐ ☐☐☐ . ☐

3 .... ☐ ☐☐☐ . ☐

4 .... ☐ ☐☐☐ . ☐

5 .... ☐ ☐☐☐ . ☐

URINE TESTS

Proteinuria ☐

Glucosuria ☐

1 = positive, 2 = traces, 3 = negative, 9 = unknown

White blood cells ☐

Red blood cells ☐

Granular casts ☐

1 = many (≧ 10 field), 2 = rare, 3 = none, 9 = unknown

BLOOD TESTS

Was patient fasting ? ☐ 1 = yes, 2 = no, 9 = unknown

Haematocrit (%)	☐☐ . ☐
Haemoglobin (mmol/L)	☐☐ . ☐
WBC (10^9 cells/L)	☐☐ . ☐
RBC (10^{12}cells/L)	☐ . ☐☐
Creatinine (μmol/L)	☐☐☐ . ☐
Uric acid (mmol/L)	☐ . ☐☐
Na (mmol/L)	☐☐☐ . ☐
K (mmol/L)	☐ . ☐☐
Glucose (mmol/L)	☐☐ . ☐
Cholesterol : — total (mmol/L)	☐☐ . ☐
— HDL (mmol/L)	☐☐ . ☐
Triglycerides (g/L)	☐☐ . ☐
Alk. phosph. (U/L)	☐☐☐
SGOT (U/L)	☐☐☐
SGPT (U/L)	☐☐☐
gGT (U/L)	☐☐☐
Insulin (U/ml)*	☐☐☐

*Optional ☐ for C.O. ORIGINAL

ELECTROCARDIOGRAM

ECG normal ? ☐ 1 = yes, 2 = no, 8 = not performed, 9 = unknown

If abnormal, specify : ....

....

....

Original sent to C.O. on ☐☐ day ☐☐ month ☐☐ year

If not sent, indicate reason : ....

....

.... ☐☐

Height of : calibration signal (1mV) ☐☐ (mm)

RaVL ☐☐ (mm)

SV1 ☐☐ (mm)

RV5 ☐☐ (mm)

Atrial fibrillation ☐ 1 = yes, 2 = no, 9 = unknown

DISEASES & FINDINGS

New diseases & findings since last visit ?
(including dementia)

1 ....
2 ....
3 ....
4 ....
5 ....

Previous diseases & findings still active ?
(including dementia)

1 ....
2 ....
3 ....
4 ....
5 ....

OPTIONAL EXAMINATIONS

Did the patient participate in one of the following side-projects ?

1. Quality of live assessment* ☐
2. 24-h BP recordings* ☐
3. Storage of serum* ☐
4. Multiple infarction dementia* ☐

1 = yes, 2 = no, 9 = unknown

DECISIONS & COMMENTS

Does the patient continue supervised open follow-up ?(1) ☐ 1 = yes, 2 = no, 9 = unknown

If no, specify reason :

....

....

....

Comments :

....

....

....

(1) If a patient leaves the supervised open follow-up, a special from "Report at the End of Supervised Open Follow-up" should be completed.

* Optional ☐ for C.O. ORIGINAL

SYST-EUR
REPORT AT THE END OF SUPERVISED OPEN FOLLOW-UP

IDENTIFICATION & REGISTRATION (1)

Syst-Eur patient number : ☐☐☐ ☐ ☐☐☐ ☐☐

Date of report : ☐☐ day ☐☐ month ☐☐ year

Patient

Name or initials : (2)

....

Date of birth :

☐☐ day ☐☐ month ☐☐ year

Centre

Centre name :

....

Name of doctor : (2)

....

DATE

Specify date of major fatal or non-fatal event, date of patient withdrawal, date on which patient defected from study or date of missed appointment :

☐☐ day ☐☐ month ☐☐ year

REASON

Specify reason why supervised open follow-up was ended :

Death ☐ 1 = yes, 2 = no

If yes, cause of death :

Immediate cause : ....

....

Underlying cause(s) : ☐ ☐☐☐ . ☐

1. .... ☐ ☐☐☐ . ☐

2. ....

Contributory cause(s) : ☐ ☐☐☐ . ☐

1. .... ☐ ☐☐☐ . ☐

2. .... ☐ ☐☐☐ . ☐

If yes, was autopsy performed, ☐
and
was protocol sent to C.O. ? ☐

1 = yes,
2 = no,
9 = unknown

Non-fatal events

1 = yes
2 = no

- **Cerebrovascular accident (excluding TIA)**
  - > cerebral haemorrhage ☐
  - > subarachnoid haemorrhage ☐
  - > thrombosis ☐
  - > embolism ☐
- **Retinal lesions**
  - > exudates or haemorrhage ☐
  - > papilloedema ☐
- **Myocardial infarction** ☐
- **Congestive heart failure,** requiring diuretics or vasodilators or any antihypertensive drug ☐
- **Dissecting aneurysm** ☐
- **Increase in serum creatinine** by 100 % or to 360 μmol/L (2mg/dl) at 2 consecutive visits ☐

Withdrawal

- **Interfering disease(s) or adverse drug effect(s)** ☐

  If yes, specify :

  1. .... ☐ ☐☐☐ . ☐
  2. .... ☐ ☐☐☐ . ☐

- **End of whole trial** ☐

Defector

Patient refuses to attend clinic : ☐

If yes, specify why :

1. .... ☐ ☐☐☐ . ☐
2. .... ☐ ☐☐☐ . ☐

Other reason(s) ☐

If yes, specify :

1. .... ☐ ☐☐☐ . ☐
2. .... ☐ ☐☐☐ . ☐

FOLLOW-UP

Has the patient been examined at clinic ?

☐ 1 = yes, 2 = no, 9 = unknown

If yes, please complete the following sections on pages 2 through 4.

(1) To be completed at end of supervised open follow-up.
(2) Please print.

☐ for C.O.

INFORMATION ON TREATMENT

Drug 1

Name : .... ☐☐☐ / ☐☐☐☐☐

Drug 2

Name : .... ☐☐☐ / ☐☐☐☐☐

Drug 3

Name : .... ☐☐☐ / ☐☐☐☐☐

Drug 4

Name : .... ☐☐☐ / ☐☐☐☐☐

Drug 5

Name : .... ☐☐☐ / ☐☐☐☐☐

Drug 6

Name : .... ☐☐☐ / ☐☐☐☐☐

SMOKING & DRINKING HABITS

Is patient smoking now ? ☐ 1 = yes, 2 = no, 9 = unknown

If yes, give consumption per <u>day</u> of :

☐☐ cigarettes ☐☐ cigars ☐☐ pipes

Does the patient consume alcohol now ? ☐ 1 = yes, 2 = no, 9 = unknown

If yes, how many glasses per <u>week</u> of :

☐☐☐ beer ☐☐☐ wine

☐☐☐ spirits ☐☐☐ apéritif or fortified wine

ACTIVITIES OF DAILY LIVING (ADL)

if "yes" score = 1 if "no" score = 0

Can the patient bath alone ? ☐

Can the patient dress alone ? ☐

Can the patient go to the toilet alone ? ☐

Can the patient move in and out bed and chair alone ? ☐

Is the patient entirely self-controlled for urination and defaecation ? ☐

Can the patient eat alone ? ☐

Total ADL score (sum of 6 scores) : ☐

HISTORY

Did patient have any complaints since last visit ?

☐ 1 = yes, 2 = no, 9 = unknown

If yes, specify :

1 .... ☐ ☐☐☐ . ☐

2 .... ☐ ☐☐☐ . ☐

3 .... ☐ ☐☐☐ . ☐

4 .... ☐ ☐☐☐ . ☐

5 .... ☐ ☐☐☐ . ☐

☐ for C.O.

ORIGINAL

PHYSICAL EXAMINATION

Blood pressure (mmHg)

		supine	sitting(5min)	standing(2min)
1	SBP			
	DBP (IV)*			
	DBP (V)			
2	SBP			
	DBP (IV)*			
	DBP (V)			

Sitting pulse rate (bpm)

Weight (kg) .

Fundoscopy — **Right eye** — **Left eye**

(stage Keith-Wagener)

1 = grade I, 2 = grade II, 3 = grade III, 4 = papilloedema,
5 = lens opacity, 8 = normal, 9 = unknown

Other physical signs ? 1 = yes, 2 = no

If yes, specify :

1 ....

2 ....

3 ....

4 ....

5 ....

URINE TESTS

Proteinuria

Glucosuria

(1 = positive, 2 = traces, 3 = negative, 9 = unknown)

White blood cells

Red blood cells

Granular casts

(1 = nany (≧ 10 field), 2 = rare, 3 = none, 4 = unknown)

BLOOD TESTS

Was patient fasting ? 1 = yes, 2 = no, 9 = unknown

Haematocrit (%)

Haemoglobin (mmol/L)

WBC (10^9 cells/L)

RBC (10^{12}cells/L)

Creatinine (μmol/L)

Uric acid (mmol/L)

Na (mmol/L)

K (mmol/L)

Glucose (mmol/L)

Cholesterol :
— total (mmol/L)

— HDL (mmol/L)

Triglycerides (g/L)

Alk. phosph. (U/L)

SGOT (U/L)

SGPT (U/L)

gGT (U/L)

Insulin (U/ml)*

*Optional

for C.O.

ORIGINAL

ELECTROCARDIOGRAM

ECG normal ? ☐ 1 = yes, 2 = no, 8 = not performed, 9 = unknown

If abnormal, specify : ....

....

....

Original sent to C.O. on ☐☐ day ☐☐ month ☐☐ year

If not sent, indicate reason : ....

....

....

Height of : calibration signal (1mV) ☐☐ (mm)

RaVL ☐☐ (mm)

SV_1 ☐☐ (mm)

RV_5 ☐☐ (mm)

Atrial fibrillation ☐ 1 = yes, 2 = no, 9 = unknown

DISEASES & FINDINGS

New diseases & findings since last visit ?
(including dementia)

1 ....

2 ....

3 ....

4 ....

5 ....

Previous diseases & findings still active ?
(including dementia)

1 ....

2 ....

3 ....

4 ....

5 ....

DECISIONS & COMMENTS

Please indicate how information will be collected during non-supervised open follow-up ?

— via General Practitioner ☐

— via other hospital ☐

— via population register ☐

— via Office for Vital Statistics ☐

— other ☐

1 = yes,
2 = no,
9 = unknown

Please, specify name(s), addresse(s) & telephone number(s) of source(s) of information :

....

....

....

....

....

....

Comments :

....

....

....

* Optional ☐ for C.O. ORIGINAL

SYST-EUR
ANNUAL REPORT DURING NON-SUPERVISED OPEN FOLLOW-UP

IDENTIFICATION & REGISTRATION (1)

Syst-Eur patient number : ☐☐☐ ☐ ☐☐☐ ☐☐

Date of visit : ☐☐ day ☐☐ month ☐☐ year

Patient

Name or initials : (2)

....

Date of birth :

☐☐ day ☐☐ month ☐☐ year

Centre

Centre name :

....

Name of doctor : (2)

....

VITAL STATUS

Is patient deceased ? ☐ 1 = yes, 2 = no, 9 = unknown

If patient is deceased, please specify :

date of death ☐☐ day ☐☐ month ☐☐ year

Cause(s) of death :

Immediate cause . ...

....
☐ ☐☐☐ . ☐

Underlying cause(s) :

1 ....
☐ ☐☐☐ . ☐

....
☐ ☐☐☐ . ☐

Contributory cause(s) :

1 ....
☐ ☐☐☐ . ☐

2 ....
☐ ☐☐☐ . ☐

If patient is alive, please indicate whether he (she) is currently taking antihypertensive drugs :

☐ 1 = yes, 2 = no, 9 = unknown

DISEASES & FINDINGS

New diseases & findings since last report ?
(including dementia)

1 ....
☐ ☐☐☐ . ☐

2 ....
☐ ☐☐☐ . ☐

3 ....
☐ ☐☐☐ . ☐

4 ....
☐ ☐☐☐ . ☐

5 ....
☐ ☐☐☐ . ☐

Previous diseases & findings still active ?
(including dementia)

1 ....
☐ ☐☐☐ . ☐

2 ....
☐ ☐☐☐ . ☐

3 ....
☐ ☐☐☐ . ☐

4 ....
☐ ☐☐☐ . ☐

5 ....
☐ ☐☐☐ . ☐

COMMENTS

....

....

....
☐ ☐☐☐ . ☐/☐ ☐☐☐ . ☐/☐ ☐☐☐ . ☐

acco D781G—1988

(1) To be completed at yearly intervals during non-supervised open follow-up, i.e. in patients who do not continue to attend the clinic.

(2) Please print

☐ for CO

ORIGINAL

SYST-EUR

REPORT ON 24-HOUR BLOOD PRESSURE

IDENTIFICATION & REGISTRATION (1)

Syst-Eur patient number : ☐☐☐ ☐ ☐☐☐ ☐☐

Patient

Name or initials : (2) ....

Date of birth : ☐☐ (day) ☐☐ (month) ☐☐ (year)

Centre

Centre name : ....

Name of doctor : (2) ....

INFORMATION ON RECORDING (3)

Phase of study ☐
1 = run-in
2 = double-blind
3 = open follow-up

START

Date ☐☐ (day) ☐☐ (month) ☐☐ (year)

Hour ☐☐ (hour) ☐☐ (min.)

END

Date ☐☐ (day) ☐☐ (month) ☐☐ (year)

Hour ☐☐ (hour) ☐☐ (min.)

Type of recorder . ...

....
☐☐

Arm to which cuff was secured ☐
1 = right,
2 = left,
9 = unknown

Arm circumference ☐☐ cm

Bladder size ☐☐ (width) X ☐☐ (length) cm

STUDY MEDICATION DURING RECORDING (3)

Hypotensor 1111 (run-in period)

Dose ? (tablets/day) ☐ . ☐

Hour(s) of intake

— morning ☐☐ h. ☐ min.

— evening ☐☐ h. ☐ min.

Hypotensor A

Dose ? (tablets/day) ☐ . ☐

Hour(s) of intake

— morning ☐☐ h. ☐ min.

— evening ☐☐ h. ☐ min.

Hypotensor B

Dose ? (tablets/day) ☐ . ☐

Hour(s) of intake

— morning ☐☐ h. ☐ min.

— evening ☐☐ h. ☐ min.

Hypotensor C

Dose ? (tablets/day) ☐ . ☐

Hour(s) of intake

— morning ☐☐ h. ☐ min.

— evening ☐☐ h. ☐ min.

(1) This report should be completed for each 24 h. blood pressure recording.
(2) Please print.
(3) Please, give hours in figures from 00 tot 24.

acco D2318H—1989

☐ for C.O.

ORIGINAL

ANY OTHER DRUGS WITH POTENTIAL INFLUENCE ON BLOOD PRESSURE ?

Drug 1

Name ....

☐☐☐/☐☐☐☐☐

Drug 2

Name ....

☐☐☐/☐☐☐☐☐

Drug 3

Name ....

☐☐☐/☐☐☐☐☐

SYMPTOMS AND COMPLAINTS DURING RECORDING(1)

Did the patient note any of the following symptoms or complaints in his/her diary ?

☐ 1 = yes, 2 = no, 9 = unknown

If yes, specify symptom code and moment of occurrence :

Symptom code :
- 1 = dizziness, light-headedness, weakness, or equivalent
- 2 = fatigue, tiredness, or equivalent
- 3 = visual disturbances
- 4 = headache
- 5 = flushing, facial erythema
- 6 = nausea, vomiting
- 7 = palpitations, extrasystoles, tachycardia
- 8 = syncope

1. Symptom code ☐ Moment ☐☐ h. ☐☐ min.

2. Symptom code ☐ Moment ☐☐ h. ☐☐ min.

3. Symptom code ☐ Moment ☐☐ h. ☐☐ min.

4. Symptom code ☐ Moment ☐☐ h. ☐☐ min.

5. Symptom code ☐ Moment ☐☐ h. ☐☐ min.

---

(1) Please, give hours in figures from 00 to 24.

☐ for C.O.

ORIGINAL

MAIN MEAL DURING RECORDING (1)

☐☐ h. ☐☐ min.

SLEEP AT NIGHT (1)

START ☐☐ h. ☐☐ min.

(time patient went to sleep)

END ☐☐ h. ☐☐ min.

(first visual sensation after sleep)

BLOOD PRESSURE AT START OF RECORDING

Office blood pressure (mmHg)

		sitting (5min)
1	SBP	☐☐☐
	DBP (IV)(2)	☐☐☐
	DBP (V)	☐☐☐

		sitting (5min)
2	SBP	☐☐☐
	DBP (IV)(2)	☐☐☐
	DBP (V)	☐☐☐

DATA MANAGEMENT

Did you send the recording of the ambulatory BP to the C.O. ?

☐ 1 = yes, 2 = no

— If yes, specify date : ☐☐ day ☐☐ month ☐☐ year

— If yes, specify how ? ☐ 1 = File on diskette, 2 = ASCII file via EARN, 3 = ASCII file via modem,

— If not, indicate how the data will be sent :

....

....

....

☐☐

COMMENTS

....

....

....

☐☐☐☐.☐/☐☐☐☐.☐/☐☐☐☐.☐

(1) Please, give hours in figures from 00 to 24.
(2) Where Korotkoff sounds persist below 50 mmHg, please give also phase IV DBP.

☐ for C.O. ORIGINAL

SYST-EUR

"MINI-MENTAL STATE" EXAMINATION (MMS)

IDENTIFICATION & REGISTRATION (1)

Syst-Eur patient number : ☐☐☐ ☐ ☐☐☐ ☐☐

Date of visit : ☐☐ day ☐☐ month ☐☐ year

Phase of study : ☐ 1 = run-in, 2 = double-blind, 3 = open follow-up

Patient	Centre
**Name or initials : (2)**	**Centre name :** ....
**Date of birth :** ☐☐ day ☐☐ month ☐☐ year	**Name of doctor : (2)** ....

A. ORIENTATION (Ask the following questions)

0 = incorrect
1 = correct
8 = not performed

Question	Item	Score
What is today's date ?	Date	☐
What is the year ?	Year	☐
What is the month ?	Month	☐
What day is today ?	Day	☐
Can you also tell me what season it is ?	Season	☐
Can you also tell me the name of this hospital (clinic) ?	Hospital	☐
What floor are we on ?	Floor	☐
What town or city are we in ?	Town	☐
What county, state or department are we in ?	County	☐
What country are we in ?	Country	☐

B. IMMEDIATE RECALL

Ask the subject if you may test his/her memory. Then say : "**ball**", "**flag**", "**tree**", clearly and slowly. (one second for each word). After you have said all three words, ask him/her to repeat them. The first repetition determines his/her score (0-3) but keep saying them until he/she can repeat all three, up to six tries. If he/she does not eventually learn all three, recall (D) cannot be meaningfully tested.

Ball ☐
Flag ☐
Tree ☐

C. ATTENTION AND CALCULATION

1. Ask the subject to begin with 100 and count backwards by 7. Stop after five subtractions (93, 86, 79, 72, 65). Score the total numbers of correct answers.

   93 ☐
   86 ☐
   79 ☐
   72 ☐
   65 ☐

If the subject cannot or will not perform the "count backwards test" task, then proceed to C2.

2. Ask the subject to spell the word "**world**" backwards. The score is the number of letters in correct position. For example, "**dlrow**" is 5, "**dlorw**" is 3.

   d ☐
   l ☐
   r ☐
   o ☐
   w ☐

(1) To be completed at end of the run-in period, at yearly intervals during double-blind treatment, at end of double-blind treatment, at yearly intervals during open follow-up and at end of open follow-up.
(2) Please print.

ORIGINAL

D. RECALL

0 = incorrect
1 = correct
8 = not performed

Ask the subject to recall the three words you previously asked him/her to remember (B).

Ball	
Flag	
Tree	

E. LANGUAGE

Naming

— Show the subject a wrist watch and ask him/her what it is.
— Repeat for pencil.

Watch	
Pencil	

Repetition

Ask the subject to repeat "**No ifs, ands, or buts**"

Repetition	

3-Stage command

Give the subject a piece of plain blank paper and say :

"**Take the paper in your right hand, fold it in half, and put it on the floor**".

Takes paper in right hand	
Folds paper in half	
Puts paper on floor	

Reading

Hold up the card which reads "**Close your eyes**", so the subject can see it clearly. Ask him/her to read it and to do what it says. Score as correct only if he/she actually closes his/her eyes.

Closes eyes	

Writing

Give the subject a blank piece of paper provided and ask him/her to write a sentence. It is to be written spontaneously. It must contain a subject and a verb and be sensible. Correct grammar and punctuation are not necessary.

Writes sentence	

Copying

On a clean piece of paper, draw intersecting pentagons, each side about 2 cm (as shown in the example below), and ask the subject to copy the drawing exactly as it is. All 10 angles must be present and two must intersect to score one point.
Tremor and rotation are ignored.

Draws pentagons	

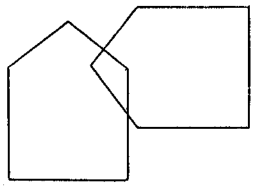

DERIVING TOTAL SCORE

Sum the number of correct replies to the test items.
If item (count backwards) was not performed and if instead item C2 (world spelled backward) was used then add the number of correct letters given in proper position (zero to five).

Total Score (1)
(Maximum 30)

(1) If total score is 23 points or less, further diagnostic evaluation is necessary. (See section C2 of protocol "Vascular Dementia side project")

ORIGINAL

SURGERY CODES

S 1000	Radiotherapy

Gynaecological surgery

S 1102	Subtotal hysterectomia
S 1103	Total hysterectomia
S 1106	Laparotomia for ligamentopexia or Douglas punction
S 1111	Extensive hysterectomia (Wertheim)
S 1113	Surgery for genital prolaps
S 1115	Ovariectomia or other surgery on ovaria (1 or 2 sides)
S 1119	Surgery on Bartholin gland
S 1121	Extensive hysterectomia with pelvical lymphadenectomia
S 1139	Cystoscopia (± biopsia)
S 1146	Adnexectomia (1 or 2 sides)
S 1149	Insufflation of tuba and/or injection of therapeutical product for hysterosalphingographia
S 1156	Myomectomia
S 1165	Surgery on tuba ovarii (including sterilisation)
S 1169	Dilatation of cervix uteri
S 1177/ S 1186	Colporraphia and colpoperineoraphia
S 1189	Surgery on vagina and/or vulva
S 1195	Salphingectomia and salphingostomia
S 1197	Hysterotomia with polypectomia
S 1199	Surgery on vaginal cyst
S 1202	Laparotomia or laparoscopia because of sterilisation
S 1225	Surgery for urinary incontinence
S 1235	Hysterotomia
S 1999	Gynaecological surgery, NEC

S 1600	Dental extraction

Surgery on eyes

S 1802	Dacryocystorhinostomia
S 1803	Dilaceration of lacrimal ducts
S 1811	Exceresis of tumor of eye lid
S 1815	Exceresis of chalazion
S 1820	Plastic surgery on eye lids
S 1827	Other surgery on eye lids, NEC
S 1846	Grafting of coniunctiva
S 1860	Surgery on perforating wound of eye ball
S 1862	Iridectomia
S 1863	Glaucoma operation
S 1864	Extraction of eye lens ± intra-ocular implantation of lens
S 1865	Secundary cataract
S 1866	Replacement of vitrious body
S 1867	Photocoagulation therapy on eye (with xenonlamp or laser)
S 1869	Surgery for retinal detachment
S 1878	Extraction of eye lens + complete surgical glaucoma treatment
S 1881	Enucleation or evisceration of eye ball
S 1895	Surgery for strabismus or nystagmus
S 1901	Eye operation - unspecified
S 1906	Alcoholisation of ganglion ciliare

General surgery

S 2020	Surgery on deep phlegmone
S 2029	Surgery for papilloma or condyloma
S 2030	Curettage of uterus
S 2036	Exceresis of deep, malignant tumor of internal organs
S 2046	Surgery on deep, malignant tumor of face, lips, etc. (extensive resection)
S 2078	Surgery on deep, benignant tumor or non-traumatic leasion (minus skin leasions)
S 2079	Surgery on benignant or malignant superfiscial tumors or non-traumatic leasions
S 2089	Exceresis of cold abscess

Neurosurgery

S 2125 Sympathectomia
S 2138 Ventriculo-subcutaneous drainage
S 2146 Trepanation (decompression or drainage)
S 2150 Surgery for spinal stenosis
S 2171 Internal drainage of hydrocephalus

Plastic surgery

S 2200 Face lift
S 2228 Dermo-epidermal graft
S 2240 Skin transplantation
S 2244 Breast reconstruction after total mammectomia for tumor

---

Abdominal surgery

S 2300 Surgery for hiatus hernia or hernia of diaphragma
S 2301 Surgery for incarcerated eventration or hernia
S 2303 Surgery for eventration or hernia without incarceration
S 2308 Surgery for omphalocoele
S 2311 Exceresis of excessive abdominal adiposal tissue
S 2313 Fissurectomia
S 2321 Exceresis of benignant tumor of stomach
S 2332 Total gastrectomia with oesophago-jejunal anastomosis or subtotal gastrectomia with repair of transit by interposition of bowel segment
S 2333 Subtotal gastrectomia
S 2334 Antrectomia with vagotomia
S 2335 Vagotomia
S 2336 Resection of stomach without discontinuation
S 2337 Degastro-gastrectomia
S 2341 Pylorotomia or pyloroplastia
S 2343 Gastrotomia
S 2350 Duodeno-pancreatectomia
S 2356 Repair of duodenal fistula
S 2363 Surgery on abscess of liver
S 2368 Cholecystectomia
S 2369 Operation on biliary ducts

S 2373	Choledochotomia (± cholecystectomia)
S 2374	Cholecystostomia
S 2375	Surgery on sfincter of Oddi or papilla Vateri with duodenotomia
S 2377	Splenectomia
S 2401	Total colectomia
S 2402	Hemocolectomia
S 2403	Segmentar colectomia
S 2407	Appendectomia
S 2408	Anus praeter
S 2411	Segmentary resection of small bowel
S 2414	Resection of benignant tumor of bowel by enterotomia
S 2416	Laparotomia because of bowel obstruction
S 2432	Explorative laparotomy
S 2434	Surgery on pelvical or mesocoliacal abscess
S 2437	Exceresis of tumor of mesenterium
S 2450	Abdomino-perineal amputation of rectum
S 2452	Hartmann operation
S 2457	Operation for rectal prolapsus
S 2467	Resection of benignant tumors or polyps of rectum (endoscopic)
S 2468	Resection of benignant tumors or polyps of sigmoïd (endoscopic)
S 2471	Resection of anal fistula
S 2476	Hemorroidal operation
S 2480	Resection of anal abscess

Thoracal surgery

S 2500	Extensive breast amputation
S 2504	Subtotal mammectomy or resection of breast tumor
S 2505	Clearance of axillar lymph glands
S 2507	Biopsia on mamma
S 2511	Thoracoplastia
S 2514	Pneumotomia or cavernostomia
S 2515	Pleuropneumonectomia, pleurolobectomia, costopleuropneumonectomia because of chronic pleuritis
S 2518	Total or subtotal exceresis of lung
S 2526	Surgical release of lung with collaps of lung, apicolyse, extrapleural pneumothorax, intrapleural pneumolysis with open thorax

S 2528	Explorative thoracotomia
S 2550	Operation on heart with extracorporal circulation
S 2551	Operation on large intrathoracal arteries with extracorporal circulation
S 2552	Open heart operation (hypothermia)
S 2555	CABG and PTCA
S 2556/ S 2557	Pacemaker implantation
S 2565	Coarctation of aorta

Vascular surgery

S 2601	Arteriectomia of arteria axillaris, femoralis, poplitea
S 2603	Revascularisation of arteria carotis or vertebralis
S 2604	Revascularisation of arteries of limbs
S 2606	Embolectomia or thrombectomia via arteries of limbs or neck
S 2609	Drainage of edema (elephantiasis)
S 2615	Revascularisation of large intrathoracal artery
S 2618	Surgery on aorta, arteria anonyma, arteria subclavia, arteria carotis
S 2625	Surgery because of elephantiasis
S 2629	Revascularisation of abdominal artery
S 2632	Grafting of wounds or incissions of large intra-abdominal arteries
S 2640	Tie-up of intra-abdominal vena cava
S 2642	thrombectomia of deep veins of limbs
S 2643	Varices operation
S 2646	Total exceresis of vena saphena externa
S 2647	Resection of vena saphena interna

ORL surgery

S 2801	Functional surgery on bones in middle ear and operation for fenestration
S 2805	Mastoïditis operation
S 2808	Antrotomia
S 2820	Operation on nasal synechia
S 2827	Total or subtotal parotidectomia
S 2830	Partial or total resection of nasal septum or nasal shell
S 2840	Total or partial thyroidectomia
S 2842	Endoscopic treatment on chordae vocales

S 2853	Surgery because of petromastoiditis
S 2863	Labyrinthal trepanation because of petrositis or Menière disease
S 2869	Surgery because of othematoma
S 2870	Surgery because of rhinophyma
S 2877	Surgery because of sinusitis
S 2897	Ethnoidectomia
S 2907	Arnygdalectomia and adenoidectomia
S 2916	Tympanoplastia
S 2918	Surgery on tumor or cyste in neck
S 2920	Exceresis of laryngal polyps
S 2923	Surgery on internal ear
S 2929	Extraction of saliva stones
S 2980	Uvuloplastia
S 2983	Oesophagotomia
S 2987	Resection of styloid apophyses

Urology

S 3006	Cystostomia
S 3003	Nephromtomia, with or without removal of stones
S 3017	Lithotriptia
S 3019	Cystoscopia (men)
S 3023	Surgery for hydronephrosis
S 3024	Prostatectomia (surgery or endoscopy)
S 3026	Surgery for urinary incontinence (surgery on bladder neck)
S 3027	Epididimectomia
S 3028	Surgery for varicocoele
S 3029	Cystoscopia with ureter catheterisation
S 3043	2-sided uretero-intestinal anastomosis
S 3045	Pyelotomia
S 3059	Tie-up of ductus deferens
S 3060	Dilatation of ureter
S 3067	Resection of tunica vaginalis
S 3073	Surgery because of bladder malformations
S 3083	Resection of bladder tumor
S 3085/ S 3087	Ureterotomia

S 3097	Castration
S 3102	Nephro- or pyelotomia because of lithiasis
S 3109	Total nephrectomia
S 3114	Subtotal nephrectomia
S 3116	Surgery for renal cyst
S 3130	Dilatation of urethra
S 3149	Distraction of kidney stone by ultrasonic waves (percutaneous)

Orthopedic surgery

S 9000	Removal of synthetic material
S 9001	Bone biopsy
S 9002	Bone surgery unspecified S 9003 : arthroscopy
S 9024	Tenotomia
S 9030	Attachment of tendon
S 9040	Reposition of luxation, fracture or both of cervical vertebral colum
S 9041	Cervical osteosynthesis
S 9042	Cervical orthrodesia
S 9060	Reposition of luxation, fracture or both of lumbosacral vertebral colum
S 9061	Vertebral osteosynthesis
S 9062	Vertebral arthrodesia
S 9070	Laminectomia
S 9071	Laminectomia with or without arthrodesis
S 9072	Flavoligamentectomia
S 9073	Surgery for discal hernia with or without arthrodesis
S 9082	Exceresis of tumor from vertebra
S 9083	Resection and reconstruction of 1 or more vertebrae
S 9128	Sternoclavicular arthrodesia
S 9129	Treatment of scapulohumeral luxation
S 9132	Scapulohumeral arthrotomia
S 9158	Surgery on congenital scapula elevata
S 9159	Surgical treatment of fracture of humerus
S 9184	Arthrotomia or capsulotomia of elbow
S 9191	Resection or hemiresection of elbow
S 9198	Surgery on fracture of distal end of lower arm
S 9218	Exceresis of intramuscular osteoma of elbow

S 9219	Amputation of distal part of the arm
S 9232	Synovectomia of wrist
S 9233	Arthorplastia of wrist
S 9248	Amputation of finger(s)
S 9280	Metacarpophalyngeal synovectomia of finger(s)
S 9290	Surgery on rupture of capsule of rotator muscles of shoulder
S 9291	Surgery for rupture, tenosynovitis or luxation of a tendon of the biceps
S 9294	Exceresis of calcifications of capsule of rotator muscles of shoulder
S 9306	Total or subtotal aponeurotomia of handpalm or fingers
S 9309	Complete or partial aponeurectomia of handpalm or fingers
S 9324	Surgery for de Quervain disease
S 9325	Tenolysis of flexor muscles of upper limbs
S 9330	Surgery on canalis carpi
S 9360	Surgical treatment of fracture of hip (including acetabulum)
S 9369	Arthrotomia of the hip
S 9370	Removal of free body from hip articulation
S 9371	Surgery because of osteochondritis dissecans of the hip
S 9372	Capsulectomia of hip
S 9373	Synovectomia of the hip
S 9374	Arthroplastia of hip
S 9376	Arthroplastia of hip with prosthesis f.i. hipprosthesis
S 9380	Arthrodesia (not in hip articulation)
S 9388	Surgical treatment of femur fracture
S 9406	Surgery on periost of femur
S 9407	Osteotomia of femur
S 9419	Treatment for tumor of femur with grafting
S 9422	Amputation of femur
S 9426	Arthrotomia of the knee
S 9428	Exceresis of meniscus
S 9429	Capsulotomia of knee
S 9430	Total synovectomia of the knee
S 9431	Arthrolysis of the knee
S 9432/ S 9434	Femoropatellar arthroplastia
S 9437	Femorotibial arthroplastia with prosthesis
S 9440	Resection of knee

S 9441	Athrorisis of the knee
S 9446	Resection of patella
S 9447	Surgical treatment of fracture of patella
S 9450	Surgery for fracture of tibia, fibula or both
S 9463	Surgical treatment of pseudo arthosis of patella
S 9468	Osteotomia of tibiadiafyse, fibuladiafyse or both
S 9470	Osteotomia of tibia plateau
S 9488	Amputation of lower leg
S 9517	Osteotomia of bones of the foot (not metatarsals or phalanges)
S 9542	Metatarsophalangeal alignement (operation of Lelièvre)
S 9543	Amputation of foot
S 9548	Tarsometatarsal synovectomia
S 9549	Tarso-metatarsal arthroplastia
S 9563	Amputation of toe
S 9568	Metatarsophalangeal of interphalangeal arthrotomia
S 9573	Interphalangeal or metatarsophalangeal arthroplastia on 1 or more toes
S 9586	Surgery for hallux valgus
S 9594	Surgery on ingrowing toe nail
S 9616	Attachment of quadriceps tendon
S 9632	Attachment of tendon in feet
S 9638	Tenotomia of Achilles tendon
S 9640	Lenghtening or shortening of tendon of leg or foot

ClinicalTrials.gov
A service of the U.S. National Institutes of Health

Trial record **1 of 4** for: Systolic Hypertension in Europe Trial
Previous Study | Return to List | Next Study

Systolic Hypertension in Europe (Syst-Eur)

This study has been terminated.

Sponsor:
Katholieke Universiteit Leuven

Information provided by (Responsible Party):
Jan A. Staessen, Katholieke Universiteit Leuven

ClinicalTrials.gov Identifier:
NCT02088450

First received: March 10, 2014
Last updated: March 13, 2014
Last verified: March 2014
History of Changes

Full Text View | Tabular View | No Study Results Posted | Disclaimer | How to Read a Study Record

Purpose

The Syst-Eur **Trial** is a concerted action of the European Community's Medical and Health Research Programme. The **trial** is carried out in consultation with the World Health Organization, the International Society of **Hypertension**, the European Society of **Hypertension** and the World **Hypertension** League. Syst-Eur is a multicentre **trial** designed by the European Working Party on **High Blood Pressure** in the Elderly (EWPHE), to test the hypothesis that antihypertensive treatment of elderly patients with isolated **systolic hypertension** results in a significant change in stroke morbidity and mortality. Secondary endpoints include cardiovascular events, such as myocardial infarction and congestive heart failure. To be eligible patients must be at least 60 years old and have a **systolic** blood pressure averaging 160-219 mmHg with a diastolic pressure less than 95 mmHg. Patients must give their informed consent and be free of major cardiovascular and non-cardiovascular diseases at entry. The patients are randomized to active treatment or placebo. Active treatment consists of nitrendipine (10-40 mg/day), combined with enalapril (5-20 mg/day) and hydrochlorothiazide (12.5-25 mg/day), as necessary. The patients of the control group receive matching placebos. The drugs (or matching placebos) are stepwise titrated and combined in order to reduce **systolic** blood pressure by 20 mmHg at least to a level below 150 mmHg. Morbidity and mortality are monitored to enable an intention-to-treat and per-protocol comparison of the outcome in the 2 treatment groups.

Condition	Intervention	Phase
Isolated **Systolic Hypertension**	Drug: Active treatment with nitrendipine (10-40 mg/day).	Phase 2

Study Type: Interventional
Study Design: Allocation: Randomized
Intervention Model: Parallel Assignment
Masking: Double Blind (Subject, Caregiver, Investigator, Outcomes Assessor)
Primary Purpose: Treatment

Official Title: **Systolic Hypertension in Europe Trial**

Resource links provided by NLM:

MedlinePlus related topics: Heart Attack Heart Failure High Blood Pressure Stroke

Drug Information available for: Hydrochlorothiazide Enalapril Enalapril maleate

U.S. FDA Resources

Further study details as provided by Katholieke Universiteit Leuven:

Primary Outcome Measures:

- Incidence of stroke [Time Frame: 3-monthly visits up to 5 years]

Secondary Outcome Measures:

- Death [Time Frame: 3-monthly visits up to 5 years]
- Myocardial infarction [Time Frame: 3-monthly visits up to 5 years]
- Congestive heart failure [Time Frame: 3-monthly visits up to 5 years]

Enrollment: 4695
Study Start Date: February 1990
Study Completion Date: February 1997
Primary Completion Date: February 1997 (Final data collection date for primary outcome measure)

Arms	Assigned Interventions
Active Comparator: Active treatment<br>Active treatment with nitrendipine (10-40 mg/day). If necessary, the dihydropyridine calcium-channel blocker was combined with or replaced by enalapril maleate (5-20 mg/day), hydrochlorothiazide (12.5-25 mg/day), or both drugs.	Drug: Active treatment with nitrendipine (10-40 mg/day).<br>If necessary, the dihydropyridine calcium-channel blocker was combined with or replaced by enalapril maleate (5-20 mg/day), hydrochlorothiazide (12.5-25 mg/day), or both drugs.
Placebo Comparator: Placebo<br>Placebo tablets were identical to the study drugs with a similar schedule.	

Eligibility

Ages Eligible for Study: 60 Years and older
Genders Eligible for Study: Both
Accepts Healthy Volunteers: No

Criteria
Inclusion Criteria:

- At least 60 years old.
- Sitting systolic blood pressure on masked placebo, during run-in phase of 160 to 219 mmHg, with a sitting diastolic blood pressure below 95 mmHg and a standing systolic blood pressure of at least 140 mmHg
- Informed consent must be obtained

Exclusion Criteria:

- Systolic hypertension is secondary to a disorder that needed specific medical or surgical treatment.
- Retinal haemorrhage or papilloedema;
- Congestive heart failure
- Dissecting aortic aneurysm
- Serum creatinine concentration at presentation of 180μmol/L or more.
- History of severe nose bleeds.
- Stroke or myocardial infarction in the year before the study.
- Dementia.
- Substance abuse.
- Any disorder prohibiting a sitting or standing position.
- Any severe concomitant cardiovascular or non-cardiovascular disease.

Contacts and Locations

No Contacts or Locations Provided

More Information

No publications provided

Responsible Party: Jan A. Staessen, Professor of Medicine, Katholieke Universiteit Leuven
ClinicalTrials.gov Identifier: NCT02088450 History of Changes
Other Study ID Numbers: Syst-Eur
Study First Received: March 10, 2014
Last Updated: March 13, 2014
Health Authority: Belgium: Institutional Review Board

Keywords provided by Katholieke Universiteit Leuven:
Cardiovascular complications
Clinical **trial**
Elderly
Isolated **systolic hypertension**
Stroke

Additional relevant MeSH terms:
Hypertension
Vascular Diseases
Cardiovascular Diseases
Calcium Channel Blockers
1,4-dihydropyridine
Nitrendipine
Enalapril
Hydrochlorothiazide
Membrane Transport Modulators
Cardiovascular Agents
Therapeutic Uses
Angiotensin-Converting Enzyme Inhibitors
Protease Inhibitors
Enzyme Inhibitors
Antihypertensive Agents
Diuretics
Natriuretic Agents
Physiological Effects of Drugs

Molecular Mechanisms of Pharmacological Action	Sodium Chloride Symporter Inhibitors
Pharmacologic Actions	Vasodilator Agents

ClinicalTrials.gov processed this record on April 29, 2014
